# Targeting the G-quadruplex as a novel strategy for developing antibiotics against hypervirulent drug-resistant *Staphylococcus aureus*

**DOI:** 10.1186/s12929-024-01109-3

**Published:** 2025-02-05

**Authors:** Maria Sultan, Maria Razzaq, Joohyun Lee, Shreyasi Das, Shrute Kannappan, Vinod Kumar Subramani, Wanki Yoo, Truc Kim, Hye-Ra Lee, Akhilesh K. Chaurasia, Kyeong Kyu Kim

**Affiliations:** 1 Department of Precision Medicine, Graduate School of Basic Medical Science, Institute for Antimicrobial Resistance Research and Therapeutics, Sungkyunkwan University School of Medicine, Suwon, 16419 Republic of Korea; 2 Department of Biotechnology and Bioinformatics, College of Science and Technology, Korea University, Sejong, 30019 Republic of Korea

**Keywords:** *S. aureus* USA300, G-quadruplex, G4 ligands, N-methyl mesoporphyrin IX (NMM), Division cell wall, *dcw* cluster, *mraZ*

## Abstract

**Background:**

The rapid emergence of multiple drug-resistant (MDR) bacterial pathogens and the lack of a novel antibiotic pipeline pose a serious threat to global healthcare. The limited number of established targets further restricts the identification of novel antibiotics to treat life-threatening MDR infections caused by *Staphylococcus aureus* strains. Therefore, novel targets for developing antibiotics are urgently required. In this study, we hypothesized that the G-quadruplex (G4)-binding ligands can be used as novel antibiotics as their binding can possibly downregulate/block the expression of vital genes.

**Methods:**

To test this, first we screened the antibiotic properties of representative G4-binding ligands against hypervirulent and MDR *S. aureus* USA300 and determined the in vitro and in vivo antibacterial activity; and proposed the mechanism of action by applying various microbiological, infection, microscopic, and biophysicochemical techniques.

**Results:**

Herein, among screened G4-binding ligands, N-methyl mesoporphyrin IX (NMM) showed the highest antibacterial activity against *S. aureus* USA300. NMM exhibited a minimum inhibitory concentration (MIC) of 5 μM against *S. aureus* USA300, impacting cell division and the cell wall by repressing the expressions of genes in the division cell wall (*dcw*) gene cluster. Genome-wide bioinformatics analysis of G4 motifs and their mapping on *S. aureus* genome, identified the presence of G4-motif in the promoter of *mraZ*, a conserved master regulator of the *dcw* cluster regulating the coordinated cell division and cell wall synthesis. Physicochemical assessments using UV–visible, circular dichroism, and nuclear magnetic resonance spectroscopy confirmed that the G4-motif present in the *mraZ* promoter formed an intramolecular parallel G4 structure, interacting with NMM. In vivo reporter followed by coupled in vitro transcription/translation (IVT) assays confirmed the role of *mraZ* G4 as a target interacting NMM to impose extreme antibacterial activity against both the gram-positive and -negative bacteria. In-cell and in vivo validation of NMM using RAW264.7 cells and *Galleria mellonella*; respectively, demonstrated that NMM exhibited superior antibiotic activity compared to well-established antibiotics, with no observed cytotoxicity.

**Conclusions:**

In summary, the current study identified NMM as a broad-spectrum potent antibacterial agent and elucidated its plausible mechanism of action primarily by targeting G4-motif in the *mraZ* promoter of the *dcw* gene cluster.

**Supplementary Information:**

The online version contains supplementary material available at 10.1186/s12929-024-01109-3.

## Background

Methicillin-resistant *Staphylococcus aureus* (MRSA) has rapidly evolved into a dangerous pathogen, prompting the World Health Organization (WHO) to classify *S. aureus* as a priority pathogen due to its notable global morbidity and mortality. This is primarily attributed to the emergence of multiple drug-resistant (MDR) hypervirulent clones such as *S. aureus* USA300 strain, (hereafter referred to as SAUSA300) [1, 2]. SAUSA300 causes several types of infections ranging from simple skin infections to severe life-threatening infections, including pneumonia, meningitis, osteomyelitis, endocarditis, toxic shock syndrome, bacteremia, and sepsis [3]. The continuous emergence of MDR in MRSA makes its antibiotic treatment increasingly challenging [4, 5]. Accordingly, the SAUSA300 strain has shown variable levels of antibiotic resistance to the majority of the available antibiotics for MDR-MRSA infections. Despite this, few novel antibiotics have been developed in the past decade to address the growing concern of MDR-MRSA infections. Consequently, a clear gap exists between the discovery of novel antibiotics and the emergence of MDR pathogens [6]. Furthermore, as some antibacterial agents, currently in clinical trials, are derived from well-known antibiotic classes, their efficacy against MDR strains might be limited due to pre-existing intrinsic resistance and the emergence of newresistant clones [7, 8]. Therefore, controlling infections caused by hypervirulent MDR SAUSA300 requires the identification of novel antibacterial drugs with novel targets to avoid cross-resistance with conventional antibiotics [9].

In pursuit of novel target-based antibiotics, we investigated the antibacterial activities of G-quadruplex (G4/Gq) binding molecules, which have been minimally explored in bacteria [10]. G-quadruplexes (G4s), in general, are guanine-rich repeats in DNA/RNA present in variable (> 2–4) tracts at a certain interval of nucleotides (N). G4s can fold or stack into four-stranded secondary structures [11] and are abundantly present in all domains of life including viruses [12], bacteria [13], parasites [14–16], and humans [17]. G4s control the transcription, translation, genomic stability, and repair, thereby influencing various physiopathologies [17] including various cancer types. Therefore, the G4 structure and the binding ligands are important in the field of cancer research, owing to their presence in the regulatory regions of genes associated with cancer [18, 19]. Furthermore, intensive studies have been performed to understand the roles of G4s in viral life cycles, to develop novel antiviral drugs targeting viral G4s [20, 21]. However, the potential utilization of G4 as an antibacterial target for developing antibiotics remains largely unexplored [10, 13, 22–24]. Nevertheless, genome-wide bioinformatics analyses of G4 have been well-studied in bacteria such as *Escherichia coli* [25], *Mycobacterium tuberculosis* [26], *Streptococcus pneumoniae* [27], and *Klebsiella pneumoniae* [28]. Recently, the antibacterial activity of an azobenzene G4-binding compound against resistant *E. coli* has been reported [29]. Naphthalene diamide, a G4-binding compound, can inhibit both gram-negative and -positive bacteria [30]. Accordingly, we hypothesized that G4-binding ligands can potentially alter the expression of genes relevant to virulence and/or survival in the hypervirulent MDR SAUSA300 by binding to genomic G4s, which can be used as a strategy for their development as antibacterial drugs.

To prove this hypothesis, we assessed the antimicrobial efficacy of representative G4-binding ligands against SAUSA300. From 10 tested ligands, N-methyl mesoporphyrin IX (NMM) showed the most potent antibacterial activity against SAUSA300 by impacting the cell wall and cell division, as determined using confocal and scanning electron microscopy. Based on these findings, the division cell wall (*dcw*) cluster genes synchronously controlling both the cell division and cell wall biogenesis were analyzed. The findings revealed the presence of guanine-rich tracts creating several possibilities of G4 in the promoter region (P_*mraZ*_) of the highly conserved master regulator, *mraZ*. The potent antibacterial activity of NMM was primarily manifested by regulating the *dcw* gene cluster by stabilizing the G4 structure of P_*mraZ*_ and consequently impacting the transcriptional inhibition of key genes controlling cell division and cell wall biogenesis. This is an initial report on the antibacterial potential of G4-binding ligands against hypervirulent MDR SAUSA300. The research serves as an important milestone in the development of G4-binding compounds as antibiotics against drug-resistant pathogens*.*

## Materials and methods

### Chemicals and reagents

Most of the chemicals used in this study were of analytical grade and purchased from Sigma-Aldrich (St. Louis, MO, USA) unless otherwise indicated. The G4-ligands with their molecular structures screened for their antibacterial activity are shown in Supporting Information, Fig. S1. The G4 compounds were procured as BRACO19 trihydrochloride (GC50140, GLPBIO); Quarfloxin or CX-3543 (A12380, AdooQ), TMPyP2 or meso-Tetra (3-pyridyl) porphine (T40846, Frontier Scientific), TMPyP4 tosylate (GC12092, GLPBIO), PDS or Pyridostatin trifluoroacetate salt (18013, Cayman Chemical), PhenDC3 trifluoromethanesulfonate (CS-7711, Chemscene), Thioflavin T (2390-54-7, MedChemExpress), Quinacrine dihydrochloride (69-05-6, MedChemExpress), N-methyl mesoporphyrin IX or NMM (GC44416, GLPBIO), and Quercetin (117-39-5, MedChemExpress) (Fig. S1). The stock solution of NMM was prepared in DMSO and the working solution was diluted in water. Macrogen oligonucleotide purification Cartridge (MOPC) purified oligonucleotides for gene expression and cloning, or HPLC grade oligonucleotides for G4 biophysical studies were purchased from Macrogen (Korea).

### Bacterial cell and growth conditions

All the bacterial strains used in this study are shown in Supplementary Table S1. SAUSA300 and *E. coli* cells were cultivated for 16–18 h at 37 °C on tryptic soy agar (TSA) (Sigma-Aldrich, USA) and Luria Bertani agar (LBA) (Becton, Dickinson, USA) plates containing 1.5% micro-agar, respectively. Bacterial broth cultures were prepared by inoculating a single colony in tryptic soy broth (TSB) or Luria Bertani (LB) media for 14–16 h under orbital shaking conditions at 200 rpm at 37 °C. Bacterial growth was measured using a Gen5 BioTek microplate reader (BioTek, USA) to measure optical density at 600 nm (OD_600 nm_) and colony-forming units (CFU) per mL [(OD_600 nm_ = 1.0 = 1.0 × 10^9^ CFU/mL for SAUSA300), and (OD_600 nm_ = 1.0 = 1.0 × 10^8^ CFU/mL for *E.coli*)]. In each experiment, bacterial cells were harvested by centrifugation at 4000 rpm (2701 × *g*) at 4 °C and washed with Phosphate-buffered saline (PBS, pH 7.2). All wild-type and recombinant strains used in this study are listed in Supporting Information Table S1.

### Antimicrobial susceptibility assay

The minimal inhibitory concentration (MIC) assay was performed using the broth microdilution protocol approved by the Clinical and Laboratory Standards Institute (CLSI) [31]. G4-binding ligands were diluted at a range of 10–0.0625 µM in Muller Hinton broth (MHB) using a two-fold serial dilution in 96-well plates, with each dilution having a volume of 50 µL. The final volume of 100 µL in each well was achieved by adding 50 µL of a bacterial dilution containing 5 × 10^5^ CFU/mL bacteria. For the control growth of SAUSA300, the G4-binding ligand solution was substituted with MHB. To ensure sterility, 100 µL of MHB without bacteria was used. The plates were incubated for 18 h at 37 °C under static culture conditions. The absence of turbidity indicated growth inhibition, while the spectrophotometrically measured turbidity (OD_600 nm_) indicated bacterial growth. The MIC value is the lowest antibiotic concentration at which visual turbidity is suppressed.

### Killing kinetics of NMM

The time-killing assay was performed as described earlier [32] to evaluate the antibiotic activity of NMM. Strong antibiotics, vancomycin, and tetracycline (Van and Tet) were used against SAUSA300 as comparators. Bacteria were cultured overnight in TSB at 37 °C. Subsequently, a fresh batch of TSB was inoculated with the overnight bacterial culture to facilitate growth until it reached the mid-log phase. Bacterial cells were introduced into 14 mL polystyrene round-bottom tubes (Falcon, Bedford, USA) with an initial inoculum of 1 × 10^9^ CFU/mL in 1 × PBS. NMM, Van, and Tet were supplemented at concentrations equivalent to 1.0, or 10 times their experimentally determined MICs of 5, 0.6, and 2.5 µM, respectively [33]. Bacteria grown in the presence of the same percentage of antibiotic-diluent DMSO served as the dilutant’s growth control. The cultures were subjected to incubation in a shaking incubator at 37 °C. At specific time intervals of 0, 4, 8, and 12 h samples were taken from each tube. These samples were then diluted serially and subsequently enumerated for CFU count on blood agar plates. To assess the efficacy of antibiotic killing, a widely recognized lysis agent, Triton X-100 (0.05%) was used as a positive control.

### Live/dead staining of bacterial cells

SAUSA300 was cultured until the bacteria reached the log growth phase, and OD_600 nm_ was maintained at 1. The cells were treated with 1 × (5 μM) and 10 × NMM concentrations for 2 h at 37 °C. After washing, the cells were treated with the Live/Dead Viability Kit (Cat #L7007; Invitrogen, Carlsbad, USA). SYTO9 (λex = 485 nm and λem = 503 nm) equally labeled all the cells with a green-fluorescent color; however, PI (λex = 535 nm and λem = 610) specifically labeled only the dead cells with a red-fluorescent color. It is noteworthy that the PI red fluorescence emission intensity of dead cells was significantly higher than that of NMM when excited at 535 nm (PI λex = 535 nm). PI is typically excited at 535 nm and red fluorescence can be detected in a 610/20 bandpass, while NMM was excited at 399 nm (λex = 399 nm) and the red fluorescence emission was detected at peaks at 610–614 nm. The stained cells were rinsed twice with PBS and then examined using confocal laser scanning microscopy (CLSM) to quantify NMM-induced cell death.

### Evaluation of NMM internalization to SAUSA300

Bacterial strains were cultured in TSB or LB media until they reached the logarithmic phase. A 1:100 dilution of bacteria was cultured for 2 h in the presence of 1.0 × NMM. The collected bacterial cells were rinsed with PBS, and OD_600 nm_ was adjusted to a value of 0.5, corresponding to approximately 5.0 × 10^8^ CFU/mL. The cells were stained with wheat germ agglutinin Alexa Fluor 488 conjugate stain (Invitrogen, USA), as described earlier [34]. The remaining unbound dye was removed by washing with 1 × PBS. Bacterial slides were made by placing 10 μL of the stained culture onto glass slides. These slides were then used for confocal imaging using the Laser Scanning Microscopy (LSM 710 Meta, Carl Zeiss, Germany).

### Scanning electron microscopy (SEM)

The SEM analysis of SAUSA300 was conducted without or with NMM as mentioned in an earlier study [35]. Bacteria were treated with 1 × and 10 × NMM solutions for 12 h at 37 °C. Negative controls included solutions containing 0.5% and 5% DMSO. Triton X-100 (0.05%) treated cells were used as a positive control. The cells were fixed by incubating them in a 2% glutaraldehyde solution (Sigma Grade I, 25% in H_2_O,) overnight at room temperature (RT, 25 ± 2 °C). Following this, the cells were washed twice with PBS and dehydrated in an ethanol gradient of 10, 20, 40, 60, 80, and finally 100% ethanol. Cells were placed on silicon wafers and dried at room temperature before being subjected to SEM. After drying, platinum sputtering was performed to visualize the samples using SEM at different magnifications.

### Quantitative reverse-transcription polymerase chain reaction (qRT-PCR) analysis

Bacteria were grown in TSB at 37 °C without or with NMM until they reached the mid-log phase. Total RNA was isolated using the Qiagen RNeasy kit (Hilden, Germany) according to the manufacturer’s protocol. Total RNA (1 µg) was treated with RNase-free DNase I (1 U) (amplification grade; Sigma-Aldrich, USA) at room temperature for 15 min. The DNase I reaction was inhibited using a stop-buffer followed by the inactivation of DNase at 70 °C for 10 min. A random hexamer premix (RNA-to-cDNA EcoDryTM premix; Takara Bio, Japan) was used to prepare cDNA from the isolated DNA-free mRNA. qRT-PCR was performed with an annealing temperature of 55 °C and SYBR Green Supermix (Bio-Rad, USA). The *gyrA* gene was used as a housekeeping gene for SAUSA300. The primers used in this study are listed in Supplementary Table S2. Expressions of the tested genes were normalized using the 2^−ΔΔCT^ method, to calculate the relative gene expressions [36]. Three independent qRT-PCR experiments were performed, and statistical significance was calculated using Student’s *t*-test.

### Circular dichroism (CD) spectral analysis

To assess the CD spectra, 15 µM wild-type (WT) or mutant (Δ) G4 DNA samples were prepared in a standard G4 buffer consisting of 10 mM Tris–HCl (pH 7.5) and 100 mM KCl. The G4 samples underwent annealing in a thermal block (Eppendorf, Germany), with denaturation at 95 °C for 10 min followed by gradual cooling with ramp rate of 0.5 ºC/min until 4 ºC. The G4 samples were stored at 4 °C overnight prior to use in the experiment. For CD spectra measurement, the G4 samples (15 µM) were mixed at 1:2 [G4 oligonucleotide]: [ligand] ratio in G4 buffer and incubated at room temperature for 30 min. Measurements were performed using a 1 mm quartz cuvette in a sample volume of 200 µL. The CD spectra were obtained at 25 °C, at wavelengths ranging from 220 to 320 nm. The scan speed was set at 100 nm/min, with a data pitch of 0.1 nm and a bandwidth of 1 nm. All CD tests were performed using a CD spectropolarimeter (JASCO J-810, Japan) with a CDC-426F Peltier temperature controller.

### CD thermal melting (Tm) analysis

For CD Tm analysis, the G4 DNA samples (15 µM) were mixed without or with NMM at 1:2 [oligonucleotide]: [ligand] ratio in G4 buffer and incubated at room temperature for 30 min as mentioned previously in CD spectral analysis. For CD Tm analysis the samples were heated from 25 to 90 °C at a ramp rate of 1 °C per min, with a data pitch of 0.1 °C. The Tm value was determined at the wavelength exhibiting the maximum degree of ellipticity. Data were obtained in two trials, and a sigmoidal four-parameter equation was used to fit the curves using GraphPad Prism 8 software. Tm was calculated when the G4s were present in equal proportion in the folded and unfolded states. Error bars represent ± standard error of the mean.

### Electrophoretic mobility shift assay (EMSA) of G4 DNA

EMSA was performed using non-denaturing polyacrylamide gel electrophoresis (PAGE) to evaluate the mobility shifts of various G4 DNA as described earlier [37]. Briefly, 15% native polyacrylamide gel was prepared in 1 × Tris Borate EDTA (TBE) buffer. The gel was pre-run in 1 × TBE buffer containing 100 mM KCl at 70 V for an hour at 4 ºC. Then, the G4 samples of WT P_*mraZ*__G4_3 and its 4 individual point mutants namely P_*mraZ*__∆G4_3a, P_*mraZ*__∆G4_3b, P_*mraZ*__∆G4_3c and P_*mraZ*__∆G4_3d G4 DNA were loaded on native PAGE gel by mixing 5 μL of G4 samples (2 µM) with 1 μL of 6 × Orange DNA Loading Dye. Ultra-Low DNA Ladder GeneRuler (ThermoFisher Scientific, USA) was used for verifying differential migration of various G4 samples. The gel was run in 1 × TBE with 100 mM KCl at 70 V for 2 h in cold room (4 ºC) followed by staining with 1 × SYBR-Gold nucleic acid stain at room temperature (ThermoFisher Scientific, USA).

### Simulation study of G4

The three-dimensional G4 structures of WT P_*mraZ*__G4_3 and its G4-destabilized mutant P_*mraZ*__∆G4_3d were prepared using 3D-NUS [38] with all parallel strands, based on CD data showing the characteristic curve for intramolecular parallel G4. The mutant G4 was prepared by mutating the respective bases using PyMOL (v2.5) [39]. The WT P_*mraZ*__G4_3 and mutant P_*mraZ*__∆G4_3d were simulated without or with NMM. The K^+^ ion was placed at the center of the G-tracts to stabilize the G4 structure. The structure of G4 drug NMM was obtained from PubChem (CID 656404) [40] in SDF format and converted to PDB format using Avogadro molecular editor (v1.2) [41]. DNA was parameterized using AMBER ff99bsc0 force field with CUFIX corrections for non-bonding interactions [42, 43] and NMM was parameterized using the antechamber module with GAFF [44, 45]. The molecular dynamics (MD) simulations were done using Gromacs (v2023.2) [46] with CUFIX-corrected AMBER ff99bsc0 force field under periodic boundary conditions. The complex was solvated in a cubic box with a fixed distance of 1 nm between the solute and the box using the TIP3P water model [47]. Sufficient KCl salt was added to neutralize the system and to maintain the K^+^ ion concentration up to 100 mM. In the case of simulations with G4 ligand, one molecule of NMM was randomly inserted into the system. Energy minimization was carried out in two steps, twice with restraint (1.0 kcal mol^−1^ Å^−1^) for 10 ps each using the steepest descent and conjugate gradient methods, respectively; followed by an unrestrained minimization of 250 ps. Long-range interactions were calculated using Particle Mesh Ewald (PME) [48] with Fourier grid spacing of 0.16 nm and the cutoff distance for short-range Vander Waal interactions was set to 1.2 nm. The SHAKE algorithm [49] was used to stabilize the motion of hydrogens; and LINCS algorithm [50] was used to put constraints on hydrogen bonds. The system was maintained at a constant temperature and pressure (310 K and 1 atm respectively) allowing an integration time step of 2 fs. The final production MD was carried out for 100 ns. For trajectory analysis, binding energy was calculated for the last 20 ns of the WT P_*mraZ*__G4_3 and mutant P_*mraZ*__∆G4_3d G4s with ligands using gmx_MMPBSA [51]. The structures were finally visualized in PyMOL, and the intramolecular distances in the G4 tetrad were computed.

### UV–visible titration and thermal difference spectra (TDS) analysis

UV–visible titration assay was performed to understand NMM interaction with WT P_*mraZ*__G4_3 and mutant P_*mraZ*__∆G4_3d in solution as described earlier [21]. TDS analysis of WT P_*mraZ*__G4_3, and mutant P_*mraZ*__∆G4_3d with abolished G4 structure were carried out using a JASCO UV–VIS spectrophotometer (Jasco-V750, Japan) equipped with a Peltier temperature controller (LAUDA, Germany). Melting curves of WT P_*mraZ*__G4_3, and mutant P_*mraZ*__∆G4_3d were collected at 20 °C and 90 °C with a heating ramp rate of 1 °C/min. The TDS spectra was generated by subtracting G4 buffer corrected spectra of 20 °C from those at 90 °C. The spectra were normalized using the maximum TDS value to get the normalized TDS.

### One-dimensional H^1^ nuclear magnetic resonance (NMR) spectroscopy

Proton NMR experiments were performed using 700 MHz BRUKER NMR spectrometer (Bruker, Germany) as described earlier [10, 52]. Briefly, the G4 samples were prepared as described in the previous section of CD spectral analysis. The 1D H^1^ NMR spectra were recorded using 3 mM G4 in 10% D_2_O solvent at room temperature (25 °C) with 20 ppm spectral width. Both the WT P_*mraZ*__G4_3 and mutant P_*mraZ*__∆G4_3d were resuspended in 10 mM Tris–HCl without or with 100 mM KCl (pH, 7.5). NMR data were processed and analyzed by SpinWorks v4.0. software.

### Steady-state fluorescence titration study

Steady-state fluorescence titration assay was performed using Spark 10M Multimode Microplate Reader (Tecan, Switzerland) in 96 wells black/flat bottom microtiter plates at 25 °C as described earlier [53]. Briefly, increasing concentrations of drugs (0–600 μM) were mixed with WT P_*mraZ*__G4_3 (0.5 μM) to a final volume of 100 μL in G4 buffer [10 mM Tris–HCl and 100 mM KCl (pH 7.5)], and incubated for 30 min in a rotary shaker. The excitation and emission wavelength of ligands are indicated in the graphs. The change in emission fluorescence (ΔF) for all datasets was normalized to the wells containing only DNA without G4-ligands. The equilibrium dissociation constant (*K*_d_) was determined by non-linear curve fitting wherein two-site saturation model was used to extrapolate the curve between ΔF and DNA concentration (Y = [(B_max_Hi × X) /(*K*_d_Hi + X)] + [(B_max_Lo × X) / (*K*_d_Lo + X)] where B_max_ is maximum binding) in GraphPad Prism 8.

### G-quadruplex fluorescence intercalator displacement (FID) assay

The FID assay was performed in a buffer containing 10 mM sodium cacodylate and 100 mM KCl (pH 7.4). To determine the binding affinity (*K*_d_) of thiazole orange (TO) to WT P_*mraZ*__G4_3, 1 μM G4 DNA was mixed with increasing concentrations TO (0 to 5 μM) in 100 μL final volume. The G4 DNA-TO complex was incubated using an orbital shaker for 5 min at 25 ± 1 °C. The fluorescence spectra were measured using a Synergy™ NEO HTS Multi-Mode Microplate Reader (Agilent BioTek, USA). The emission spectra were recorded from 520 to 650 nm when excited at 501 nm. The area under the curve (AUC_0_) for the spectra was calculated and the association curve was plotted by non-linear curve fitting.

To determine the competitive displacement of TO by NMM, WT P_*mraZ*__G4_3 (1 μM) was mixed with TO (2 μM) and incubated for 5 min in a rotatory shaker, followed by the addition of increasing concentrations of NMM (0 to 20 μM). After 15 min of shaking, the fluorescence spectra were recorded. The degree of TO displacement was calculated from the AUC_0_ for the spectra as:

$$\%\text{TO displacement}=100-{(\text{AUC}}_{\text{NMM}}/{\text{AUC}}_{0})\times 100)$$

where AUC_NMM_ is the area under the curve in the presence of NMM concentration.

### Reporter assay by creating a promoter-probe vector

Due to the extreme susceptibility of NMM against SAUSA300, the reporter strains were constructed in *E. coli* DH5α to assess the gene regulation under P_*mraZ*_ promoter harboring G4 structure. In this context, ten representative strains were taken for *E. coli, Enterococcus faecium*, *Klebsiella pneumoniae*, *Pseudomonas aeruginosa*, *Enterobacter sp.* and *Staphylococcus aureus* including the SAUSA300 strain (Table S3). The conservation analysis of P_*mraZ*_ promoter-specific G4 in *dcw* cluster was analyzed by extracting the P_*mraZ*_ promoter from the genomic sequences of both gram-positive and gram-negative bacterial strains. The DNA sequences, -200 bp from the transcription start site (TSS) of *mraZ* gene sequences were extracted for each strain from NCBI database, which were then aligned using Clustal Omega v2.1 [54]. The sequence logo for the bases at each position in the region of interest was created with the WebLogo 3 web application [55]. All the DNA fragments were cloned by following the standard recombinant DNA technology protocols as described in Molecular Cloning: A Laboratory Manual by Sambrook [56]. The promoter-probe vector was constructed with multiple cloning sites and *mCherry* gene by taking the backbone of *E. coli*-*S. aureus* vector pRP1195 [57]. The pRP1195 vector possesses the *luxBADCE* gene cloned under P_*gapA*_ promoter at two *EagI* sites, pBR322 origin of replication for *E. coli,* a temperature sensitive origin of replication (*pE194 ts ori*) for *S. aureus*, with ampicillin and chloramphenicol resistance genes for *E. coli* and *S. aureus*, respectively. Unfortunately, the pRP1195 vector does not possess any choice to replace the P_*gap A*_ promoter due to lack of multiple cloning sites (MCS). Therefore, a new promoter-probe vector with MCS and *mCherry* reporter gene was constructed. Briefly, the P_*gapA*_ tagged with *luxBADCE* genes was deleted using *EagI* restriction endonuclease digestion followed by self-ligation which generated the vector backbone (Table S1). This vector backbone was used for re-cloning of the synthesized DNA fragment with MCS harboring P_*gapA*_ promoter (underlined) (GCG GCC GCC TTA AGG GGC CCG CTA GCC GCC GGC GGT TTA AAC GCG CGC *TTG ACA CTG CGT AAG GTT TGT GTT ATA AT*A CTA GTT ACG TAC TCG AGG GAT CC) at *EagI* and *BamHI* which is designated as *pACKK*-P_*gapA*_-MCS. The *mCherry* gene with *lamda* t0-terminator was amplified from ‘*pQ30-mCherry-lamda t0 terminator’* using the primers pair *mCherry*_*XhoI*_*Fwd* and *mCherry_BamHI_Rev* (Table S2) cloned at the same sites to create *pACKK*-P_*gapA*_-*mCherry* promoter-probe vector (*pACKK*-P_*probe*_). The promoter-less-*mCherry* empty vector (EV), *pACKK*-*mCherry* was created by deleting the P_*gapA*_ using *PmeI*/*SnaBI *restriction sites followed by self-ligation. The P_*mraZ*_ promoter was PCR amplified from SAUSA300 genomic DNA with vector homology using the primers pair *Gib_P*_*mraZ*_*_Fwd* and *Gib_P*_*mraZ_*_*Rev* (Table S2) and assembled between *PmeI/SnaBI* in *pACKK*-P_*gapA*_-*mCherry* promoter-probe vector by replacing the P_*gapA*_ promoter to obtain *pACKK*-P_*mraZ*_-*mCherry* construct. The site directed mutagenesis (SDM) in P_*mraZ*_ promoter’s G4-motif (WT P_*mraZ*__G4_3) was performed using the quick-change primers pairs *Mut_P*_*mraZ*_*_Fwd* and *Mut_P*_*mraZ*_*_Rev* (Table S2) to attain a mutant P_*mraZ*_ promoter, P_*mraZ-*mut_ with destabilized G4-motif (P_*mraZ*__ΔG4_3d) and the resultant plasmid is designated as *pACKK*-P_*mraZ*-mut_-*mCherry* (Table S1). All the constructs were verified by DNA sequencing. The details of these clones are shown in Table S1.

### In vitro transcription/translation (IVT) assay

To assess the impact of G4-motif of P_*mraZ*_ promoter on bacterial coupled transcription/translation, three clones were used namely promoter-less *mCherry* empty vector (EV), the fusion protein MraZ-_His6_-mCherry expression under P_*gapA*_ and P_*mraZ*_ promoters. The promoter-less EV and P_*gapA*_ promoter served as negative and positive controls for the expression of fusion protein, respectively. Briefly, to create the *pACKK*-P_*gapA*_*-mraZ-*_*his6*_-*mCherry* vector*,* the *mraZ *open reading frame (*orf*) with C-terminal his6-tag without stop codon *(mraZ-*_*his6*_*)* was PCR amplified using primers pair *mraZ-orf-Fuse-Fwd* and *mraZ-NS-his6_Rev* (Table S2) flanked with *SpeI* and *XhoI* restriction enzymes. The *pACKK*-P_*gapA*_-*mCherry* vector was restriction digested with *SpeI and XhoI* sites. The *SpeI*-*XhoI* digested PCR product, *mraZ-*_*his6*_ without stop codon was cloned in-frame with *mCherry* gene at same site of *pACKK*-P_*gapA*_-*mCherry* which expressed the fusion protein MraZ_-His6_-mCherry under* P*_*gapA*_ promoter*.* Next, the P_*mraZ*_ promoter with *mraZ* gene without stop codon and with C-terminal his6-tag (P_*mraZ*_-*mraZ*-_*his6*_) was PCR amplified with *NheI* and *XhoI* using the primers pair *P*_*mraZ*_*-Fuse_Fwd* and *mraZ-NS-his6_Rev* (Table S2). The *pACKK*-P_*gapA*_-*mCherry* vector was restriction digested with *NheI and XhoI* sites which deleted the P_*gapA*_ promoter. The PCR product flanked with *NheI* and *XhoI* was restriction digested and cloned in-frame with *mCherry* gene at the same sites of *pACKK*-P_*gapA*_-*mCherry* vector to yield *pACKK*-P_*mraZ*_-*mraZ*-_*his6*_-*mCherry* construct to express recombinant MraZ-_His6_-mCherry protein under P_*mraz*_ promoter (Table S1)*.*

To further validate the protein expression data and the role of WT P_*mraZ*__G4_3 G4-motif, in vitro coupled transcription/translation (IVT) templates were amplified with T7 promoter plus G4-motif (WT P_*mraZ*__G4_3) [568 bp, *T7* + *GQ_P*_*mraZ*_*_Fwd* & *mraZ-*his_*Stop-Rev* (Table S2)] and minus G4-motif [(498 bp, *T7-GQ_P*_*mraZ*_*_Fwd* & *mraZ-his_Stop-Rev* (Table S2)]. The in vitro coupled transcription/translation (IVT) assay was performed by using TnT Quick coupled transcription/translation system (Promega, USA) as described by the manufacturer’s instruction in 50 µL reaction with varying concentrations of NMM (10, 20, and 40 µM) wherein the control was treated with the equivalent volume of DMSO carrier.

### Western blot and immunodetection

The recombinant protein MraZ-His_6_ of the IVT reactions was detected through western blotting and immunodetection as described earlier [21]. Briefly, proteins were electrophoretically resolved using 16% SDS-PAGE and electroblotted onto PVDF membrane (Immobilon, Merck Millipore, USA). Immunodetection of recombinant MraZ-His_6_ protein of IVT reactions without or with NMM was carried out with a primary mouse-monoclonal anti-His antibody (Santa Cruz Biotechnology, USA). MraZ-His_6_ proteins from various reactions were visualized by using a secondary antibody (Goat anti-mouse IgG-HRP Horseradish peroxidase conjugate; Santa Cruz Biotechnology, USA). The immuno-cross-reacting protein was detected using a highly enhanced peroxidase detection kit pico EPD (ELPIS BIOTECH, Korea) by peroxidase-catalyzed luminol chemiluminescent oxidation reaction.

### Assessment of cell viability

RAW264.7 cells were cultured in high-glucose Dulbecco’s Modified Eagle’s Medium (DMEM), supplemented with 10% fetal bovine serum (FBS) and 1% penicillin/streptomycin. The cells were maintained at 37 °C in a CO_2_ incubator. The relative cell viability was quantitatively assessed using a cell counting kit Quanti-Max WST-8 (BIOMAX, Korea). Concisely, approximately 5000 cells were added to each well of a 96-well plate with 200 μL DMEM. The plate was incubated at 37 °C in a 5% CO_2_ incubator for 6 h, 24 h, and 48 h either with or without the drug. Subsequently, a 3% solution of WST-8 reagent was added to each well, followed by incubation for 2 h at 37 °C. Following this, the absorbance was quantified at 450 nm using a microplate reader. The relative percentage cell viability was determined using the following equation:

$$\text{Relative \% cell viability}=\frac{\begin{array}{c} \\ Absorbance \, treated \, sample\end{array}}{\begin{array}{c} \\ Absorbance \, untreated \, sample\end{array}}\times 100$$

The live/dead staining method was used to visualize both viable and non-viable cells. Approximately 20,000 cells were initially exposed to different doses of drugs in 24-well plates containing a final volume of 500 μL DMEM for 24 h. Subsequently, a staining solution comprising 10 μL 6.7 μM acridine orange (AO, λex = 490 nm, and λem = 520 nm) (Life Technology, USA) and 750 μM PI was added to the cells. The mixture was thoroughly mixed and allowed to incubate for 10 min at room temperature. Cell images were captured using a fluorescence microscope (Nikon Eclipse Ti, Nikon Instruments Inc., USA).

### Evaluation of SAUSA300 infection and NMM treatment

#### In vitro infection of RAW264.7 cells

RAW264.7 cells were cultured in six-well plates by adding 5 × 10^5^ cells to 2 mL DMEM and incubated for 24 h. The medium was replaced with 1 mL of fresh DMEM medium without FBS 2 h before bacterial infection. In the infection experiment, bacterial cells in the logarithmic phase were rinsed with ice-cold PBS and resuspended in PBS. The multiplicity of infection (MOI) was set at 10. Both host and pathogen cells were chilled on ice for 15 min to achieve synchronization. Following this, bacterial cells were mixed with mammalian cells at low temperatures (4 °C) on ice. The infection process began by transferring the host–pathogen mixture to an incubator set at 37 °C and 5% CO_2_. Following 30 min of infection of SAUSA300 or *E. coli* CFT073 [58, 59], the combination of the host and pathogen cells was washed three times with PBS, to eliminate non-adherent host cells and any surplus extracellular bacteria. To determine the efficacy of NMM, residual extracellular bacteria and those attached to the host cell surface were treated with NMM at 0.5 × and 1 × MIC for 2 h at 37 °C. The host–pathogen complex was washed three times with PBS at 4 °C and centrifuged at 500 × *g* for 5 min, to eliminate any residual extracellular bacteria and antibacterial substances. The number of bacteria that were taken up by the host cells was determined by counting the CFU in the host cell lysates. The lysates were generated by treating the harvested host cells with 1 mL of a solution containing 0.02% Triton X-100 in water. The lysates were then diluted in PBS, plated, and incubated at 37 °C for 18 h. For the live and dead assay, following drug treatment, both live and dead cells were rinsed with PBS and stained using the live/Dead Viability Kit. The labeled cells were washed twice with PBS and subsequently analyzed using confocal microscopy, to determine the extent of infection.

### *Galleria mellonella* infection model

SAUSA300 and *E. coli* CFT073 cells were cultured overnight in TSB and LB media, respectively at 37 °C under orbital shaking culture conditions at 200 rpm. The overnight grown cultures were reinoculated at 100-fold dilution in their corresponding fresh media for 6 h. Bacterial cells were recovered by centrifugation (2701 × *g*, 4 °C) and washed once with 1 × PBS. The cell numbers were maintained by adjusting OD_600 nm_ at 1 in 1 × PBS. *G. mellonella* was used within one week of delivery. Each control or treatment group consisted of ten waxworms (n = 10). These waxworms were injected with 2.0 × 10^5^ (SAUSA300) or 1.0 × 10^5^ (*E. coli* CFT073) cells in a final volume of 20 µL into left posterior leg using a 0.3 mL syringe (Becton, Dickinson and Company, USA) [60]. The NMM drug (20 μL) was administered after 1 h of infection *via* the right posterior proleg [61] and incubated at 37 °C for observation. Two drug concentrations (*i.e.* 2.5 μM and 5 μM) were selected for the in vivo validation of NMM antibacterial activity. In each experiment, a control group of worms was injected with 20 µL 1 × PBS. The survival of larvae was monitored for 4 days post-infection to evaluate the antibacterial efficacy of NMM under in vivo conditions.

### Adaptive laboratory evolution

To compare NMM as an antimicrobial resistance evolution or adaptation-proof potent antibacterial agent, adaptive laboratory evolution (ALE) of SAUSA300 was conducted for 8 days using 0.5 × MIC of vancomycin (Van) and NMM along with control SAUSA300 without any treatment as described earlier [62]. The cell growth was monitored by measuring the optical density at 24 h intervals. The spent media was replenished with fresh TSB media supplemented with vancomycin or NMM at 0.5 × MIC or TSB media only for no treatment control. The adaptive evolution was assessed by challenging the adapted cells at 1 × MIC of antibacterial agents. The adapted (Evolved Van SAUSA300 and Evolved NMM SAUSA300), and control SAUSA300 cells which has never been exposed to any antibacterial agent were repeatedly sub-cultured by inoculating 1:1000 inoculum followed by measuring growth for 16–18 h at 37 °C under aerated culture conditions.

## Results

### Screening of G4-binding ligands for antibacterial activity against SAUSA300

We screened the antibacterial activity of 10 representative G4-binding ligands (Quinacrine, PhenDC3, PDS, Quercetin, Quarfloxin, TMPyP4, Thioflavin T, TMPyP2, BRACO19, and NMM) (Fig. S1) against SAUSA300 in 96 microtiter plates without aeration in small volume of 100 µL as a preliminary screen to evaluate if these G4 ligands possess any antibacterial activity against MDR SAUSA300. Unexpectedly, under these conditions, all the tested ligands showed variable levels of antibacterial activity at a concentration of 10 μM (Table S4). Among them, NMM apparently showed the most effective ligand, exhibiting maximum growth inhibition with a minimum inhibitory concentration (MIC) of 5 μM (2.9 µg/mL) (Table S4). However, these preliminary observations (Table S4) further verified using growth inhibition measurement under optimal bacterial culture conditions, IC_50_ determination followed by drug internalization in SAUSA300 to choose an efficient antibacterial agent.

### Antibacterial activity confirmation of the G4-binding ligands

**Fig. 1:**
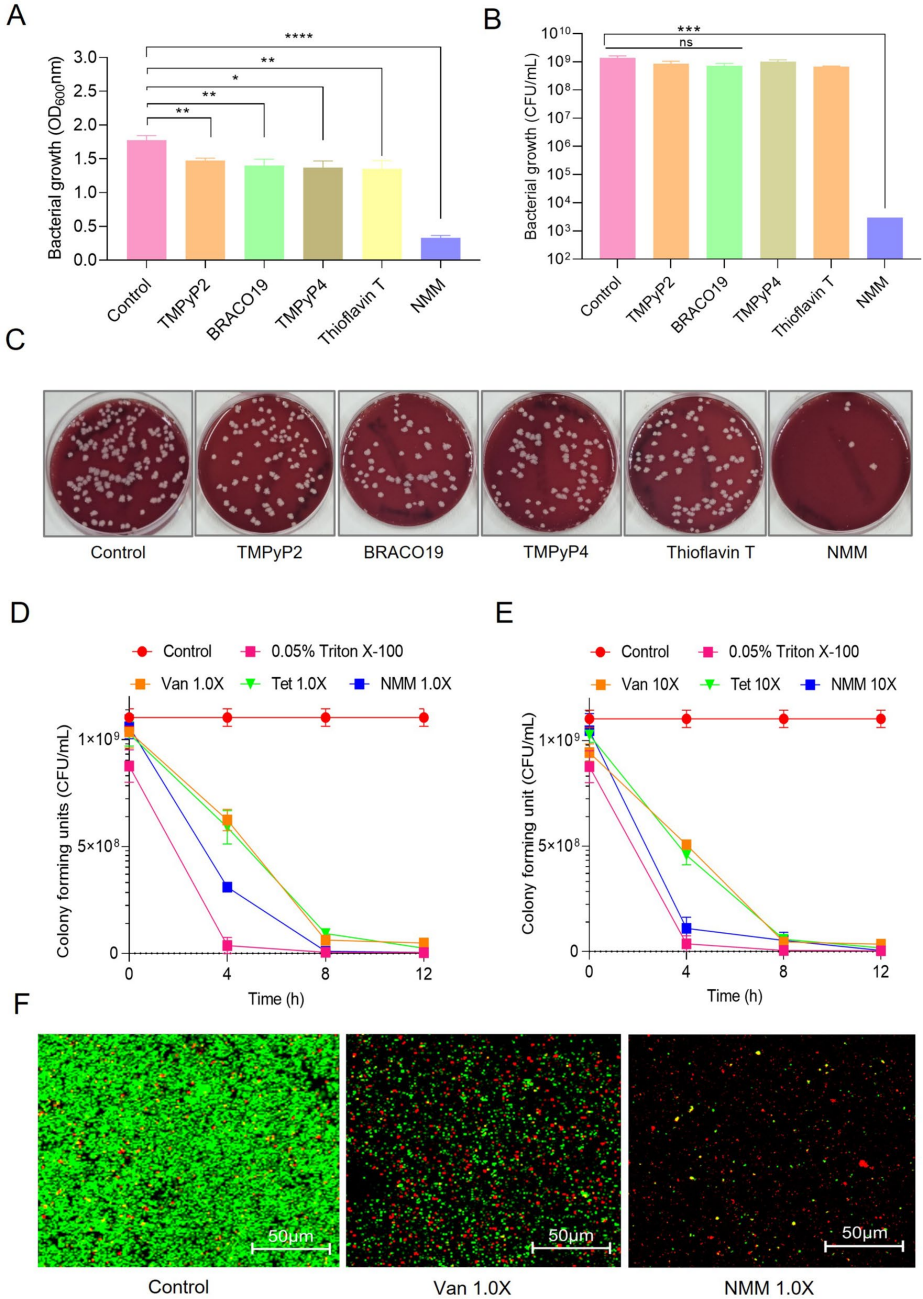
Confirmation of antibacterial activity. **A** The antibacterial activities of NMM, TMPyP2, BRACO19, TMPyP4, and Thioflavin T against SAUSA300 were examined by measuring cell growth in terms of OD at 600 nm. **B, C** SAUSA300 cell growth assessed as CFU/mL (**B**); representative sheep-blood agar plates showing the appearance of colonies during CFU enumeration (**C**). **D**, **E** Comparative killing kinetics of vancomycin (Van), Tetracycline (Tet), and NMM against SAUSA300 based on CFU/mL at 1.0 × (**D**); and 10 × (**E**) MIC of Van, Tet, NMM, and 0.05% Triton X-100 at different time points (0 to 12 h). **F** Comparative live/dead assay of SAUSA300 with 1 × MIC of NMM (5 µM) and Van (0.6 µM) using confocal microscopy, showing the proportion of live/dead SAUSA300 cells. SYTO9 and PI were used to stain the number of total and dead cells as green-fluorescent and red-fluorescent cells, respectively. All experiments were performed in triplicate and the average data was plotted with standard deviation. Significance of the data was analyzed using Student’s *t*-test. *p*-values less than 0.05 were considered significant (ns = non-significant *p* > 0.05, *p < 0.05, **p < 0.01, ***p < 0.005, and ****p < 0.0001)

To further confirm the antibacterial activity of the top five ligands exhibiting the higher levels of growth inhibition of SAUSA300 (Table S4), optical density (OD) at 600 nm and colony forming units (CFU)/mL of SAUSA300 were measured after treatment with 5 µM of each ligand under optimal aerated bacterial growth conditions. Briefly, 5 × 10^5^ bacteria/mL were cultured in media supplemented with 5 µM of each drug for 14–16 h, resulting in clinically irrelevant marginal bacterial growth inhibition for four ligands (TMPyP2, BRACO19, TMPyP4, and Thioflavin T) except NMM (Fig. 1A). Further determination of the CFU/mL count revealed non-significant reduction using BRACO19, TMPyP2, Thioflavin T, and TMPyP4. Consistent with the primary screening data, efficient CFU inhibition was observed using 5 µM NMM (Fig. 1B, C). The antibacterial activity of G4-binding ligands was further confirmed by 50% inhibitory concentration (IC_50_) and a drug internalization assay (Fig. S2 and S3). Consistent with the MIC data, NMM showed the most potent antibacterial activity and its IC_50_ was found to be 2.3 µM (Fig. S2J) which was lower than the IC_50_ of Quarfloxin, a previously identified antibacterial as the first G4-binding ligands (Fig. S2K). Furthermore, NMM showed the highest internalization (intracellular drug) to SAUSA300 cells (Fig. S3 H–H’) as compared to other G4 binding ligands when exposed to 5 µM of each drug for 2 h in TSB media (Fig. S3). Together these data demonstrate that NMM is an efficiently bioavailable G4 ligand to SAUSA300 cells, and thus, exhibited the most potent antibacterial activity among the tested G4-ligands against MDR hypervirulent SAUSA300 (Table S4, Fig. 1A–C, Fig. S2J-K, and S3 H–H’).

To assess the antibacterial efficacy of NMM compared to those of well-established antibiotics against SAUSA300, we conducted a killing kinetics evaluation using the CFU/mL assay. The killing kinetics of NMM against SAUSA300 were compared to those of vancomycin (Van) and tetracycline (Tet) at concentrations ranging from 1 to 10 × of their corresponding reported MIC values (0.6 and 2.5 µM for Van and Tet, respectively) [33]. At each time point, the CFU/mL counts were measured (Fig. 1D and E); 0.05% Triton X-100 was used as a positive control of cell lysis. These results revealed a significant decrease in CFU/mL in the positive control groups. Similarly, CFU/mL counts drastically reduced after 4 h of NMM treatment at both 1 × and 10 × MIC, whereas Van- and Tet-treated SAUSA300 displayed slower killing kinetics than that of NMM (Fig. 1D and E). Additionally, a comparative live-dead assay using confocal laser scanning microscopy** (**CLSM) was performed to further verify the antibacterial effect of NMM on SAUSA300. Untreated and 1 × MIC of Van-treated SAUSA300 were used as negative and positive antibiotic controls, respectively (Fig. 1F). The untreated control group showed predominantly live cells with a negligible number of red-fluorescent dead cells, while cells treated with 1 × MIC Van exhibited a relatively higher number of red fluorescent dead cells. Interestingly, treatment with 1 × MIC NMM resulted in cytolysis of SAUSA300 cells, and thus, a higher number of dead/lysed cells were observed than those with Van treatment (Fig. 1F). In summary, these results, along with the decrease in OD, CFU/mL counts, IC_50_, efficient bioavailability, killing kinetics, and live-dead assay, provide compelling evidence that NMM possesses potent antibacterial properties against SAUSA300, surpassing the killing potential of Van which is a well-established last-resort antibiotic for MDR *S. aureus* strains.

### Assessment of NMM binding or internalization and its impact on SAUSA300 cells

**Fig. 2:**
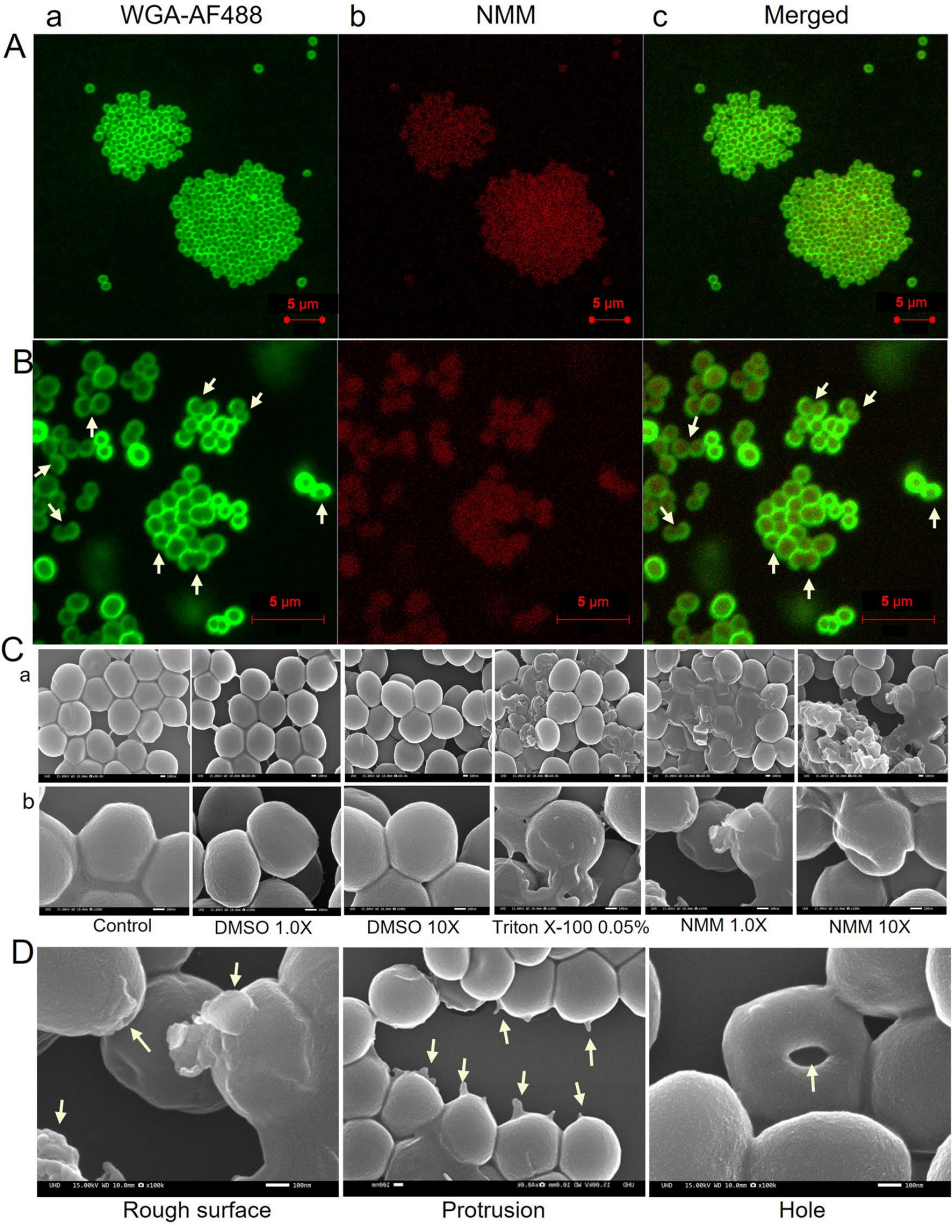
Internalization of NMM into SAUSA300 cells. **A** Efficiency of NMM internalization into SAUSA300 wherein **a** Confocal photomicrographs reveal the SAUSA300 cell boundary as delineated by WGA-AF488 stain-labeled green fluorescence. **b** The presence of red fluorescent NMM inside the cell is depicted in the red channel. **c** Merged images of the green and red channels confirm the efficient internalization of NMM into SAUSA300 cells. **B** Magnified (2 ×) images of Fig. 2A, showing the debilitation of green-fluorescent cell boundaries on incubation for 2 h at MIC_50_ indicated using arrows. **C** Scanning electron microscopy (SEM) images of SAUSA300 at 40,000 × (**a**) and 100,000 × (**b**) magnification reveal incomplete cell division and cell wall damage in SAUSA300. SAUSA300 cultures grown at 37 °C in tryptic soy broth, with an OD_600 nm_ of 1, were treated with 1.0 × and 10 × MIC of NMM for 12 h. The cell wall integrity was assessed via SEM. A 0.05% Triton X-100 solution served as a positive control for cytolysis, while untreated cells and those treated with the dimethyl sulfoxide (DMSO) carrier at 0.05% (1.0 ×) and 0.5% (10 ×) (v/v) concentrations acted as carrier controls showing unaltered bacterial cells. **D** Magnified SEM images of (2**C**) reveal various patterns of cell wall destruction in SAUSA300 cells. Each destruction pattern is indicated by arrows and labeled

Following verification of the antibacterial potential of NMM against SAUSA300, we attempted to elucidate its mechanism of action, focusing on details of NMM entry or binding to bacterial cells. NMM exhibits inherent red fluorescence (λem = 613 nm) when excited at 399 nm [63]. We used wheat germ agglutinin Alexa Fluor 488 conjugate stain (WGA-AF488) to label the bacterial cell membrane, with the cell boundaries highlighted in fluorescent green (Fig. 2A). In contrast, NMM was internalized by the SAUSA300 cells, as indicated by the distinct intracellular red fluorescence against the green-fluorescent cell boundaries (Fig. 2A). Moreover, a higher magnification revealed disrupted SAUSA300 cells at 1.0 × MIC of NMM (Fig. 2B).

Further, the effects of NMM on SAUSA300 cell morphology were elucidated using scanning electron microscopy (SEM). Based on the initial observations, we examined the effects of NMM at 1.0 × and 10 × MIC. Triton X-100 (0.05% v/v) and dimethyl sulfoxide (DMSO) were used as the positive and negative controls, respectively. DMSO, at 1 × and 10 × concentrations (0.05% and 0.5%, respectively), did not alter the cell surface and cellular morphology compared to that of the control while the positive control treated with Triton X-100 showed the cytolysis of staphylococcal cells (Fig. 2C). Notably, NMM treatment at both concentrations significantly disrupted the cells, as evidenced by the presence of holes, rough surfaces, and protrusions (Fig. 2C, D and S4 A-C). These findings indicate the vital role of NMM in compromising SAUSA300 cell wall integrity.

Our findings suggest the following key insights regarding the antibacterial action of NMM: (**a**) a decrease in the number of viable cells due to NMM-induced cell death (Fig. 1); (**b**) aggregated cells due to incomplete/inhibition of cell division at sublethal concentration of NMM (MIC_50_/0.5 × MIC) after a short exposure (2 h) (Fig. 2A); and (**c**) visible damage to the cell walls (Fig. 2C, D and Fig. S4A-C). These observations lead us to hypothesize that the interaction of G4-ligand, NMM with G4-motif(s) in the bacterial genome might inhibit gene expressions, particularly those within the *dcw* gene cluster of SAUSA300 essential for cell division and cell wall biogenesis.

### Analysis of G4-motif in the *dcw* cluster of SAUSA300

**Fig. 3:**
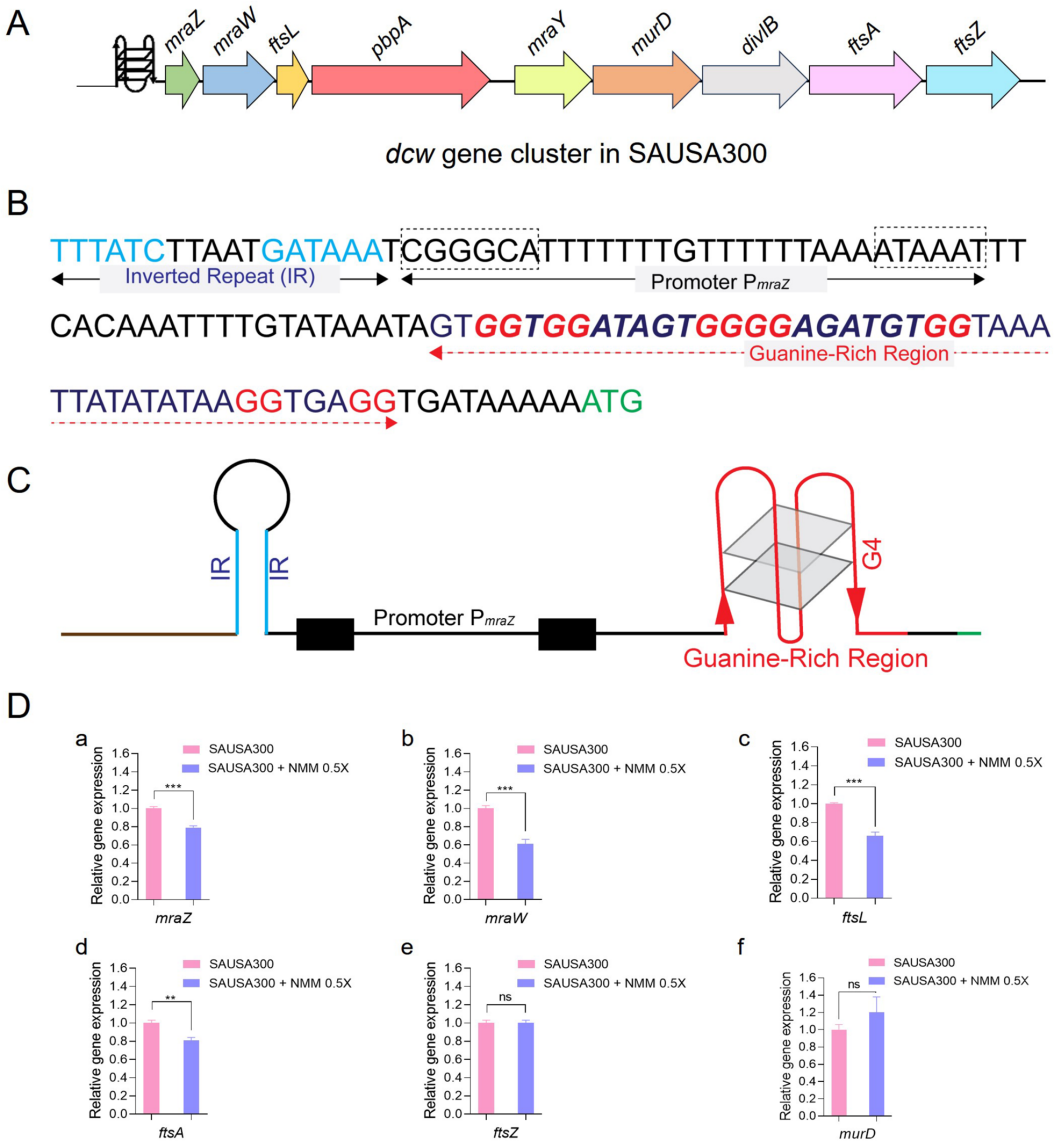
Transcriptional analysis of cell division and cell wall biosynthesis genes within the *dcw* cluster. **A** Overview of the *dcw* region in the SAUSA300 genome, including the following nine essential genes: *mraZ, mraW, ftsL, pbpA, mraY, murD, divlB, ftsA,* and *ftsZ.* **B** The upstream region of *mraZ* open reading frame (*orf*) showing inverted repeats (IR), − 10 and − 35 of P_*mraZ*_ promoter (boxed) and the guanine-rich region with the possibilities of various G4 structures. **C** Schematic representation of (**B**) with physiologically relevant G4 structure. **D** Quantitative RT-PCR analysis of *dcw* genes in SAUSA300 reveals relative gene expression levels of (**a**–**f**) *mraZ, mraW, ftsL, ftsA, ftsZ,* and *murD* between untreated SAUSA300 samples and those treated with NMM at 0.5 × MIC_90_ for 1 h. Data, averaged from triplicate experiments, are presented with standard deviations. Significance was determined using Student’s *t*-test, with *p*-values < 0.05 deemed significant (*p* > 0.05, ns = non-significant, **p < 0.01, and ***p < 0.005)

Based on the findings from CLSM and SEM analyses, it was evident that NMM exerts its antibacterial activity against SAUSA300 by affecting cell division and altering the cell wall. Consequently, we propose that NMM targets the promoter regions of genes critical for coordinated cell division and the biogenesis and maintenance of cell wall. Such gene clusters, known as the *dcw* cluster, are present across bacterial species encoding key steps in peptidoglycan synthesis and cytokinesis [64]. We identified the *dcw* region from the SAUSA300 genome [65] (GenBank Accession No. CP000255.1) and analyzed a cluster containing nine essential genes, including *mraZ, mraW, ftsL, pbpA, mraY, murD*, *divlB, ftsA,* and *ftsZ* (Fig. 3A). This cluster was examined using the G4-hunter tool [66], which identified single G4 motif in the guanine-rich upstream region of *mraZ* (Fig. S5A), the master regulator of the *dcw* cluster (Fig. 3A, B). The *mraZ* intergenic region contains putative *mraZ* promoter (P_*mraZ*_) and the guanine-rich region (40% G alone among other nucleotides) possessing more than four G-tracts downstream to the P_*mraZ*_ promoter (Fig. 3B). The P_*mraZ*_ upstream region also possesses TTTATCTTAATGATAAA, an inverted repeat, making a distinct stem-loop structure (Fig. 3B and C), which might presumably ensure the transcriptional termination of the upstream genes.

To investigate whether these G4-motifs could influence the expression of cell division and cell wall biosynthesis genes, we quantified the relative expressions of six genes (*mraZ, mraW, ftsL, ftsA, ftsZ,* and *murD*) by exposing the SAUSA300 cells to NMM at 0.5 × MIC_90_ for 1 h (Table S2). Among these, the expressions of *mraZ, mraW, ftsL,* and *ftsA* were downregulated in NMM-treated SAUSA300 compared to those in untreated controls, with reductions of 21, 39, 34, and 19%, respectively (Fig. 3D, panels a–d). However, no significant changes were noted in the expressions of *ftsZ* and *murD* (Fig. 3D, panels e, f). These results suggest that NMM may inhibit bacterial cell division and cell wall biosynthesis by downregulating *mraZ*, *mraW*, *ftsL*, and *ftsA* by potentially interacting with G4-motif downstream to the *mraZ* promoter (P_*mraZ*_), thus affecting downstream gene expression *via* the master regulator, *mraZ,* of the *dcw* cluster.

### Identification of functional P_*mraZ*__G4_3 motif and its interaction with NMM

To further elucidate the role of NMM in regulating the *dcw* cluster genes through plausible binding to G4-motifs in the P_*mraZ*_ promoter, we performed biophysical assessments of these G4 motifs and their interactions with NMM. First, circular dichroism (CD) spectroscopy was performed for the G4 sequence predicted using G4-hunter in the *dcw* cluster (P_*mraZ*__G4_1) in G4 buffer (10 mM Tris–HCl, pH 7.5 with 100 mM KCl) (Fig. S5 B). CD spectroscopic analysis revealed distinct positive and negative peaks at 265 nm and 240 nm, respectively for the intramolecular G4 structure which suggested further analysis of the melting temperature (Tm) of P_*mraZ*__G4_1. However, the Tm of P_*mraZ*__G4_1 was 33 ± 1 °C which was not a competent Tm for the G4 sequence making it physiologically irrelevant (Fig. S5 C). Hence, we then sequentially analyzed the guanine-rich region in different G-tract combinations (Fig. 3B). This further yielded an additional seven different combinations of G4 sequences with varying Quadruplex forming G-Rich Sequences (QGRS) score from P_*mraZ*__G4_2 to P_*mraZ*__G4_8 (Fig. S6 A). All the putative G4 sequences were subjected to CD spectroscopic analysis wherein P_*mraZ*__G4_7 and P_*mraZ*__G4_8 did not show the signature peak for the G4 structure (Fig. S6 B–C). Sequences P_*mraZ*__G4_2 and P_*mraZ*__G4_5 showed the CD spectra maxima at 262 and 263 nm, respectively (Fig. S6 D–E). The P_*mraZ*__G4_4 showed the hybrid G4 topology with two positive peaks at 265 and 290 nm with one negative peak at 240 nm (Fig. S6 F), while sequences P_*mraZ*__G4_3 and P_*mraZ*__G4_6 formed predominantly parallel G4 topology (Fig. S6 G–H).

Among eight predicted G4 structures, wild-type (WT) P_*mraZ*__G4_3 and P_*mraZ*__G4_6 showed parallel G4 CD spectra with a maximum peak at 265 nm and the minimum peak at 240 nm in G4 buffer (10 mM Tris–HCl, pH 7.5, and 100 mM KCl), but destabilized in buffer containing 100 mM LiCl instead of 100 mM KCl (Fig. S7A–B). As controls, we also tested few sequential mutants to attain completely destabilized G4 conformation in which key guanine repeats of WT P_*mraZ*__G4_3 and WT P_*mraZ*__G4_6 were replaced with adenine (Fig. S7A–C). The parallel G4 conformation of WT P_*mraZ*__G4_3 and P_*mraZ*__G4_6 were abolished in mutants (P_*mraZ*__ΔG4_3d and P_*mraZ*__ΔG4_6) on modifying the G residues in the G-tract (G to A) (Fig. S7A-D).

**Fig. 4:**
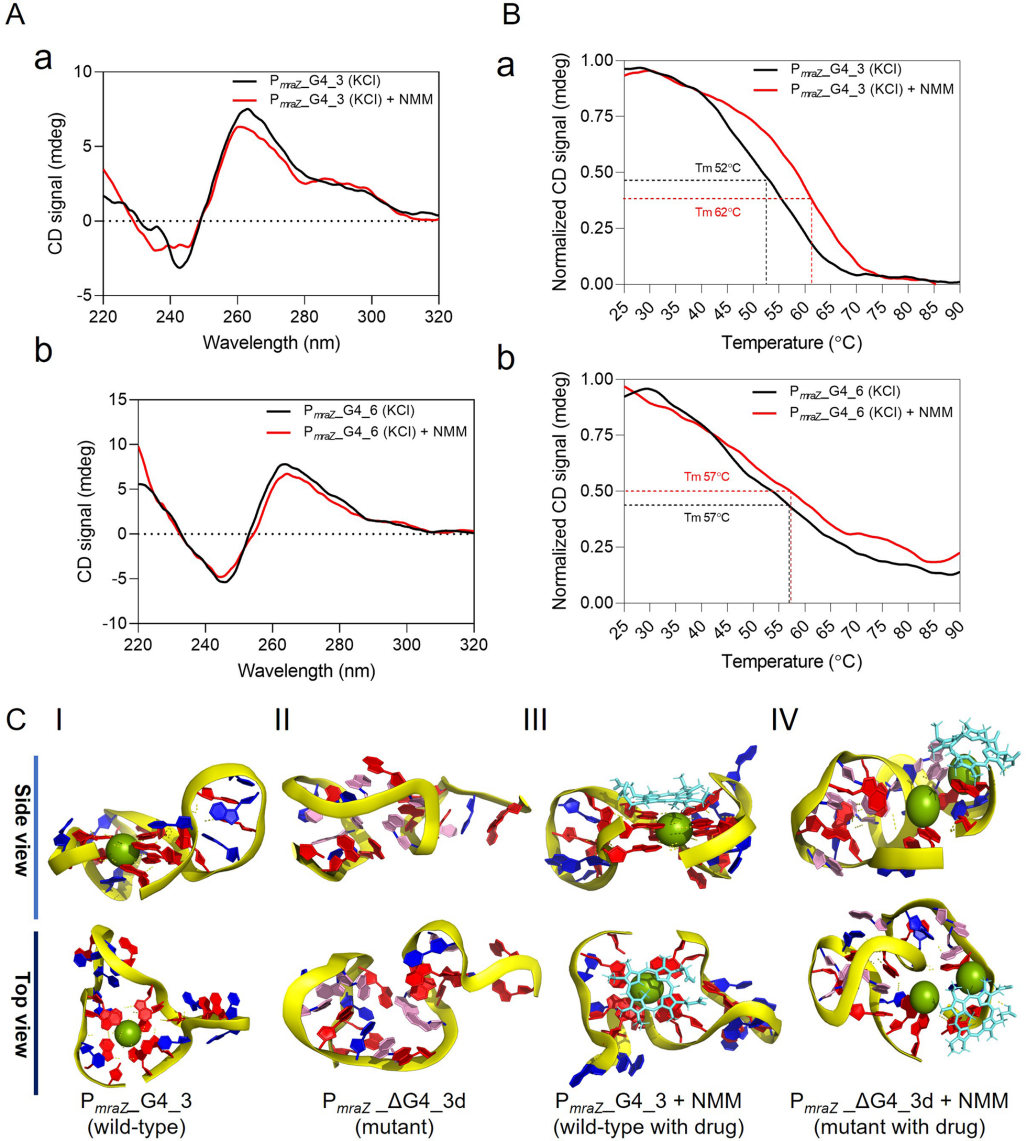
Biophysical analyses of the WT P_*mraZ*__G4_3 and P_*mraZ*__G4_6 sequences of SAUSA300. **A** (**a, b**) Circular Dichroism (CD) spectra of WT P_*mraZ*__G4_3 (**a**) and P_*mraZ*__G4_6 (**b**) with or without NMM. **B** (**a-b)** The melting temperatures of WT P_*mraZ*__G4_3 (**a**) and P_*mraZ*__G4_6 (**b**) (Tm) with or without NMM. **C** Molecular Dynamics (MD) simulation results: (I) 3D G4 conformation of WT P_*mraZ*__G4_3 demonstrating a well-structured G4 stack. (II) Destabilized, unstructured G4 conformation of the mutant *P*_*mraZ*_*_*ΔG4_3d. (III) A specific G4-stack binding mode of NMM with WT P_*mraZ*__G4_3. (IV) No binding was observed between NMM and the mutant *P*_*mraZ*_*_*ΔG4_3d

The G4 conformational stability and its signature with or without NMM treatment was analyzed among these two WT P_*mraZ*__G4 motifs (P_*mraZ*__G4_3 and P_*mraZ*__G4_6) using the CD spectra followed by melting-curve (Tm) assessment. According to our data, G4 in WT P_*mraZ*__G4_3 shifted the G4 structure from intramolecular parallel to hybrid conformation (Fig. 4A-a), while the Tm of WT P_*mraZ*__G4_3 (approximately 52 °C) increased with NMM (approximately 62 °C) (Fig. 4B-a). P_*mraZ*__G4_6 formed a stable intramolecular parallel G4 conformation in the presence of NMM (Fig. 4A-b); however, the Tm of P_*mraZ*__G4_6 (approximately 57 °C) apparently did not change upon NMM treatment (Fig. 4B-b). In CD melting curve analysis, WT P_*mraZ*__G4_3 was identified as the most suitable candidate between the two G4-motifs, as WT P_*mraZ*__G4_3 without NMM had a slightly lower Tm value (Tm approximately 52 °C) than that of WT P_*mraZ*__G4_3 with NMM (Tm approximately 62 °C), indicating G4 stabilization by NMM (Fig. 4B-a). Thus, NMM interaction increased the Tm value of WT P_*mraZ*__G4_3 suggesting G4 structural stabilization. The SYBR-Gold stained EMSA gel of WT P_*mraZ*__G4_3 showed the predominant intramolecular G4 topology while P_*mraZ*__∆G4_3a and P_*mraZ*__∆G4_3b mutants showed both intra- and inter-molecular topologies (Fig. S7E). The P_*mraZ*__∆G4_3c mutant could not be destabilized, and thus showed the G4 conformation (Fig. S7A) and migrated faster in EMSA (Fig. S7E). The P_*mraZ*__∆G4_3d mutant exhibited the slowest migration among all mutants, confirming its destabilized G4 topology (Fig. S7E). This result is consistent with the CD and Tm analysis results (Fig. S7A & D).

To assess the binding mode and energy of NMM to P_*mraZ*__G4_3, an MD simulation experiment was conducted, using the destabilized P_*mraZ*__ΔG4_3d as control. After 100 ns of simulation, the WT P_*mraZ*__G4_3 motif clearly maintained its G4 stacks, while the mutant failed to maintain the G4 stacked structure (Fig. 4C I-II), consistent with the CD and EMSA results (Fig. S7A, S7D–E). The MD simulation revealed the binding mode of NMM with WT P_*mraZ*__G4_3 (Fig. 4C III) was consistent as reported earlier [63]. As expected, the mutant P_*mraZ*__ΔG4_3d neither showed the stacked G4 structure nor any interaction with NMM (Fig. 4C IV). Furthermore, the guanine residues are symmetrically arranged around the K^+^ ion in WT P_*mraZ*__G4_3, and the intermolecular distances were typical of G4 structure (Fig. S8 A). NMM showed an efficient and specific binding through π–π stacking interaction with the structured G4 conformation of WT P_*mraZ*__G4_3 (Fig. S8B), which agrees with the earlier report [63].

To rationalize NMM’s highest binding efficiency with WT P_*mraZ*__G4_3 and to explain the role of porphyrin ring in G4 binding, we calculated the binding energy of NMM and two non-porphyrin rings containing G4-ligands, BRACO19 and Quinacrine (Fig. S8C–D). The binding energies (ΔG) of BRACO19 and Quinacrine − 32.05 ± 1.66 kcal/mol and − 24.37 ± 2.88, respectively (Fig. S8H) were higher than that of NMM (− 49.55 ± 0.72 kcal/mol), suggesting a plausible role of porphyrin ring in G4 binding. We then tested the binding of two porphyrin-containing G4-ligands, TMPyP4 (Fig. S8E), TMPyP2 (Fig. S8F), and porphyrin ring only (Fig. S8G) with WT P_*mraZ*__G4_3. Both TMPyP4 (ΔG = − 31.37 ± 1.63 kcal/mol), and TMPyP2 (ΔG = − 20.26 ± 2.02 kcal/mol) displayed intermediate binding energies while the porphyrin ring alone showed the highest binding energy (ΔG = − 10.62 ± 1.53 kcal/mol) (Fig. S8H). These findings suggest that NMM’s superior activity arises not only from the porphyrin ring but also from its highly selective and specific interaction to parallel G4 conformation as the NMM molecule lacks bulky substituents attached to the porphyrin ring. In contrast, both TMPyP2 and TMPyP4 with bulky substituents at the periphery, are known to hinder the efficient interaction with G4 conformation and exhibit differential selectivity [67]. This explains why NMM exhibits stronger binding interactions compared to other porphyrin-containing G4 ligands (TMPyP4 and TMPyP2).

### Biophysicochemical characterization of P_*mraZ*__G4_3 motif

**Fig. 5:**
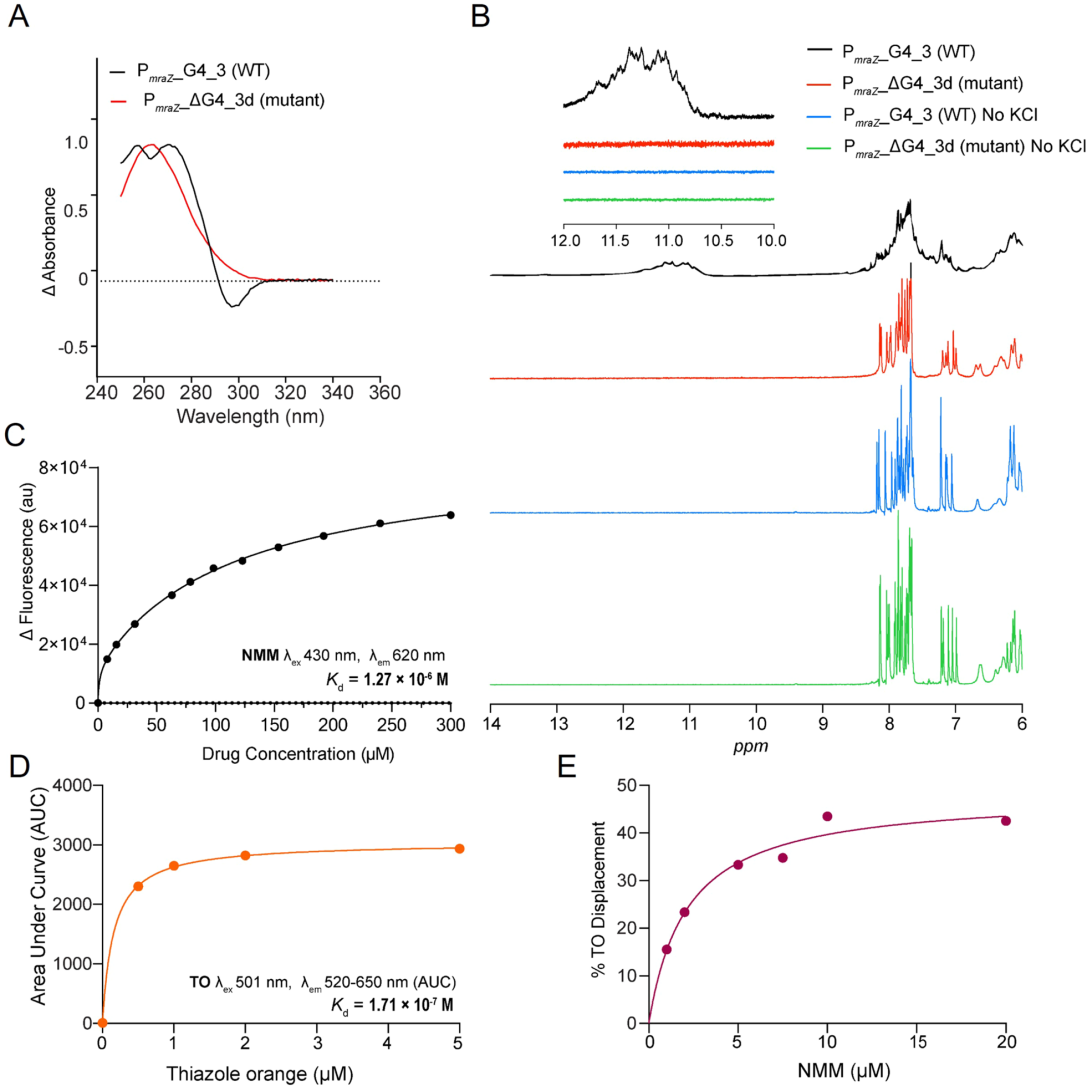
Biophysicochemical characterization and validation of the G4 conformation of WT P_*mraZ*__G4_3 and its binding with NMM. **A** Thermal difference spectra (TDS) of WT P_*mraZ*__G4_3 confirmed the G4 structure with characteristic positive and inverted peaks. **B** 1D ^1^H NMR spectra of WT P_*mraZ*__G4_3 revealed G4 structure-specific imino proton signals between 10.5–12.0 ppm, indicative of stable G4 formation. **C** Steady-state fluorescence titration assay determined a dissociation constant (*K*_d_) of 1.27 × 10⁻⁶ M for the binding of NMM to WT P_*mraZ*__G4_3, indicating strong binding affinity. **D** Thiazole orange (TO) association curve with WT P_*mraZ*__G4_3 demonstrated a strong binding constant (*K*_d_ = 1.71 × 10⁻⁷ M). **E** Competitive displacement of TO bound to WT P_*mraZ*__G4_3 by NMM further supported its specific and strong interaction with WT P_*mraZ*__G4_3

A UV–visible titration assay was performed to further investigate the interactions between NMM and WT P_*mraZ*__G4_3 in solution. NMM exhibited an absorption peak at 378 nm (λ_max_ = 378 nm) with a significant increase in absorbance upon mixing with WT P_*mraZ*__G4_3. UV–vis spectroscopic titrations of NMM with WT P_*mraZ*__G4_3 revealed a red shift of 21 nm, resulting in an absorption maximum at 399 nm (λ_max_ = 399 nm). In contrast, the mutant P_*mraZ*__ΔG4_3d, used as a control, displayed no red shift, indicating a lack of interaction with NMM (Fig. S9A & B). Furthermore, the absorption ratio at 399/378 nm plotted against [DNA]: [drug] confirmed a clear interaction between NMM and WT P_*mraZ*__G4_3, which was absent in the mutant P_*mraZ*__ΔG4_3d (Fig. S9C). The base-stacking interactions and G4 structure of WT P_*mraZ*__G4_3 were further validated using thermal difference spectra (TDS). The TDS showed a G4-specific spectrum with a positive peak at 273 nm and an inverted signature peak at 295 nm, consistent with previous reports [68]. In contrast, these G4-specific peaks were completely absent in the mutant P_*mraZ*__ΔG4_3d, indicating its inability to adopt a G4 conformation (Fig. 5A).

To further confirm G4 formation, 1D ^1^H NMR spectroscopy was performed with and without KCl for both WT P_*mraZ*__G4_3 and the mutant P_*mraZ*__ΔG4_3d. G4-specific imino proton peaks were observed between 10.5 and 12 ppm exclusively in WT P_*mraZ*__G4_3 in the presence of KCl, confirming the stable G4 structure in the P_*mraZ*_ promoter region under these conditions. Interestingly, neither the G4-specific imino proton peaks were observed in WT P_*mraZ*__G4_3 without KCl, nor in the mutant P_*mraZ*__ΔG4_3d under any conditions, confirming that the mutant sequence fails to form a stable G4 structure (Fig. 5B).

A steady-state fluorescence titration assay was performed using varying concentrations of G4 ligands listed in Table S4 (Fig. S10A–I). Among the tested ligands, NMM exhibited the highest binding affinity with WT P_*mraZ*_*_*G4_3, with a *K*_d_ value of 1.27 × 10⁻⁶ M (Fig. 5C). This high binding affinity correlated with the superior IC_50_ value, bioavailability (internalization) (Fig. S2J, Fig. S3H–H’), and antibiotic killing potential of NMM (Fig. S10J, Fig. 1). To further validate the strong interaction between NMM and WT P_*mraZ*__G4_3, we conducted competition experiments using the G-quadruplex fluorescence intercalator displacement (FID) assay, with thiazole orange (TO) as an intercalator. Titrating TO with WT P_*mraZ*__G4_3 resulted in increased fluorescence, indicative of a strong binding affinity between TO and WT P_*mraZ*__G4_3 (Fig. 5D). Curve fitting with a 1:1 stoichiometry model revealed a sub-micromolar binding coefficient (*K*_d_ = 1.71 × 10⁻⁷ M), confirming the strong binding of TO to the G4 conformation of WT P_*mraZ*__G4_3 (Fig. 5D). Further, displacement experiments showed that NMM competitively displaced approximately 40% of TO from WT P_*mraZ*__G4_3 (Fig. 5E), indicating a specific and strong interaction of NMM with the G4 conformation of WT P_*mraZ*__G4_3. These results support the hypothesis that NMM effectively targets the G4 structure, contributing to its high binding affinity and antimicrobial efficacy.

### Reporter assay to establish the role of G4-motif in P_*mraZ*_ promoter

**Fig. 6:**
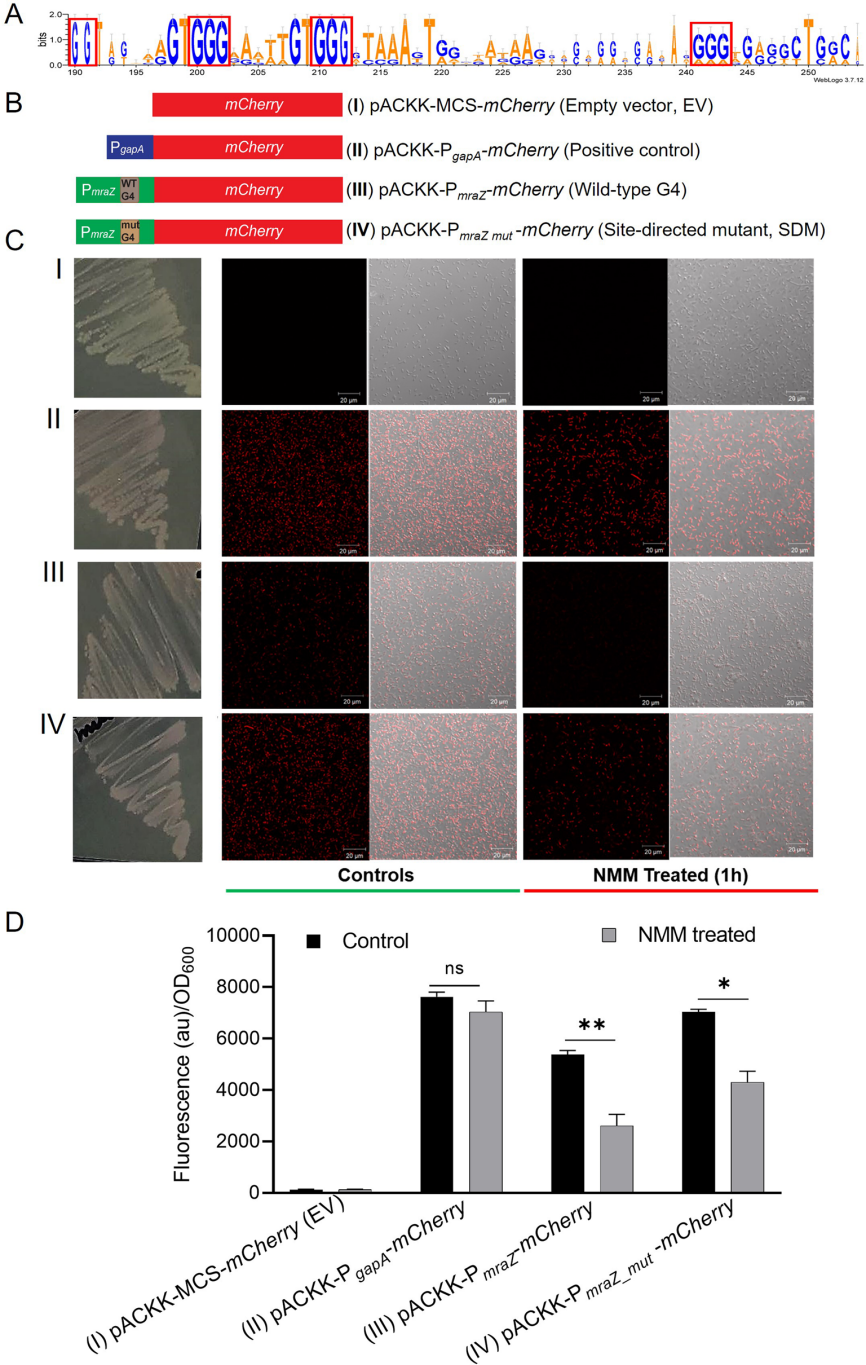
Establishing the role of G4 motif in the regulation of P_*mraZ*_ promoter. **A** Comparative analysis of the P_*mraZ*_ promoter across various gram-positive and gram-negative bacteria revealed the presence of a conserved G4 motif involved in the regulation of *mraZ*. **B** Differential expression of mCherry protein was assessed using four constructs. **C, D** Expression of *mCherry* reporter gene under various promoters upon treatment of NMM was monitored by the bacterial strains streaking (left panel) and their microphotographs (right panels). Confocal microscopy was used to qualitatively observe mCherry protein expression, no expression in promoter-less *mCherry* (**C–I**), differential expression of *mCherry* under P_*gapA*_ promoter (**C–II**), wild-type *mraZ* promoter possessing WT P_*mraZ*__G4_3 G4- motif (P_*mraZ*_) (**C–III**), and mutant promoter with destabilized G4-motifs P_*mraZ*__ΔG4_3d (P_*mraZ_mut*_) (**C–IV**). **D** Fluorometric analysis was performed to quantitatively estimate mCherry protein signals wherein a promoter-less empty vector and the P_*gapA*_ promoter served as negative and positive controls, respectively. Wild-type P_*mraZ*_ (WT P_*mraZ*__G4_3), and its mutant (P_*mraZ*___*mut*_) promoters containing destabilized G4 motifs (P_*mraZ*__ΔG4_3d) were utilized to investigate mCherry expression under optimal growth conditions and following 5 µM NMM treatment. The wild-type P_*mraZ*_ promoter possessing WT G4-motif showed the highest inhibition of mCherry protein expression upon NMM exposure indicating that the NMM stabilizes the WT P_*mraZ*__G4_3 G4 motif to inhibit mCherry reporter protein expression

MraZ acts as a regulator of *dcw* genes cluster along with other genes outside of *dcw* cluster, but no regulator is identified for P_*mraZ*_ promoter regulating *mraZ* and *mraW* in *E. coli* [69]*.* MraZ controls the transcription of *ftsL* in *Bacillus subtilis* required for focusing Z-rings to kickstart the cell separation [69, 70]. The *mraZ* and *mraW* are conserved at the 5’ end of *dcw* cluster in both gram-positive and -negative bacteria [64]. In this context, comparative analysis of the promoter region of *mraZ* across various gram-positive and -negative bacteria showed the presence of conserved G4 forming motifs (Fig. 6A and S11A–C, Table S3) plausibly involved in gene regulation of *mraZ*. A new promoter-probe vector, *pACKK*-P_*probe*_ was constructed to clone P_*mraZ*_ promoters with WT P_*mraZ*__G4_3 and mutant P_*mraZ*__ΔG4_3d G4 motifs (Fig. S12 and Fig. 6B, details in materials and methods).

All the four constructs shown in Fig. 6B (I–IV) were transformed into *E. coli* DH5α wherein promoter-less empty vector (EV) (Fig. 6B I) showed no expression of mCherry while the constructs with P_*gapA*_, P_*mraZ*_ and P_*mraZ_*mut_ (Fig. 6B II-IV) showed variable levels of mCherry fluorescent protein expressions (Fig. 6C, D). A promoter-less EV with *mCherry* reporter gene served as a negative control to assess the background or leaky expression (Fig. 6C I and D). The mCherry expression under P_*gapA*_ promoter was found to be the highest which acts as positive control for mCherry protein expression (Fig. 6C II and D) under control growth conditions. The P_*mraZ*_ promoter possessing G4-motif (WT P_*mraZ*__G4_3) showed the lowest expression of mCherry which was enhanced in P_*mraZ_mut*_ possessing destabilized G4 mutant (P_*mraZ*__ΔG4_3d) under control conditions (Fig. 6C III-IV and Fig. 4D). Considering the potent antibacterial activity of NMM, the log-phase grown cells were exposed to 5 µM of NMM for 1 h to assess the NMM-mediated G4 stabilization and consequent inhibition of mCherry protein expression (Fig. 6C, D). The highest reduction in mCherry protein expression was recorded with WT P_*mraZ*_ promoter possessing G4-motif (WT P_*mraZ*__G4_3) while a significantly lower-level reduction in mCherry expression was observed under P_*mraZ_mut*_ possessing destabilized G4 mutant (P_*mraZ*__ΔG4_3d) (Fig. 6C IV and D). In summary, the WT P_*mraZ*_ promoter containing WT G4 motif (WT P_*mraZ*__G4_3) regulated the expression of *mCherry* reporter gene, suggesting G4-motif dependent regulation of *mraZ* (Fig. 6).

### Role of G4 motif in P_*mraZ*_ promoter on transcription/translation

**Fig. 7:**
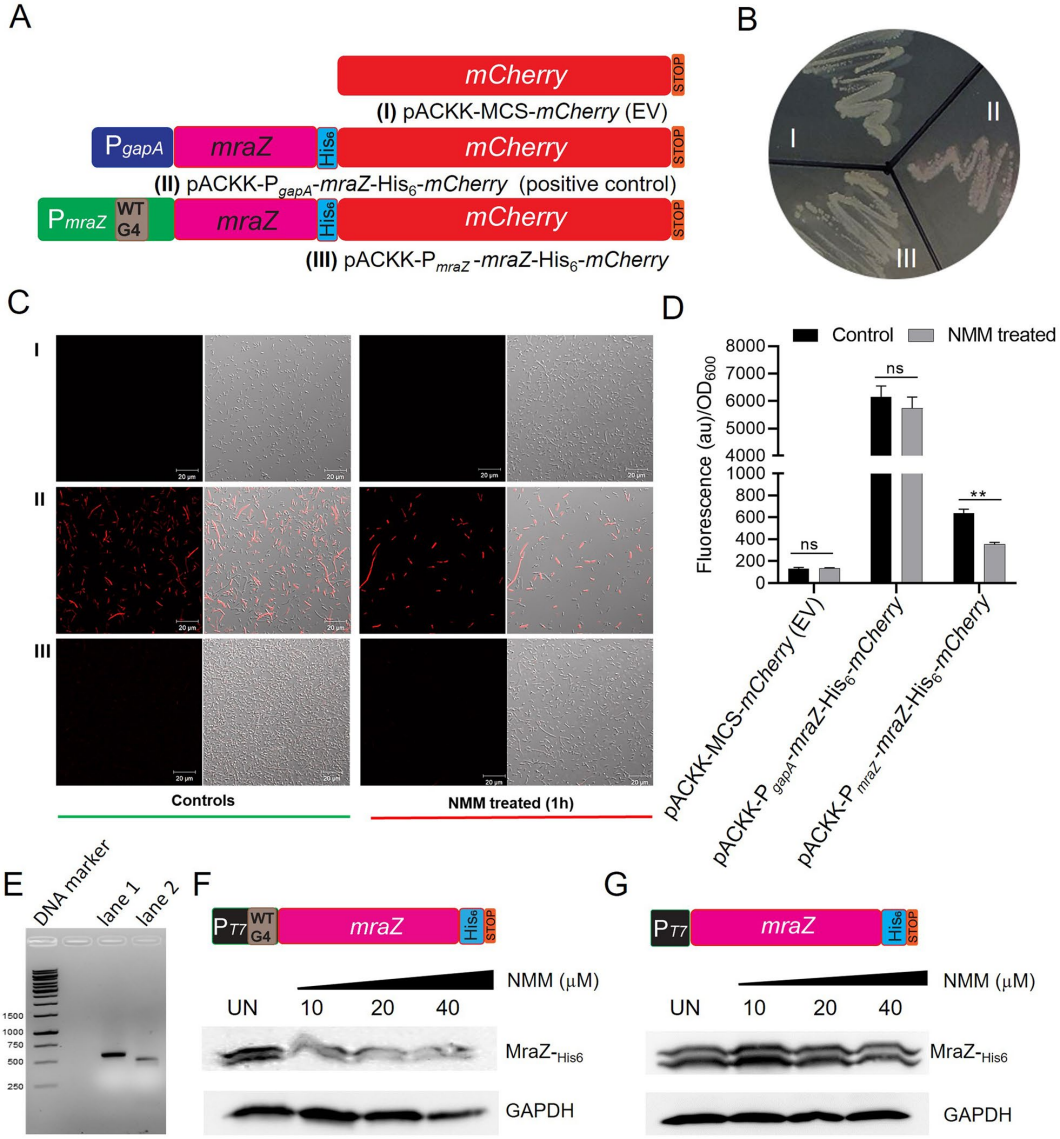
NMM stabilizes the G4-motif in P_*mraZ*_ promoter to inhibit transcription/translation. **A, B** Three constructs showing promoter-less *mCherry* empty vector (EV) (**A I**), the recombinant fusion protein, rMraZ-His_6_-mCherry expression under P_*gapA*_ and P_*mraZ*_ promoters (**A II–III**) were transformed in *E. coli* DH5α showing the differential expression of rMraZ-His_6_-mCherry fusion protein (**B I–III**). **C, D** Qualitative and quantitative assessment of differential expression of rMraZ-His_6_-mCherry fusion protein without or with 5 µM NMM using qualitative confocal fluorescence microscopy (**C I–III**); and quantitative fluorometric estimation of red fluorescent signal intensity of rMraZ-His_6_-mCherry fusion protein showing that the NMM inhibits the rMraZ-His_6_-mCherry fusion protein expression due to the inhibition of bacterial coupled transcription/translation (**D**). **E** DNA templates for IVT reactions showing T7 promoter with G4-motif (WT P_*mraZ*__G4_3) (lane 1) and T7 promoter without G4-motif (lane 2) tagged with *mraZ-*_*his6-stop*_. **F, G** Western blot and immunodetection of recombinant MraZ-_His6_ protein using Anti-His antibody showed the NMM concentration-dependent inhibition of coupled transcription/translation in case of T7 promoter with G4-motif (WT P_*mraZ*__G4_3) (**F**), while such inhibition was absent where T7 promoter is devoid of G4-motif (**G**)

To investigate the role of the G4 motif in the P_*mraZ*_ promoter on transcriptional regulation, three plasmids were constructed: a promoter-less *mCherry* empty vector (EV) (Fig. 7A I), and recombinant fusion protein rMraZ-His_6_-mCherry under the P_*gapA*_ and P_*mraZ*_ promoters (Fig. 7A II–III). These plasmids were transformed into *E. coli* DH5α, with EV and P_*gapA*_ serving as negative and positive controls, respectively, for fusion protein expression under the P_*mraZ*_ promoter (Fig. 7A, B I-III). The bacterial cultures were grown up to log-phase and then treated with 5 µM NMM, with untreated samples serving as controls. Qualitative and quantitative assessments of transcription and translation were conducted using confocal fluorescence microscopy and fluorescence intensity measurements of the recombinant MraZ-His_6_-mCherry fusion protein (λex = 587 nm; λem = 610 nm), respectively. The empty vector control (EV) showed no red fluorescence, either with or without NMM treatment (Fig. 7C I and D). In contrast, high expression level of rMraZ-His_6_-mCherry was observed under the P_*gapA*_ promoter, as indicated by red fluorescence and high fluorescence intensity, with minimal impact from NMM treatment for 1 h (Fig. 7C II and D). Under control growth conditions, the P_*mraZ*_ promoter containing the wild-type G4 motif (WT P_*mraZ*__G4_3), a lower level of rMraZ-His_6_-mCherry expression was observed, which was further diminished upon NMM treatment (Fig. 7C III and D). These results support the notion that NMM stabilizes the G4 motif in the P_*mraZ*_ promoter, leading to transcriptional inhibition of the *dcw* cluster genes, thereby inhibiting the expression of the rMraZ-His_6_-mCherry fusion protein.

To further validate the effect of NMM on bacterial coupled transcription-translation under controlled conditions, an in vitro transcription/translation (IVT) system was employed with varying concentrations of NMM (0, 10, 20, and 40 µM). IVT DNA templates containing the T7 promoter with the WT G4 motif (P_*mraZ*__G4_3, 568 bp) or without the WT G4 motif (498 bp) were generated by PCR using construct III as a template (Fig. 7E). The results demonstrated that transcription-coupled translation of the recombinant MraZ-His6 protein was inhibited in a dose-dependent manner when WT G4-motif-containing templates were used, indicating that NMM stabilizes the G4 structure, thereby suppressing transcription and translation (Fig. 7F). In contrast, no such inhibition was observed in IVT reactions using template DNA lacking the WT G4 motif (Fig. 7G). Taken together, these findings confirm that the WT G4 motif (P_*mraZ*__G4_3) in the P_*mraZ*_ promoter plays a regulatory role in bacterial coupled transcription-translation and its stabilization by NMM leads to inhibition of gene expression (Fig. 7).

### In-cell and in-vivo validation for the antibacterial activity of NMM

**Fig. 8:**
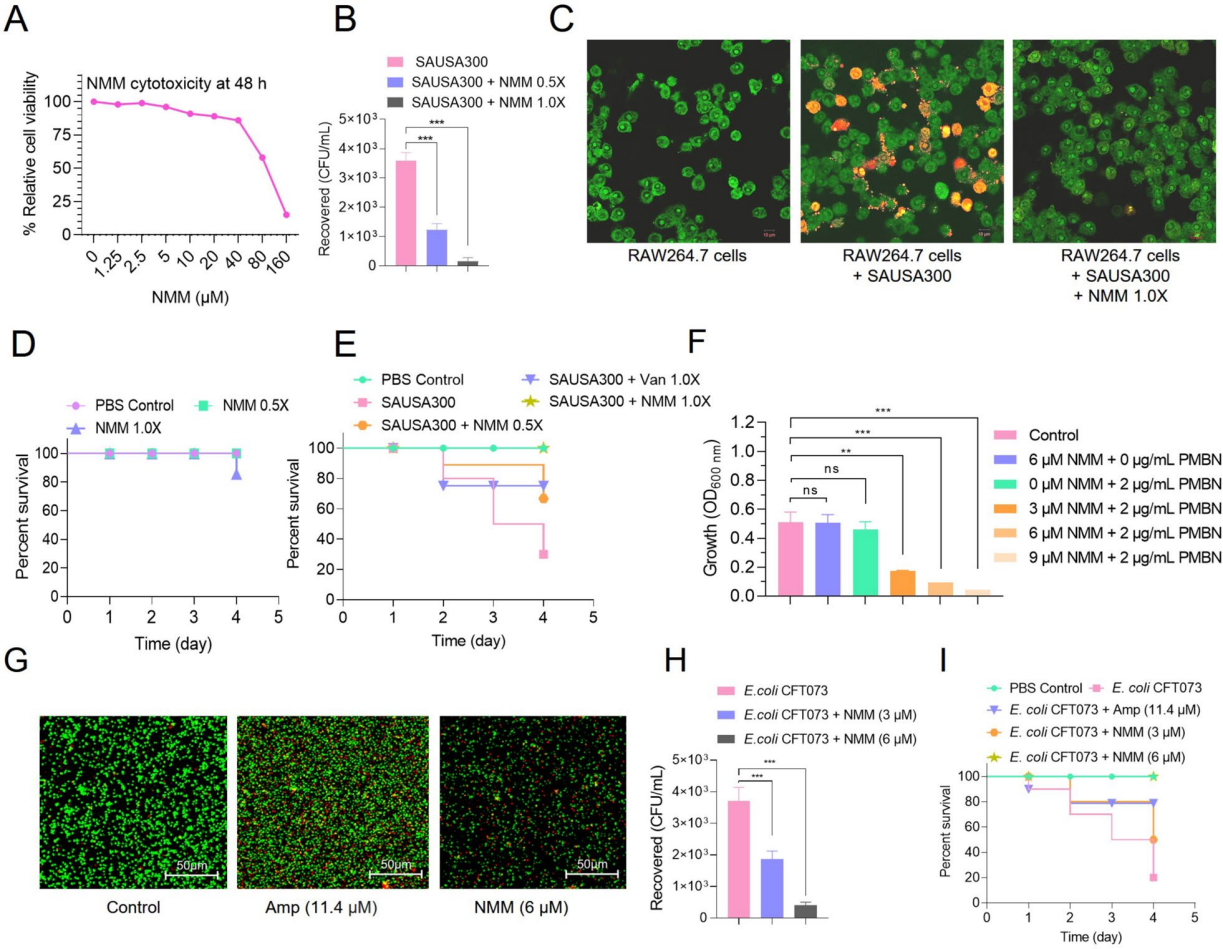
In cell and in vivo assessment of the cytotoxicity and antibacterial activity of NMM. **A** The cell toxicity of NMM by percent relative cell viability of RAW264.7 cells was measured using a WST-8 cell viability kit after 48 h of treatment with varying concentrations of NMM. **B, C** Quantitative and qualitative assessment of antibacterial activity against SAUSA300 infection in RAW264.7 cell demonstrated that NMM significantly inhibits the infection potential of hypervirulent SAUSA300 (**B**) and confocal images of RAW264.7 cells infected with SAUSA300 followed by NMM treatment at 1.0 × MIC compared to only cells and untreated infected cells showing protection from SAUSA300 infection-mediated host cell death (**C**). The scale bar is 10 μm. **D** The toxicity of NMM by percent larvae survival under in vivo conditions using *G. mellonella *(waxworms) (n = 10) showed no toxicity at 1.0 × MIC of NMM up to 4 d of monitoring. **E** The antibacterial activity NMM in *G. mellonella* larvae (n = 10) infected with SAUSA300 (2 × 10^5^ CFUs). NMM was administered 1 h post-infection followed by incubation at 37 °C to examine their survival up to day 4 wherein 1 × NMM protected 100% larvae as compared to 1 × Van. **F** Synergistic effect of NMM and PMBN against MDR gram-negative *E. coli* CFT073. PMBN (2 μg/mL) was combined with varying concentrations of NMM to determine the lowest effective NMM concentration. **G** CLSM of live/dead staining assay (SYTO9: total cells, green; PI: dead cells, red) of *E. coli* CFT073 treated with NMM (6 µM) and ampicillin (positive control: 11.4 µM) compared to untreated control cells. **H** Antibacterial activity of NMM during in vitro infection of *E. coli* CFT073 in RAW264.7 cells, showing reduced bacterial CFU recovery with increasing NMM concentrations compared to untreated cells. **I** Antibacterial activity of NMM tested in *G. mellonella* larvae (n = 10) infected with *E. coli* CFT073 (1 × 10^5^ CFUs), showing improved survival rates with NMM treatment. The significance of the data was analyzed using three independent experiments using Student’s *t*-test (*p* > 0.05 ns = non-significant, * *p* < 0.05, ** *p* < 0.01, and *** *p* < 0.005)

Our results confirmed that NMM exhibits potent antibacterial activity against SAUSA300 at the MIC of 5 μM (Figs. 1, 2, S2–S4, and Table S4). To further develop NMM and its derivatives for antibiotic use, establishing their safety profile, particularly low cytotoxicity, is imperative. Hence, we evaluated the cytotoxic effects of NMM using mouse macrophage cell line (RAW264.7), to assess its safety. The viability of RAW264.7 cells was determined by exposing them to varying NMM concentrations, to observe the dose- and time-dependent effects over periods of 6, 24 and 48 h. The WST-8 assay results revealed around 15% cytotoxicity of NMM in RAW264.7 cells, even at concentrations as high as 40 μM at 6 h, 24 h, and 48 h (Fig. 8A and Fig. S13A–B). These findings were consistently corroborated by live-dead assays using Acridine Orange/Propidium Iodide (AO/PI) staining followed by fluorescence microscopy, which further validated the minimal cytotoxic nature of NMM on mammalian cells (Fig. S13C).

Further, RAW264.7 cell line was used to assess the efficacy of NMM to eliminate infection. First, the impact of SAUSA300 on RAW264.7 cells was assessed, followed by evaluating the ability of NMM to inhibit this infection using the enzyme protection assay. Following RAW264.7 cell infection with SAUSA300 for 30 min, the cells were treated with 0.5 × MIC and 1 × MIC NMM for 2 h. To quantify the infection levels, we measured the CFUs of SAUSA300 present in infected RAW264.7 cell lysates. Our analysis revealed 65.7% and 95.3% reduction in SAUSA300 CFUs on treatment with 0.5 × and 1 × MIC of NMM, respectively (Fig. 8B).

To gain further insights into the antibacterial efficacy of NMM, we monitored the infection potential of SAUSA300 in RAW264.7 cells post-NMM treatment using CLSM. The host cell viability staining assay allowed quantification of the number propidium iodide (PI)-labeled red-fluorescent dead cells among the total SYTO9-labelled green-fluorescent cells. The findings revealed that all untreated RAW264.7 cells remained alive and green. Conversely, infected cells displayed red to orangish fluorescence, signifying cell death. Notably, no dead cells were observed on 1 × MIC NMM treatment even by searching several microscopic fields, indicating the clearance of infection due to the potent antibacterial action of NMM (Fig. 8C). These findings collectively underscore the strong antibacterial activity of NMM and thereby highlight its possible therapeutic potential.

To further assess the antibacterial activity of NMM, we conducted experiments using *Galleria mellonella* larvae (waxworms), which is widely used as an excellent infection model since its hemocytes are functionally similar to mammalian phagocytic cells [71, 72]. Before evaluating the efficacy of NMM, we determined its cytotoxicity in waxworms by administering doses equivalent to 0.5 × and 1.0 × MIC. The survival rates of 90% and 100% observed at 1.0 × and 0.5 × MIC, respectively at 4 d post-administration (Fig. 8D) indicating its minimal toxicity at concentrations where NMM demonstrates significant antimicrobial activity in vitro conditions. For the infection, we injected SAUSA300 into waxworms at 2 × 10^5^ CFU *per* larva and monitored them for 4 d. The carrier control PBS and 1.0 × MIC_90_ of vancomycin (Van) served as negative and positive controls, respectively, with an additional group of larvae with an equivalent volume of PBS injection served as a no-infection control. We documented the survival rates over 4 d post-infection (Fig. S14A). Remarkably, larvae treated with 1.0 × MIC_90_ NMM showed a 100% survival rate, outperforming the 80% survival observed with 1.0 × MIC_90_ Van, a leading antibiotic against MRSA. The delayed mortality and increased survival of larvae treated with NMM at 0.5 × and 1.0 × MIC was found to be 70% and 100%, respectively as compared to a 30% survival rate in untreated groups highlight the potent antibacterial efficacy of NMM (Fig. 8E and S14B). These results, along with those from cellular infection assays, provide compelling indications of the superior bactericidal performance of NMM against MRSA, surpassing the existing terminal antibiotics like vancomycin.

To further extend the antibacterial application, we tested the NMM binding or internalization on gram-negative MDR uropathogenic *Escherichia coli* CFT073 (UPEC). No detectable NMM internalization was visualized as intracellular red fluorescence of NMM (Fig. S15A) than that of SAUSA300 (Fig. 2A) presumably due to outer membrane permeability barrier of gram-negative bacteria. Moreover, the merged photomicrograph showing few elongated *E. coli* CFT073 cells plausibly due to inhibition of cells division at sublethal concentration of NMM (red arrow) than those of normal *E. coli* CFT073 cells (blue arrow) (Fig. S15B). Therefore, NMM was used in combination with polymyxin B nonapeptide (PMBN), as a membrane permeabilizer. Our results indicate that 2 μg/mL PMBN and 6 μM NMM effectively killed MDR *E. coli* CFT073 (Fig. 8F). The antibacterial role of NMM against *E. coli* CFT073 was further confirmed using a live/dead assay *via* CLSM. Untreated and 1.0 × MIC ampicillin (Amp)-treated *E. coli* CFT073 were used as negative and positive antibiotic controls, respectively (Fig. 8G). The untreated control group predominantly showed live cells with a negligible number of red fluorescent dead cells, while cells treated with 1.0 × MIC of AMP exhibited a higher number of dead cells with red fluorescence. Treatment with 2 μg/mL PMBN along with 6 μM NMM resulted in a greater number of dead cells than those using AMP (Fig. 8G) as the NMM could effectively kill *E. coli* CFT073 by PMBN-mediated permeabilization the outer membrane barrier. Moreover, it could protect host cells and *G. mellonella* larvae by clearing UPEC infection (Fig. 8H–I, S16A–B). These findings collectively demonstrated the efficacy of NMM as a potent broad-spectrum antibacterial agent for both MDR gram-positive and -negative bacteria.

The adaptive evolution experiment for 7d at 0.5 × MIC displayed that SAUSA300 growth rate in NMM was significantly reduced in the initial days of experiment as compared to Van-exposed and untreated controls (Fig. S17A). The 0.5 × MIC antibacterial (Van and NMM) exposed cultures were subjected to test at 1.0 × MIC for the evolution of resistant strains (Fig. S17B). SAUSA300 exposed to Van and NMM emerged as resistant strains on day 4 and day 6, respectively. Additionally, SAUSA300 growth rate remained significantly lower in NMM treatment as compared to that in Van-evolved SAUSA300 (Fig. S17B). The clonal selection and enrichment of evolved strain in NMM-evolved strain reached equal to Van-evolved strain on day 10 (Fig. S17B). The results suggest that the development of resistance rate is slower in NMM than that in Van exposure. Next, we amplified the P_*mraZ*_-promoter with *mraZ* using high fidelity Taq DNA polymerase and sequenced the region. We could not find the mutation in this region (data not shown). This result indicates that NMM might have secondary effects of its potent antibacterial activity by regulating other targets outside of *dcw* cluster. The current study remained to identify the gene mutation and/or modulation in gene expression responsible for the slower resistance development in NMM than that of vancomycin in SAUSA300. A detailed mechanism of NMM resistance development needs to be explored in future by genome sequencing followed by subtractive genomics with wild-type SAUSA300 to identify the mutations in the evolved strains.

We further assessed four representative essential genes based on lethality of their mutants [73] [*rpsF* (30S ribosomal protein S6); *glmS* (glucosamine–fructose-6-phosphate aminotransferase); *murB* (UDP-N-acetylenolpyruvoylglucosamine reductase); and *ezrA* (septation ring formation regulator)] involved in cell division and cell wall biosynthesis outside of *dcw* cluster. Three essential genes (*glmS*, *murB* and *ezrA*) were found to be downregulated with 0.5 × NMM treatment for 1 h (Fig. S18). This result suggests that NMM differentially regulates other cell-wall and cell-division related genes upon onset of antibacterial activity exerted by NMM.

## Discussion

The discovery of novel antibiotics has been stagnant for the past 25 years, primarily due to two major challenges: (a) selecting proper targets that are less susceptible to rapid development of antimicrobial resistance (AMR), and (b) enhancing chemical libraries to address the limitations in diversity [74]. In fact, the most recent novel antibacterial classes, including linezolid, daptomycin, and the topical agent retapamulin, were introduced in 2000, 2003, and 2007, respectively [74]. Monotherapy antibiotics, which target a single site, such as rifampin, streptomycin, and fusidic acid, are highly susceptible to rapid resistance development through single-step mutations. In contrast, antibiotics like fluoroquinolones and linezolid may develop resistance through stepwise mechanisms [75]. Conversely, antibiotics that target multiple sites, affecting genome-wide molecular functions, are typically less vulnerable to becoming ineffective due to single-target mutations during monotherapy [74, 76]. Thus, multitargeting antibiotics may offer reduced susceptibility to mutation. In this study, we proposed a novel approach to develop an antibiotic targeting G4, which is ubiquitously predominant in bacterial genomes.

Various G4 conformations can arise at DNA/RNA guanine-rich sequences, presenting as variable tracts (> 2–4) after certain intervals of nucleotides (N), each offering differential affinities to G4 binding drugs [77]. For example, the genome-wide analysis in this study identified 170 G4 structures in the SAUSA300 genome using G4-hunter (Threshold 1.2), though this number may vary depending on the threshold level applied [66]. We mapped these 170 G4 motifs across the SAUSA300 genome (Fig. S19A), showing their involvement in various cellular processes, including nucleic acid biosynthesis and cell envelope maintenance (Fig. S19B). These 170 G4 motifs were further categorized based on their genomic locations: 120 within coding sequences (CDS) and 50 within intergenic regions (Fig. S19C-E). The intergenic regions were subdivided into four categories to identify meaningful G4 motifs likely to be involved in gene regulation (Fig. S19D I–IV). Notably, G4 motifs located in the intergenic regions on positive or negative strands (Fig. S19D-I & S19D-II & Fig. S19E) and in divergent gene regions (Fig. S19D-IV & Fig. S19E) are most likely to be associated with gene regulation (Fig. S19A-E).

G4s are known to be involved in virulence control of pathogenic bacteria [78]. Therefore, we analyzed the SAUSA300 genome to possess G4 motif in virulence genes. Interestingly, we found four putative G4-motifs in the CDS of 3 virulence genes (Fig. S20A). The NMM exposure mildly upregulates the *esaD* and downregulates *hysA* (Fig. S20B). The *esaD* and *sdrH* showed the possibilities of parallel and hybrid G4-conformations, respectively, while the predicted G4 motif of *hysA* could not achieve G4-conformation (Fig. S20 C–F). Similarly, six representative genes containing putative G4 in their promoters were tested for the gene expression without or with NMM exposure. The aconitate hydratase encoding gene (*acnA*) and 30S ribosomal protein S16 (*rpsP*) were found to be downregulated while N-acetylglucosamine-6-phosphate deacetylase (*nagA*) and 50S ribosomal protein L13 (*rplM*) were mildly upregulated (Fig S21A). There was no effect on gene expression of *gltS* and *SAUSA300_2260* (Fig S21A). The promoters of two genes encoding *gltS* and *nagA* indicated the parallel G4 formation without NMM using preliminary CD spectra (Fig S21B–G). Taken together, the G4 conformation and the gene expression patterns of the tested genes reveal the weak correlation between the NMM-mediated changes in gene expression and their G4 structures (Fig. S21H). The observed differential gene expression could be attributed to the antibacterial activity of NMM, which likely triggers transcriptomic adjustments in metabolism and/or pleiotropic effects on gene expression. This process appears to initiate with the inhibition of cell division, coordinated with cell wall disruption. It is well known that a single antibiotic treatment can cause significant changes in the genome-wide transcriptomic profile, including both direct/specific and nonspecific/indirect alterations, as the bacteria adjust their metabolism to cope with antibiotic stress [79]. Future studies should systematically explore the genome-wide transcriptomic profiling of NMM by integrating transcriptome data with experimental confirmation of the G4 structures for all 170 predicted G4 motifs in the SAUSA300 genome.

It is difficult to fully support the claim that a single G4 ligand can exclusively target a specific G4 structure, especially given the abundance of G4 motifs present throughout the genome. While various G4-binding ligands have demonstrated specificity toward certain G4 conformations, the genome contains numerous predicted G4s that may adopt a wide range of structural conformations. Consequently, even a single G4 ligand such as NMM is likely to bind multiple G4 targets, potentially influencing both transcription and translation processes. Nevertheless, among these interactions, certain key genes—particularly transcriptional regulators—may act as primary targets that are crucial for cell survival. In this study, MraZ appears to play a central role as a transcriptional regulator, primarily affecting the expression of the *dcw* cluster, which is essential for cell division and cell wall biosynthesis.

Based on the hypothesis that bacterial G4s play crucial roles in virulence and survival, we investigated the impact of G4-binding ligands on bacterial viability. Among the tested G4-interacting ligands, NMM demonstrated the highest antibacterial activity, which is confirmed by microbial susceptibility assay, IC_50_, and drug internalization assay (Figs. 1, 2, S2–S4, and Table S4) [63]. We demonstrated that the antimicrobial effect of NMM against SAUSA300 involves targeting cell division and cell wall biosynthesis, specifically through the *dcw* gene cluster, as indicated by cell aggregation and cell wall debilitation upon treatment with sublethal concentrations of NMM (Fig. 2A–D). Further elucidation of the mechanism of action revealed that NMM downregulates the expression of key genes involved in cell wall synthesis and cell division. Bacterial cell division remains a key area of basic research for understanding bacterial growth and applying this knowledge to develop effective antibiotics. FtsZ, a protein required for cell division in most, if not all, bacteria, has been a popular target [80]. Numerous small-molecule inhibitors have been developed against the FtsZ target [74, 81] to inhibit FtsZ GTPase activity, disrupting cytokinetic Z-ring assembly and inducing bacterial lethality [82, 83]. For these reasons, targeting G4 that controls the expression of genes responsible for cell division is appropriate.

The bioinformatic analysis of the *dcw* cluster unveiled a stem-loop structure followed by a guanine-rich region, giving rise to multiple G4 motifs downstream of the promoter region of the master regulator responsible for coordinating cell division and cell wall biosynthesis, *mraZ* (Fig. 3). Remarkably, this study identified, for the first time, that the transcription of the *mraZ* regulator is governed by the G4 structure present in the upstream intergenic G-rich region (Fig. 3). Biophysical studies of a significant G4-motif revealed that WT P_*mraZ*__G4_3 adopts a stable G4 structure and binds to NMM (Fig. 5), further stabilizing its structure (Fig. 4B-a) and repressing expressions of genes in *dcw* cluster (Fig. 3D, Figs. 6, 7). Interestingly, among the various tested G4 ligands, only NMM demonstrated high antibiotic activity, which is consistent with its stronger binding affinity to G4 (Fig. S10J) and its ability to significantly increase the thermal stability of G4 (Fig. 4B-a). Through molecular dynamics (MD) studies followed by modeling analyses, we partially rationalized why NMM exhibited higher binding affinity to P_*mraZ*__G4_3, which correlates with its superior antibiotic activity (Fig. 1, Fig. S8, Fig S2-4). Based on the MD studies, we propose that both π–π stacking interactions *via* the porphyrin ring and the selective and specific interactions of NMM’s functional groups with the backbone and base atoms of P_*mraZ*__G4_3 play crucial roles in its binding to G4 (Fig. 4C, Figures S8 and S10). Recent studies suggest that the binding pocket at the G-quadruplex-duplex junction serves as a primary binding site for G4 ligands containing aromatic core structures [84, 85], which may contribute to the binding specificity of such ligands. Therefore, we cannot rule out the possibility that the formation of a G-quadruplex-duplex junction in P_*mraZ*__G4_3 may also enhance NMM's binding specificity. However, further studies are needed to experimentally validate this potential NMM binding mode with P_*mraZ*__G4_3.

In summary, our research demonstrates that NMM exhibits exceptional antibacterial efficacy, surpassing that of the traditional and last-resort antibiotic, vancomycin, which is used to treat MDR infections of both SAUSA300 and *E. coli* CFT073 (Figs. 1, 8, Fig. S14, and S16). Given the escalating challenge of antimicrobial resistance and the scarcity of novel antibiotics, the pursuit of novel targets through diverse mechanisms is imperative. We identified P_*mraZ*__G4 as a highly potential target for NMM, regulating cell division and cell wall biosynthesis, as observed in our study; however, the binding of NMM to other SAUSA300 G4 targets cannot be ruled out. Compared to vancomycin, the highly potent antibacterial activity of NMM might be attributed to its multitargeting of G4 DNA in the SAUSA300 genome. Therefore, NMM exerts a potent orchestrated antibacterial impact is less prone to develop resistance than Van (Fig. S17), as exemplified by other multitargeting antibiotics [75, 76]. Therefore, we propose that bacterial G4s are attractive targets for the development of antibiotics against MDR pathogens.

## Conclusion

In this study, we identified NMM as a high potency antibiotic compared to vancomycin under laboratory conditions and elucidated its mechanism of action, primarily targeting G4 in the *mraZ* promoter of the *dcw* gene cluster. The highest binding affinity of NMM with P_*mraZ*__G4_3 suggested a specific binding-pocket in WT P_*mraZ*__G4_3 G4-motif, which can be used as a platform for G4-drug development against MDR pathogens. Our findings not only pave the way for novel antibiotic development utilizing G4 as a robust target but also contribute to the fundamental understanding of G4-mediated regulation in coordinated cell wall biogenesis with cell division. Notably, NMM demonstrated impermeability to the outer membrane of gram-negative bacteria. While this study briefly highlights the antibacterial efficacy of NMM against gram-negative UPEC *E. coli* CFT073 using PMBN, further validation studies are warranted. Overall, NMM exhibits potent broad-spectrum antibacterial activity against both multidrug-resistant gram-positive and gram-negative bacteria.

## Supplementary Information

Additional file 1.

## Data Availability

Derived data supporting findings of this study are available from the corresponding author (K.K.K.) upon reasonable request.

## References

[1] Thurlow LR, Joshi GS, Richardson AR. Virulence strategies of the dominant USA300 lineage of community-associated methicillin-resistant *Staphylococcus aureus* (CA-MRSA). FEMS Immunol Med Microbiol. 2012;65:5–22. 22309135 10.1111/j.1574-695X.2012.00937.x PMC4090103

[2] Benson MA, Ohneck EA, Ryan C, Alonzo F III, Smith H, Narechania A, et al. Evolution of hypervirulence by a MRSA clone through acquisition of a transposable element. Mol Microbiol. 2014;93:664–81. 24962815 10.1111/mmi.12682 PMC4127135

[3] Kwiecinski JM, Horswill AR. *Staphylococcus aureus* bloodstream infections: pathogenesis and regulatory mechanisms. Curr Opin Microbiol. 2020;53:51–60. 32172183 10.1016/j.mib.2020.02.005 PMC7244392

[4] Prestinaci F, Pezzotti P, Pantosti A. Antimicrobial resistance: a global multifaceted phenomenon. Pathog Glob health. 2015;109:309–18. 26343252 10.1179/2047773215Y.0000000030 PMC4768623

[5] Ventola C. The antibiotic resistance crisis: part 1: causes and threats. Pharmacy Therapeutics. 2015;40:277–83. 25859123 PMC4378521

[6] Butler MS, Paterson DL. Antibiotics in the clinical pipeline in October 2019. J Antibiot. 2020;73:329–64. 10.1038/s41429-020-0291-8 PMC7223789 32152527

[7] Theuretzbacher U, Bush K, Harbarth S, Paul M, Rex JH, Tacconelli E, et al. Critical analysis of antibacterial agents in clinical development. Nat Rev Microbiol. 2020;18:286–98. 32152509 10.1038/s41579-020-0340-0

[8] Theuretzbacher U, Outterson K, Engel A, Karlén A. The global preclinical antibacterial pipeline. Nat Rev Microbiol. 2020;18:275–85. 31745331 10.1038/s41579-019-0288-0 PMC7223541

[9] Tacconelli E. Global priority list of antibiotic-resistant bacteria to guide research, discovery, and development. World Health Organization 2017. p. 1–7.

[10] Zheng B-X, Yu J, Long W, Chan KH, Leung AS-L, Wong W-L. Structurally diverse G-quadruplexes as the noncanonical nucleic acid drug target for live cell imaging and antibacterial study. Chem Commun. 2023;59:1415–33. 10.1039/d2cc05945b 36636928

[11] Spiegel J, Adhikari S, Balasubramanian S. The structure and function of DNA G-quadruplexes. Trends Chem. 2020;2:123–36. 32923997 10.1016/j.trechm.2019.07.002 PMC7472594

[12] Ruggiero E, Zanin I, Terreri M, Richter SN. G-quadruplex targeting in the fight against viruses: an update. Int J Mol Sci. 2021;22. 10.3390/ijms222010984 PMC8538215 34681641

[13] Bartas M, Čutová M, Brázda V, Kaura P, Šťastný J, Kolomazník J, et al. The presence and localization of G-quadruplex forming sequences in the domain of bacteria. Molecules. 2019;24:1711. 31052562 10.3390/molecules24091711 PMC6539912

[14] Harris LM, Monsell KR, Noulin F, Famodimu MT, Smargiasso N, Damblon C, et al. G-quadruplex DNA motifs in the malaria parasite *Plasmodium falciparum* and their potential as novel antimalarial drug targets. Antimicrob Agents Chemother. 2018. 10.1128/aac.01828-17. 29311059 10.1128/AAC.01828-17 PMC5826154

[15] Belmonte-Reche E, Martínez-García M, Guédin A, Zuffo M, Arévalo-Ruiz M, Doria F, et al. G-quadruplex identification in the genome of protozoan parasites points to naphthalene diimide ligands as new antiparasitic agents. J Med Chem. 2018;61:1231–40. 29323491 10.1021/acs.jmedchem.7b01672 PMC6148440

[16] Cantara A, Luo Y, Dobrovolná M, Bohalova N, Fojta M, Verga D, et al. G-quadruplexes in helminth parasites. Nucleic Acids Res. 2022;50:2719–35. 35234933 10.1093/nar/gkac129 PMC8934627

[17] Lam EYN, Beraldi D, Tannahill D, Balasubramanian S. G-quadruplex structures are stable and detectable in human genomic DNA. Nature Commun. 2013;4:1796. 23653208 10.1038/ncomms2792 PMC3736099

[18] Balasubramanian S, Hurley LH, Neidle S. Targeting G-quadruplexes in gene promoters: a novel anticancer strategy? Nature Rev Drug Discov. 2011;10:261–75. 21455236 10.1038/nrd3428 PMC3119469

[19] Cimino-Reale G, Zaffaroni N, Folini M. Emerging role of G-quadruplex DNA as target in anticancer therapy. Cure Pharm Des. 2016;22:6612–24. 10.2174/1381612822666160831101031 27587203

[20] Métifiot M, Amrane S, Litvak S, Andreola ML. G-quadruplexes in viruses: function and potential therapeutic applications. Nucleic Acids Res. 2014;42:12352–66. 25332402 10.1093/nar/gku999 PMC4227801

[21] Razzaq M, Han JH, Ravichandran S, Kim J, Bae J-Y, Park M-S, et al. Stabilization of RNA G-quadruplexes in the SARS-CoV-2 genome inhibits viral infection via translational suppression. Arch Pharm Res. 2023;46:598–615. 37563335 10.1007/s12272-023-01458-x

[22] Yadav P, Kim N, Kumari M, Verma S, Sharma TK, Yadav V, et al. G-quadruplex structures in bacteria: biological relevance and potential as an antimicrobial target. J Bacteriol. 2021. 10.1128/jb.00577-20. 33649149 10.1128/JB.00577-20 PMC8315935

[23] Shao X, Zhang W, Umar MI, Wong HY, Seng Z, Xie Y, et al. RNA G-quadruplex structures mediate gene regulation in bacteria. MBio. 2020. 10.1128/mbio.02926-19. 31964733 10.1128/mBio.02926-19 PMC6974567

[24] Alvarez-Ortega C, Wiegand I, Olivares J, Hancock RE, Martínez JL. Genetic determinants involved in the susceptibility of *Pseudomonas aeruginosa* to β-lactam antibiotics. Antimicrob Agents Chemother. 2010;54:4159–67. 20679510 10.1128/AAC.00257-10 PMC2944606

[25] Marsico G, Chambers VS, Sahakyan AB, McCauley P, Boutell JM, Antonio MD, et al. Whole genome experimental maps of DNA G-quadruplexes in multiple species. Nucleic Acids Res. 2019;47:3862–74. 30892612 10.1093/nar/gkz179 PMC6486626

[26] Mishra SK, Shankar U, Jain N, Sikri K, Tyagi JS, Sharma TK, et al. Characterization of G-quadruplex motifs in *espB*, *espK*, and *cyp51* genes of *Mycobacterium tuberculosis* as potential drug targets. Mol Ther Nucleic Acids. 2019;16:698–706. 31128421 10.1016/j.omtn.2019.04.022 PMC6531831

[27] Mishra SK, Jain N, Shankar U, Tawani A, Sharma TK, Kumar A. Characterization of highly conserved G-quadruplex motifs as potential drug targets in *Streptococcus pneumoniae*. Sci Rep. 2019;9:1791. 30741996 10.1038/s41598-018-38400-x PMC6370756

[28] Shankar U, Jain N, Mishra SK, Sharma TK, Kumar A. Conserved G-quadruplex motifs in gene promoter region reveals a novel therapeutic approach to target multi-drug resistance *Klebsiella pneumoniae*. Front Microbiol. 2020;11:1269. 32714288 10.3389/fmicb.2020.01269 PMC7344255

[29] Ramos-Soriano J, Takebayashi Y, Samphire J, O’Hagan MP, Gurr C, Heesom KJ, et al. An Azobenzene G-quadruplex ligand exhibits promising antibacterial activity against *Escherichia coli*. bioRxiv. 2022;09.506212.

[30] Cebrián R, Belmonte-Reche E, Pirota V, de Jong A, Morales JC, Freccero M, et al. G-Quadruplex DNA as a target in pathogenic bacteria: efficacy of an extended Naphthalene Diimide ligand and its mode of action. J Med Chem. 2022;65:4752–66. 34928608 10.1021/acs.jmedchem.1c01905 PMC8958502

[31] Kowalska-Krochmal B, Dudek-Wicher R. The minimum inhibitory concentration of antibiotics: methods, interpretation, clinical relevance. Pathogens. 2021;10:165. 33557078 10.3390/pathogens10020165 PMC7913839

[32] Haste NM, Hughes CC, Tran DN, Fenical W, Jensen PR, Nizet V, et al. Pharmacological properties of the marine natural product marinopyrrole A against methicillin-resistant *Staphylococcus aureus*. Antimicrob Agents Chemother. 2011;55:3305–12. 21502631 10.1128/AAC.01211-10 PMC3122406

[33] Niu H, Yee R, Cui P, Tian L, Zhang S, Shi W, et al. Identification of agents active against methicillin-resistant *Staphylococcus aureus* USA300 from a clinical compound library. Pathogens. 2017;6:44. 28930155 10.3390/pathogens6030044 PMC5618001

[34] Batool N, Ko KS, Chaurasia AK, Kim KK. Functional identification of serine hydroxymethyl transferase as a key gene involved in lysostaphin resistance and virulence potential of *Staphylococcus aureus* strains. Int J Mol Sci. 2020;21:9135. 33266291 10.3390/ijms21239135 PMC7731198

[35] Nguyen T, Kim T, Ta H. Targeting mannitol metabolism as an alternative antimicrobial strategy based on the structure-function study of mannitol-1-phosphate dehydrogenase in *Staphylococcus aureus*. MBio. 2019;10:e02660-e2718. 31289190 10.1128/mBio.02660-18 PMC6623548

[36] Schmittgen TD, Livak KJ. Analyzing real-time PCR data by the comparative C(T) method. Nat Protoc. 2008;3:1101–8. 18546601 10.1038/nprot.2008.73

[37] Ravichandran S, Razzaq M, Parveen N, Ghosh A, Kim KK. The effect of hairpin loop on the structure and gene expression activity of the long-loop G-quadruplex. Nucleic Acids Res. 2021;49:10689–706. 34450640 10.1093/nar/gkab739 PMC8501965

[38] Patro LPP, Kumar A, Kolimi N, Rathinavelan T. 3D-NuS: a web server for automated modeling and visualization of non-canonical 3-dimensional nucleic acid structures. J Mol Biol. 2017;429:2438–48. 28652006 10.1016/j.jmb.2017.06.013

[39] Schrödinger L. The PyMOL molecular graphics system. Version 2.5 ed2023.

[40] Kim S, Chen J, Cheng T, Gindulyte A, He J, He S, et al. PubChem 2023 update. Nucleic Acids Res. 2023;51:D1373–80. 36305812 10.1093/nar/gkac956 PMC9825602

[41] Hanwell MD, Curtis DE, Lonie DC, Vandermeersch T, Zurek E, Hutchison GR. Avogadro: an advanced semantic chemical editor, visualization, and analysis platform. J Cheminform. 2012;4:1–17. 22889332 10.1186/1758-2946-4-17 PMC3542060

[42] Yoo J, Aksimentiev A. New tricks for old dogs: improving the accuracy of biomolecular force fields by pair-specific corrections to non-bonded interactions. Phys Chem Chem Phys. 2018;20:8432–49. 29547221 10.1039/C7CP08185E PMC5874203

[43] Yoo J, Aksimentiev A. Improved parameterization of Amine-Carboxylate and Amine-Phosphate interactions for molecular dynamics simulations using the CHARMM and AMBER force fields. J Chem Theory Comput. 2016;12:430–43. 26632962 10.1021/acs.jctc.5b00967

[44] Wang J, Wolf RM, Caldwell JW, Kollman PA, Case DA. Development and testing of a general amber force field. J Comput Chem. 2004;25:1157–74. 15116359 10.1002/jcc.20035

[45] Wang J, Wang W, Kollman PA, Case DA. Automatic atom type and bond type perception in molecular mechanical calculations. J Mol Graph Model. 2006;25:247–60. 16458552 10.1016/j.jmgm.2005.12.005

[46] Van Der Spoel D, Lindahl E, Hess B, Groenhof G, Mark AE, Berendsen HJ. GROMACS: fast, flexible, and free. J Comput Chem. 2005;26:1701–18. 16211538 10.1002/jcc.20291

[47] Price DJ, Brooks CL III. A modified TIP3P water potential for simulation with Ewald summation. J Chemi Phys. 2004;121:10096–103. 10.1063/1.1808117 15549884

[48] Darden T, York D, Pedersen L. Particle mesh Ewald: an N⋅log(N) method for Ewald sums in large systems. J Chemi Phys. 1993;98:10089–92.

[49] Ryckaert J-P, Ciccotti G, Berendsen HJC. Numerical integration of the Cartesian equations of motion of a system with constraints: molecular dynamics of n-alkanes. J Comput Phys. 1977;23:327–41.

[50] Hess B, Bekker H, Berendsen HJC, Fraaije JGEM. LINCS: a linear constraint solver for molecular simulations. J Comput Chem. 1997;18:1463–72.

[51] Valdés-Tresanco MS, Valdés-Tresanco ME, Valiente PA, Moreno E. gmx_MMPBSA: a new tool to perform end-state free energy calculations with GROMACS. J Chem Theory Comput. 2021;17:6281–91. 34586825 10.1021/acs.jctc.1c00645

[52] Zheng B-X, Long W, Zheng W, Zeng Y, Guo X-C, Chan K-H, et al. Mitochondria-selective dicationic small-molecule ligand targeting G-quadruplex structures for human colorectal cancer therapy. J Med Chem. 2024;67:6292–312. 38624086 10.1021/acs.jmedchem.3c02240

[53] Singh A, Jain N, Shankar U, Sharma TK, Kumar A. Characterization of G-quadruplex structures in genes involved in survival and pathogenesis of *Acinetobacter baumannii* as a potential drug target. Int J Biol Macromol. 2024;269: 131806. 38670179 10.1016/j.ijbiomac.2024.131806

[54] Madeira F, Madhusoodanan N, Lee J, Eusebi A, Niewielska A, Tivey ARN, et al. The EMBL-EBI Job Dispatcher sequence analysis tools framework in 2024. Nucleic Acids Res. 2024;52:W521–5. 38597606 10.1093/nar/gkae241 PMC11223882

[55] Crooks GE, Hon G, Chandonia JM, Brenner SE. WebLogo: a sequence logo generator. Genome Res. 2004;14:1188–90. 15173120 10.1101/gr.849004 PMC419797

[56] Sambrook J. Molecular cloning: a laboratory manual. 3rd ed. Cold Spring Harbor: Cold Spring Harbor Laboratory Press; 2001.

[57] Plaut RD, Mocca CP, Prabhakara R, Merkel TJ, Stibitz S. Stably luminescent *Staphylococcus aureus* clinical strains for use in bioluminescent imaging. PLoS ONE. 2013;8: e59232. 23555002 10.1371/journal.pone.0059232 PMC3595258

[58] Kim H, Chaurasia AK, Kim T, Choi J, Ha SC, Kim D, et al. Structural and functional study of ChuY from *Escherichia coli* strain CFT073. Biochem Biophys Res Commun. 2017;482:1176–82. 27919686 10.1016/j.bbrc.2016.12.008

[59] Kim J-H, Chaurasia AK, Batool N, Ko KS, Kim KK. Alternative enzyme protection assay to overcome the drawbacks of the gentamicin protection assay for measuring entry and intracellular survival of staphylococci. Infect Immun. 2019. 10.1128/iai.00119-19. 30782857 10.1128/IAI.00119-19 PMC6479035

[60] Imdad S, Batool N, Pradhan S, Chaurasia AK, Kim KK. Identification of 2′,4′-Dihydroxychalcone as an antivirulence agent targeting HlyU, a master virulence regulator in *Vibrio vulnificus*. Molecules. 2018;23:1492. 29925801 10.3390/molecules23061492 PMC6099652

[61] Desbois AP, Coote PJ. Wax moth larva (*Galleria mellonella*): an *in vivo* model for assessing the efficacy of antistaphylococcal agents. J Antimicrob Chemother. 2011;66:1785–90. 21622972 10.1093/jac/dkr198

[62] Nguyen A, Roy JJS, Kim J-H, Yun K-H, Lee W, Kim KK, et al. Repeated Exposure of vancomycin to vancomycin-susceptible *Staphylococcus aureus* (VSSA) parent emerged VISA and VRSA strains with enhanced virulence potentials. J Microbiol. 2024;62:535–53. 38814539 10.1007/s12275-024-00139-8

[63] Yett A, Lin LY, Beseiso D, Miao J, Yatsunyk LA. N-methyl mesoporphyrin IX as a highly selective light-up probe for G-quadruplex DNA. J Porphyr Phthalocya. 2019;23:1195–215. 10.1142/s1088424619300179 PMC8356643 34385812

[64] Megrian D, Taib N, Jaffe AL, Banfield JF, Gribaldo S. Ancient origin and constrained evolution of the division and cell wall gene cluster in bacteria. Nature Microbiol. 2022;7:2114–27. 36411352 10.1038/s41564-022-01257-y

[65] Diep BA, Gill SR, Chang RF, Phan TH, Chen JH, Davidson MG, et al. Complete genome sequence of USA300, an epidemic clone of community-acquired meticillin-resistant *Staphylococcus aureus*. Lancet. 2006;367:731–9. 16517273 10.1016/S0140-6736(06)68231-7

[66] Brázda V, Kolomazník J, Lýsek J, Bartas M, Fojta M, Šťastný J, et al. G4Hunter web application: a web server for G-quadruplex prediction. Bioinform. 2019;35:3493–5. 10.1093/bioinformatics/btz087 PMC6748775 30721922

[67] Romera C, Bombarde O, Bonnet R, Gomez D, Dumy P, Calsou P, et al. Improvement of porphyrins for G-quadruplex DNA targeting. Biochimie. 2011;93:1310–7. 21689723 10.1016/j.biochi.2011.06.008

[68] Mergny JL, Li J, Lacroix L, Amrane S, Chaires JB. Thermal difference spectra: a specific signature for nucleic acid structures. Nucleic Acids Res. 2005;33: e138. 16157860 10.1093/nar/gni134 PMC1201377

[69] Eraso JM, Markillie LM, Mitchell HD, Taylor RC, Orr G, Margolin W. The highly conserved MraZ protein is a transcriptional regulator in *Escherichia coli*. J Bacteriol. 2014;196:2053–66. 24659771 10.1128/JB.01370-13 PMC4010979

[70] White ML, Hough-Neidig A, Khan SJ, Eswara PJ. MraZ transcriptionally controls the critical level of FtsL required for focusing Z-Rings and kickstarting septation in *Bacillus subtilis*. J Bacteriol. 2022;204: e0024322. 35943250 10.1128/jb.00243-22 PMC9487581

[71] Browne N, Heelan M, Kavanagh K. An analysis of the structural and functional similarities of insect hemocytes and mammalian phagocytes. Virulence. 2013;4:597–603. 23921374 10.4161/viru.25906 PMC3906293

[72] Sultan M, Arya R, Chaurasia AK, Kim KK. Sensor histidine kinases *kdpD* and *aauS* regulate biofilm and virulence in *Pseudomonas aeruginosa* PA14. Front Cell and Infect Microbiol. 2023;13: e1270667. 10.3389/fcimb.2023.1270667 PMC10595159 37881370

[73] Fey Paul D, Endres Jennifer L, Yajjala Vijaya K, Widhelm Todd J, Boissy Robert J, Bose Jeffrey L, et al. A genetic resource for rapid and comprehensive phenotype screening of nonessential *Staphylococcus aureus* genes. MBio. 2013;4:e00537-12. 23404398 10.1128/mBio.00537-12 PMC3573662

[74] Silver LL. Challenges of antibacterial discovery. Clin Microbiol Rev. 2011;24:71–109. 21233508 10.1128/CMR.00030-10 PMC3021209

[75] Woodford N, Ellington MJ. The emergence of antibiotic resistance by mutation. Clin Microbiol Infect. 2007;13:5–18. 17184282 10.1111/j.1469-0691.2006.01492.x

[76] Silver LL, Bostian K. Discovery and development of new antibiotics: the problem of antibiotic resistance. Antimicrob Agents Chemother. 1993;37:377–83. 8460908 10.1128/aac.37.3.377 PMC187680

[77] Le DD, Di Antonio M, Chan LK, Balasubramanian S. G-quadruplex ligands exhibit differential G-tetrad selectivity. Chem Commun (Camb). 2015;51:8048–50. 25864836 10.1039/c5cc02252e

[78] Harris LM, Merrick CJ. G-quadruplexes in pathogens: a common route to virulence control? PLoS Pathog. 2015;11: e1004562. 25654363 10.1371/journal.ppat.1004562 PMC4412290

[79] Muthaiyan A, Silverman Jared A, Jayaswal Radheshyam K, Wilkinson BJ. Transcriptional profiling reveals that daptomycin induces the *Staphylococcus aureu*s Cell Wall Stress stimulon and genes responsive to membrane depolarization. Antimicrob Agents Chemother. 2008;52:980–90. 18086846 10.1128/AAC.01121-07 PMC2258546

[80] Lock RL, Harry EJ. Cell-division inhibitors: new insights for future antibiotics. Nat Rev Drug Discov. 2008;7:324–38. 18323848 10.1038/nrd2510

[81] Wang J, Galgoci A, Kodali S, Herath KB, Jayasuriya H, Dorso K, et al. Discovery of a small molecule that inhibits cell division by blocking FtsZ, a novel therapeutic target of antibiotics. J Biol Chem. 2003;278:44424–8. 12952956 10.1074/jbc.M307625200

[82] Margalit DN, Romberg L, Mets RB, Hebert AM, Mitchison TJ, Kirschner MW, et al. Targeting cell division: small-molecule inhibitors of FtsZ GTPase perturb cytokinetic ring assembly and induce bacterial lethality. Proc Natl Acad Sci. 2004;101:11821–6. 15289600 10.1073/pnas.0404439101 PMC511058

[83] Beuria TK, Santra MK, Panda D. Sanguinarine blocks cytokinesis in bacteria by inhibiting FtsZ assembly and bundling. Biochem. 2005;44:16584–93. 16342949 10.1021/bi050767+

[84] Vianney YM, Preckwinkel P, Mohr S, Weisz K. Quadruplex-Duplex junction: a high-affinity binding site for Indoloquinoline ligands. Chem Eur J. 2020;26:16910–22. 32975874 10.1002/chem.202003540 PMC7756412

[85] Vianney YM, Weisz K. High-affinity binding at quadruplex-duplex junctions: rather the rule than the exception. Nucleic Acids Res. 2022;50:11948–64. 36416262 10.1093/nar/gkac1088 PMC9723630

