# Supplementary material for: Targeting the G-quadruplex as a novel strategy for developing antibiotics against hypervirulent drug-resistant Staphylococcus aureus
Source: J Biomed Sci. 2025 Feb 5;32:15. doi: 10.1186/s12929-024-01109-3 (PMC11796246; doi:10.1186/s12929-024-01109-3)
Supplement: Supplementary file 1 — Additional file 1. [file 12929_2024_1109_MOESM1_ESM.docx]

**SUPPORTING INFORMATION**

**Title**

**Targeting the G-quadruplex as a novel strategy for developing antibiotics against hypervirulent drug-resistant *Staphylococcus* *aureus***

**Running Title**

Antibacterial effect of NMM against *S.* *aureus*

**Authors**

Maria Sultan^#,1^, Maria Razzaq^#,1^, Joohyun Lee^#,1^, Shreyasi Das^#,1^, Shrute Kannappan^1^, Vinod Kumar Subramani^1^, Wanki Yoo^1^, Truc Kim^1^, Hye-Ra Lee^2^, Akhilesh K. Chaurasia^1,^ *, Kyeong Kyu Kim^1,^ *

**Affiliations**

^1^Department of Precision Medicine, Graduate School of Basic Medical Science, Institute for Antimicrobial Resistance Research and Therapeutics, Sungkyunkwan University School of Medicine, Suwon 16419, Republic of Korea.

^2^Department of Biotechnology and Bioinformatics, College of Science and Technology, Korea University, Sejong 30019, Republic of Korea.

**^#^** These authors contributed equally to this work

***Corresponding authors:**

Akhilesh Kumar Chaurasia and Kyeong Kyu Kim

Email: [chaurasia@skku.edu](mailto:chaurasia@skku.edu), [kyeongkyu@skku.edu](mailto:kyeongkyu@skku.edu)

Tel.: +82-31-299-6152

Fax: +82-31-299-6159

**TABLE OF CONTENTS**

1. **Figure S1.** Structural formulae for the G4-binding ligands screened in this study.
2. **Figure S2.** IC_50_ of G4-binding ligands against SAUSA300.
3. **Figure S3.** Drug internalization of G4-binding ligands inhibiting SAUSA300 growth.
4. **Figure S4.** Detailed images of cell wall disruptions by NMM.
5. **Figure S5.** G4-hunter analysis of division cell wall (*dcw*) cluster synchronously controlling cell division and cell wall biogenesis in SAUSA300.
6. **Figure S6.** Identification of *mraZ***_**G4 target by screening seven putative G4 sequences using CD spectra.
7. **Figure S7.** Confirmation of G4 structures of two shortlisted candidates.
8. **Figure S8.** Non-porphyrin and porphyrin rings containing G4-ligands binding with wild-type P*_mraZ_*_G4_3 with their respective binding energies.
9. **Figure 9.** UV-Visible titration of wild-type and mutant G4 sequences with NMM in solution.
10. **Figure S10.** Steady-state fluorescence titration assay showing the *K*_d_ values.
11. **Figure S11.** Conservation of G4 regions in P*_mraZ_* promoter region of *dcw* cluster in various bacterial strains.
12. **Figure S12.** Vector maps of promoter-less vector and promoter-probe vector, pACKK_P*_Probe_* for *E. coli* and *S. aureus* strains.
13. **Figure S13.** Assessment of NMM cytotoxicity in RAW264.7 cells using the WST-8 assay at 6 and 24 h, followed by microscopy.
14. **Figure S14.** SAUSA300 infection to waxworm.
15. **Figure S15.** Phenotypic assessment of *Escherichia coli* CFT073 cells treated with NMM.
16. **Figure S16.** *E. coli* CFT073 infection to waxworm.
17. **Figure S17.** Comparative assessment of resistance development upon sustained exposure of vancomycin or NMM.
18. **Figure 18.** Impact of NMM on four representative essential genes of SAUSA300.
19. **Figure S19.** Genome-wide analysis of GQ/G4 motifs present in conserved domain sequences and intergenic regions.
20. **Figure S20.** G4 motifs present in virulence genes of SAUSA300 and their gene expressions analyses using qRT-PCR followed by CD spectral evaluation to validate G4 conformation of predicted putative G4s without or with NMM.
21. **Figure 21.** G4 motifs in the promoter of representative essential and non-essential genes and their gene expressions analyses using qRT-PCR followed by CD spectral analyses to validate G4 conformation of the predicted putative G4s without or with NMM.

**Supplementary Tables**

**Supplementary Table S1.** Bacterial strains used in this study.

**Supplementary Table S2.** Primers used in the study.

**Supplementary Table S3.** Gram-positive and gram-negative bacterial strains used for conservation of putative G4-motifs in P*_mraZ_* promoters (Figure S11 and Fig. S6A) with their respective GenBank ID and Assembly ID.

**Supplementary Table S4.** Preliminary screening of antibacterial properties of 10 representative G4-binding ligands against SAUSA300.


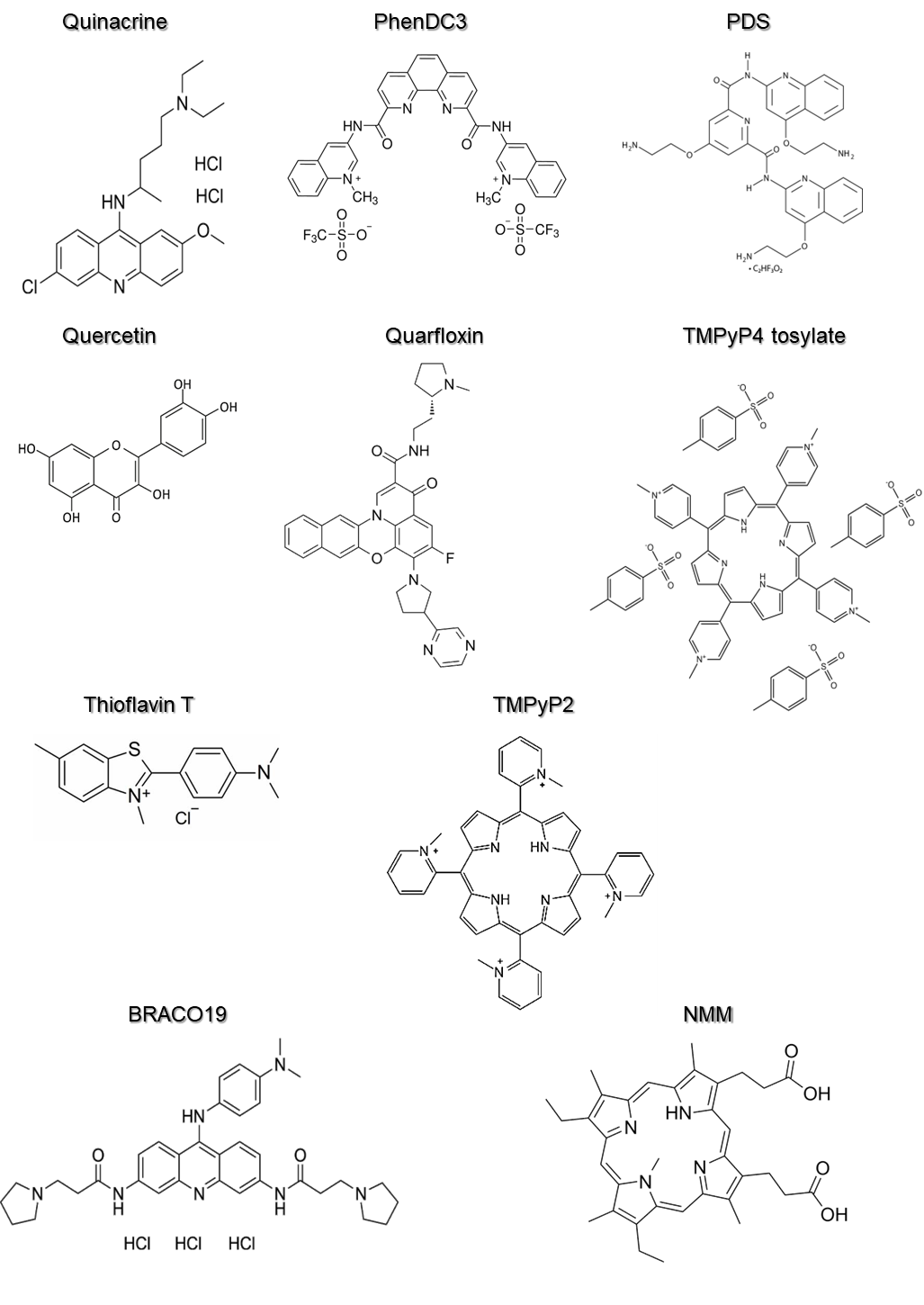


**Figure S1**. **Structural formulae for the G4-binding ligands screened in this study.** These include Quinacrine, PhenDC3, PDS (Pyridostatin), Quercetin, Quarfloxin, TMPyP4 tosylate, Thioflavin T, TMPyP2, BRACO19, and NMM.


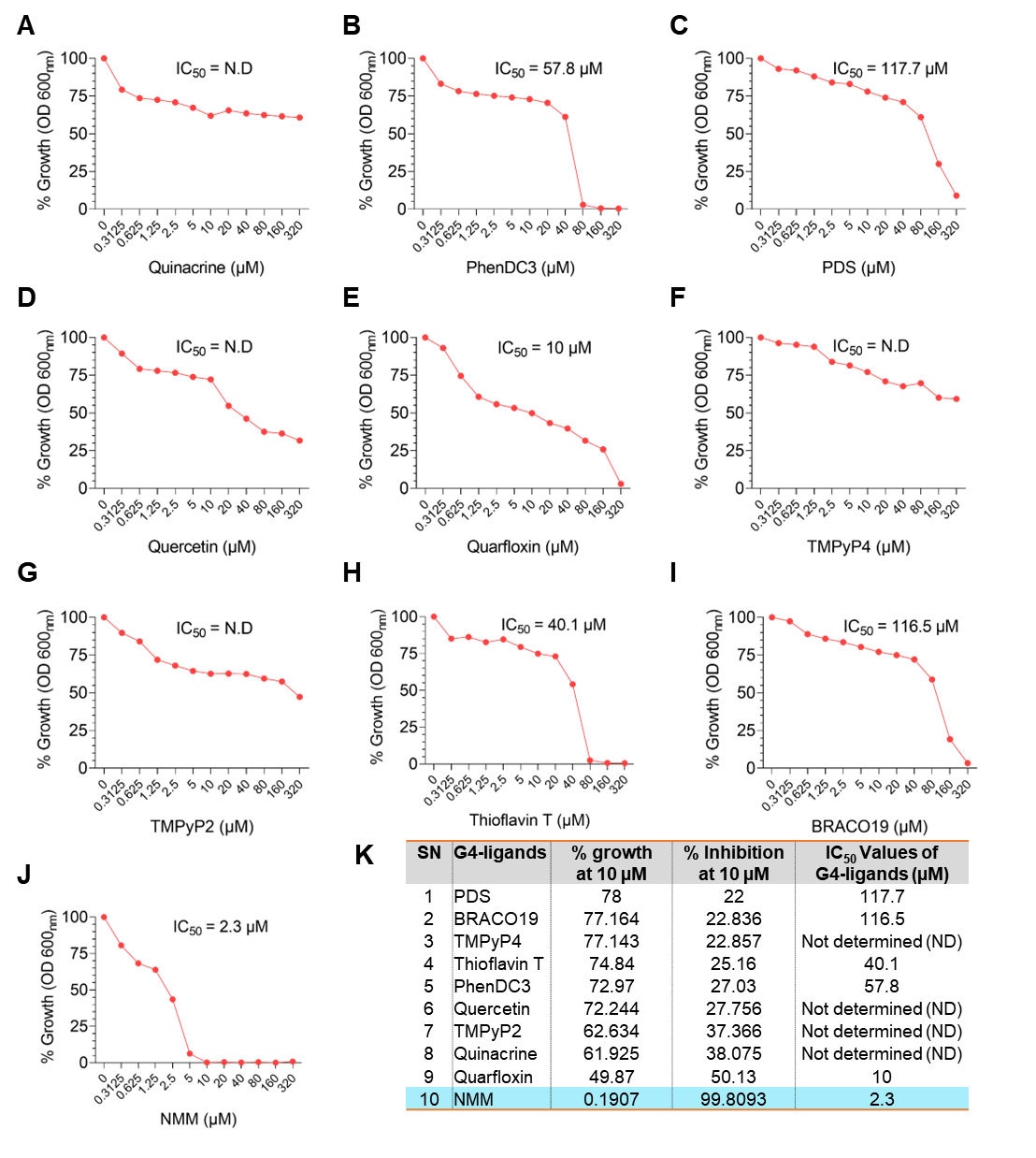
**Figure S2**. **IC_50_ of G4-binding ligands against SAUSA300.** The IC_50_ of the 10 G4-binding ligands shown as (**A**) Quinacrine, (**B**) PhenDC3, (**C**) PDS, (**D**) Quercetin, (**E**) Quarfloxin, (**F**) TMPyP4, (**G**) TMPyP2, (**H**) Thioflavin T, (**I**) BRACO19 and (**J**) NMM. The IC_50_ was determined using 2-fold increasing concentrations from 0 µM to 320 µM against SAUSA300 by following the Clinical & Laboratory Standard Institute (CLSI) protocols for testing antimicrobial susceptibility (M100: Performance standard for antimicrobial susceptibility testing) [1]. The initial inoculum of 5 × 10^5^ bacterial cells was used in 100 µL Mueller Hinton Broth (MHB) media in 96-well microtiter plate. The plates were incubated for 16 h, and optical density (OD) was measured at 600 nm using a multi-plate reader. All the experiments were done in triplicates, and the percent inhibition of SAUSA300 growth was calculated to measure the IC_50_ of each drug (ND = not determined). (**K**). The tabulated datasheet from IC_50_ data showing the percent inhibition of SAUSA300 cells at 10 µM of G4-ligands and their IC_50_ values.


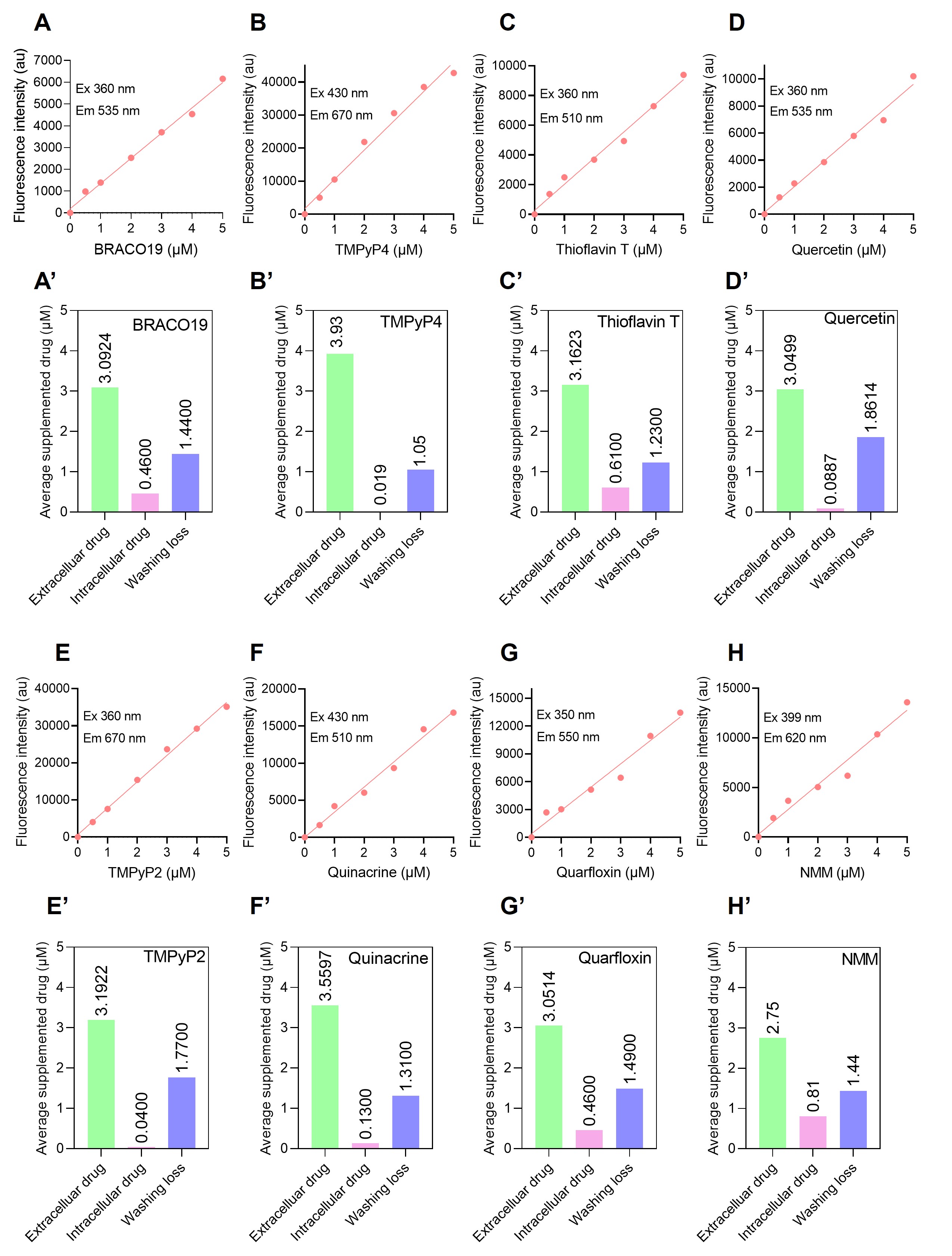


**Figure S3**. **Drug internalization of G4-binding ligands inhibiting SAUSA300 growth.** Drug permeabilization of the G4-binding ligands in SAUSA300 was calculated using their corresponding standard curves. The standard curves of eight G4-binding ligands were plotted with increasing concentrations of G4-ligands (0 to 5 µM) in 100 µL Tryptic Soy Broth (TSB) media *versus* their proportional emission (Em) fluorescence intensity at their respective excitation (Ex) wavelengths. Due to nonlinearity of PDS and PhenDC3 emission fluorescence at (0 to 5 µM) concentration, the standard graph could not establish which renders the inability to measure drug internalization. Briefly, SAUSA300 (OD_600nm_ = 1) was exposed to 5 µM of each drug for short period for 2 h in TSB media. Shorter exposure was chosen to avoid drug degradation/metabolism. The G4-ligands exposed SAUSA300 cells were pellet down by centrifugation at 4000 rpm (2701 ×*g*) for 10 min at 4 °C. The bacterial pellet and supernatant were separated into 1.5 mL Eppendorf tubes, and the bacterial pellet was washed and resuspended in 1 mL of fresh TSB media. 100 µL of each cell-suspension (Intracellular drug) and supernatant (Extracellular drug) was dispensed into 96-microtiter fluorescence plates, and emission (Em) fluorescence intensity was measured by exciting them at their respective excitation (Ex) wavelength. Based on the standard curve, intracellular drug, extracellular drug, and washing loss were calculated. (**A-H’)** showed the standard and its quantification graphs; (**A & A’**) BRACO19, (**B & B’**) TMPyP4, (**C & C’**) Thioflavin T, (**D & D’**) Quercetin, (**E & E’**) TMPyP2, (**F & F’**) Quinacrine, (**G & G’**) Quarfloxin, and (**H & H’**) NMM. The values of extracellular, intracellular and the washing loss of drug are shown with number in micromolar (µM) concentrations on bars of G4 ligands. This analysis showed that the maximum amount of intracellular concentration/bioavailability of NMM was well-corroborated with its potent antibacterial activity.


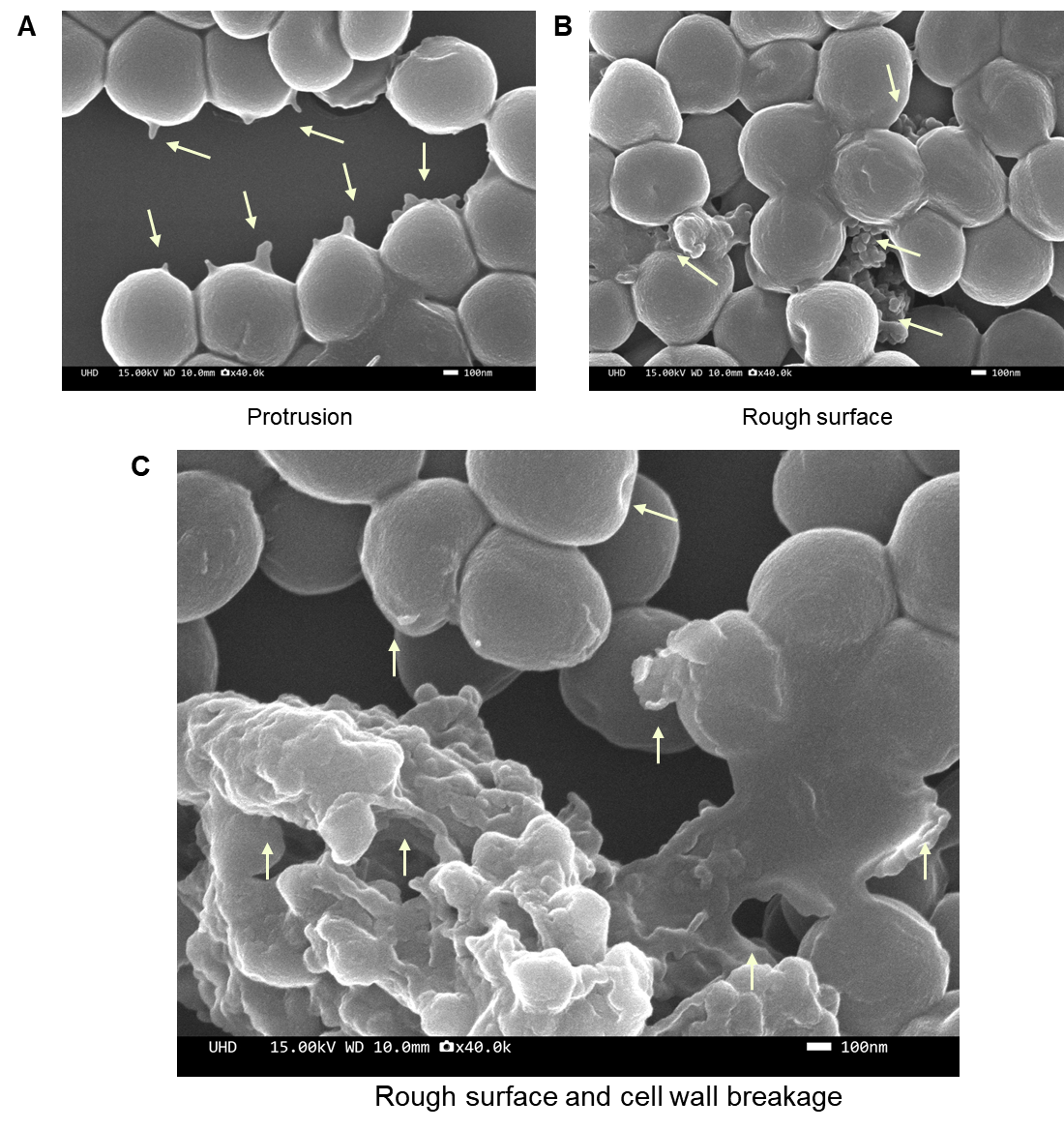


**Figure S4**. **Detailed images of cell wall disruptions by NMM.** Detailed images of wall disruptions by NMM are indicated by arrows. (**A**) Protrusion, (**B**) Rough surface, (**C**) Rough surface and cell wall breakage.


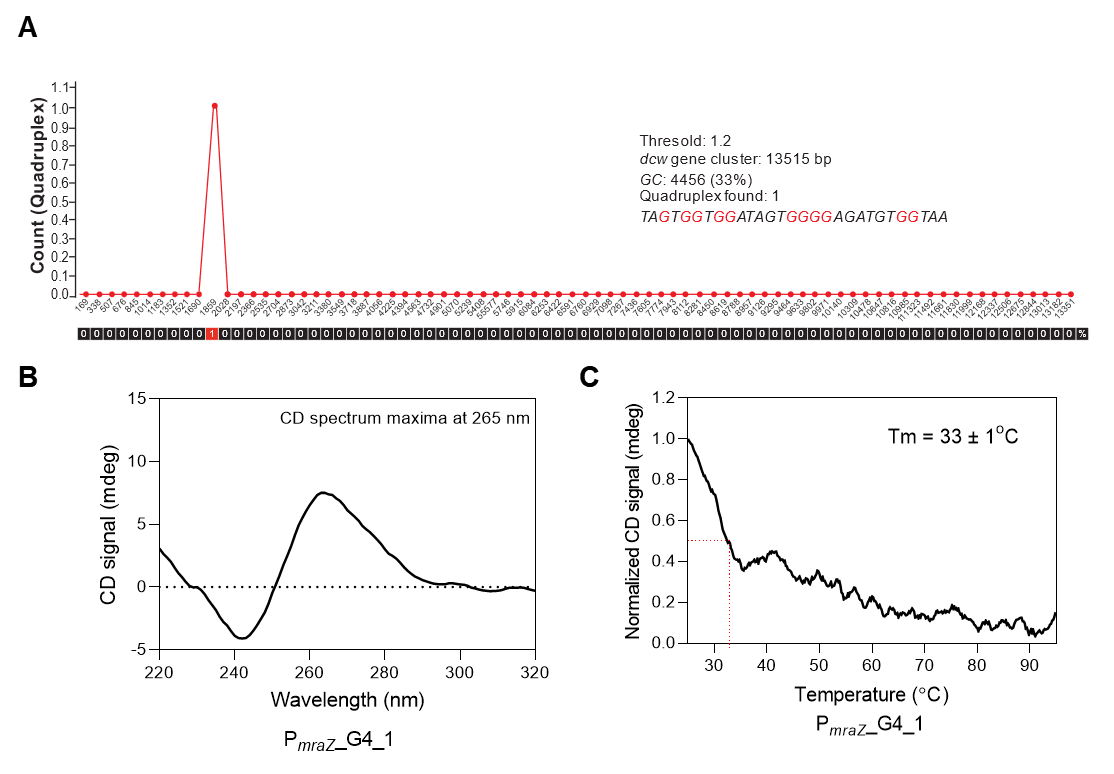


**Figure S5. G4-hunter analysis of division cell wall (*dcw*) cluster synchronously controlling cell division and cell wall biogenesis in SAUSA300.** **(A)** G4-hunter analysis of nucleotide sequence from 1170221–1183772 (13551 bp) containing the *dcw* cluster of the SAUSA300 genome showed one G4 sequence P*_mraZ_*_G4_1. (**B**) Circular dichroism (CD) spectra analysis of P*_mraZ_*_G4_1. (**C**) Melting temperature (Tm) analysis of P*_mraZ_*_G4_1. This analysis showed that the predicted G4-motif P*_mraZ_*_G4_1 possesses physiologically irrelevant low melting temperature (33 ± 1 °C) than the physiological temperature at which SAUSA300 (30-37 °C) can divide and grow.

**
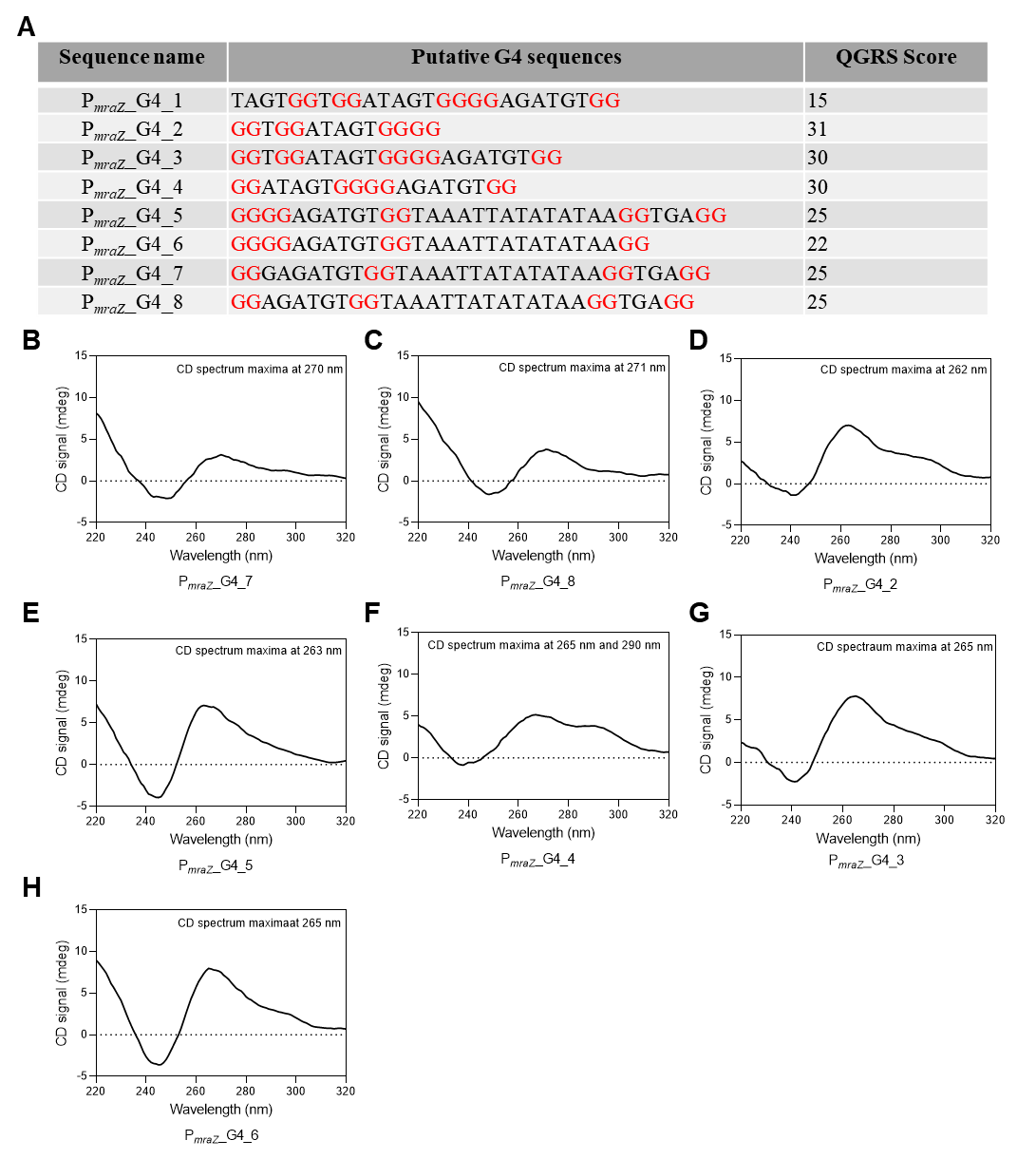
Figure S6.** **Identification of *mraZ*_G4 target by screening seven putative G4 sequences using CD spectra.** Biophysical analyses of the seven P*_mraZ_*_G4 sequences (P*_mraZ_*_G4_2 to P*_mraZ_*_G4_8) of SAUSA300 using CD spectroscopy without NMM. (**A**) The table shows the putative G4 sequences with their varying Quadruplex forming G-Rich Sequences (QGRS) scores. **(B**) P*_mraZ_*_G4_7, **(C**) P*_mraZ_*_G4_8, **(D**) P*_mraZ_*_G4_2, **(E**) P*_mraZ_*_G4_5, (**F**) P*_mraZ_*_G4_4, **(G**) P*_mraZ_*_G4_3, and (**H**) P*_mraZ_*_G4_6.

**
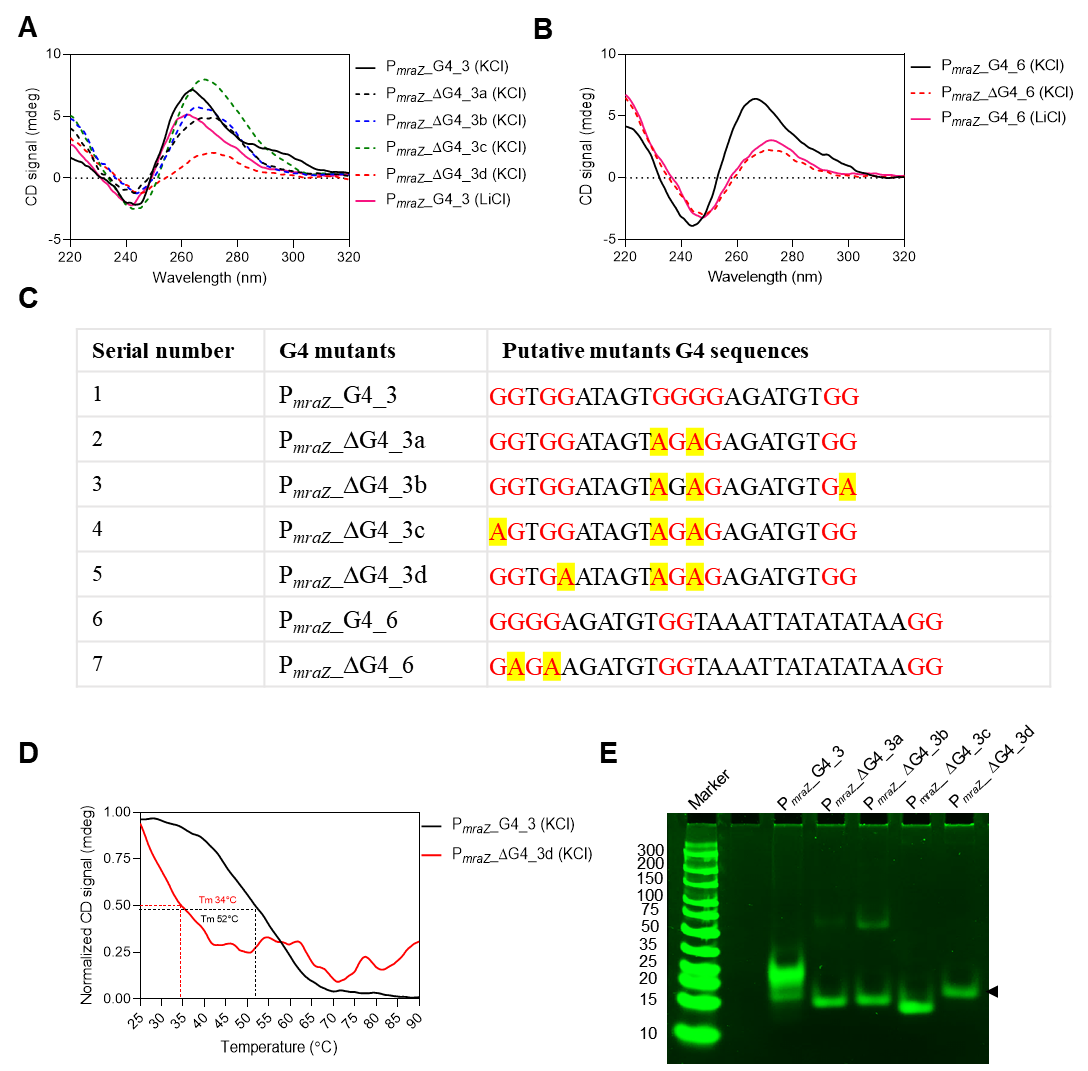
**

**Figure S7. Confirmation of G4 structures of two shortlisted candidates.** CD spectra of two shortlisted wild-type (WT) G4 candidates (P*_mraZ_*_G4_3 and P*_mraZ_*_G4_6) were confirmed by using various mutants in G4 stabilizing KCl and destabilizing LiCl buffers. (**A**) WT P*_mraZ_*_G4_3 and its sequential mutants (P*_mraZ_*_∆G4_3a – P*_mraZ_*_∆G4_3d) to achieve completely abolished G4 conformation, and (**B)** WT P*_mraZ_*_G4_6 and its mutants showing varying levels of stabilized and destabilized G4 conformations. (**C**) Table showing the putative mutant sequences of P*_mraZ_*_G4_6 and P*_mraZ_*_G4_3 wherein the yellow-highlighted ‘A’ nucleotides were the replaced ‘G’ nucleotides of the wild-type sequences. (**D**) Melting curve analysis of WT P*_mraZ_*_G4_3 and mutant P*_mraZ_*_∆G4_3d showed the Tm values 52 °C and 34 °C, respectively. This result indicates that the mutant P*_mraZ_*_∆G4_3d possesses destabilized G4 confirmation at physiological temperature. (**E**) SYBR-Gold-stained electrophoretic mobility shift assay (EMSA) gel showing the intramolecular G4 topology of WT P*_mraZ_*_G4_3, while various mutants showed deferentially destabilized topologies wherein P*_mraZ_*_∆G4_3d displayed the most destabilized G4 topology supported by CD and Tm analysis.


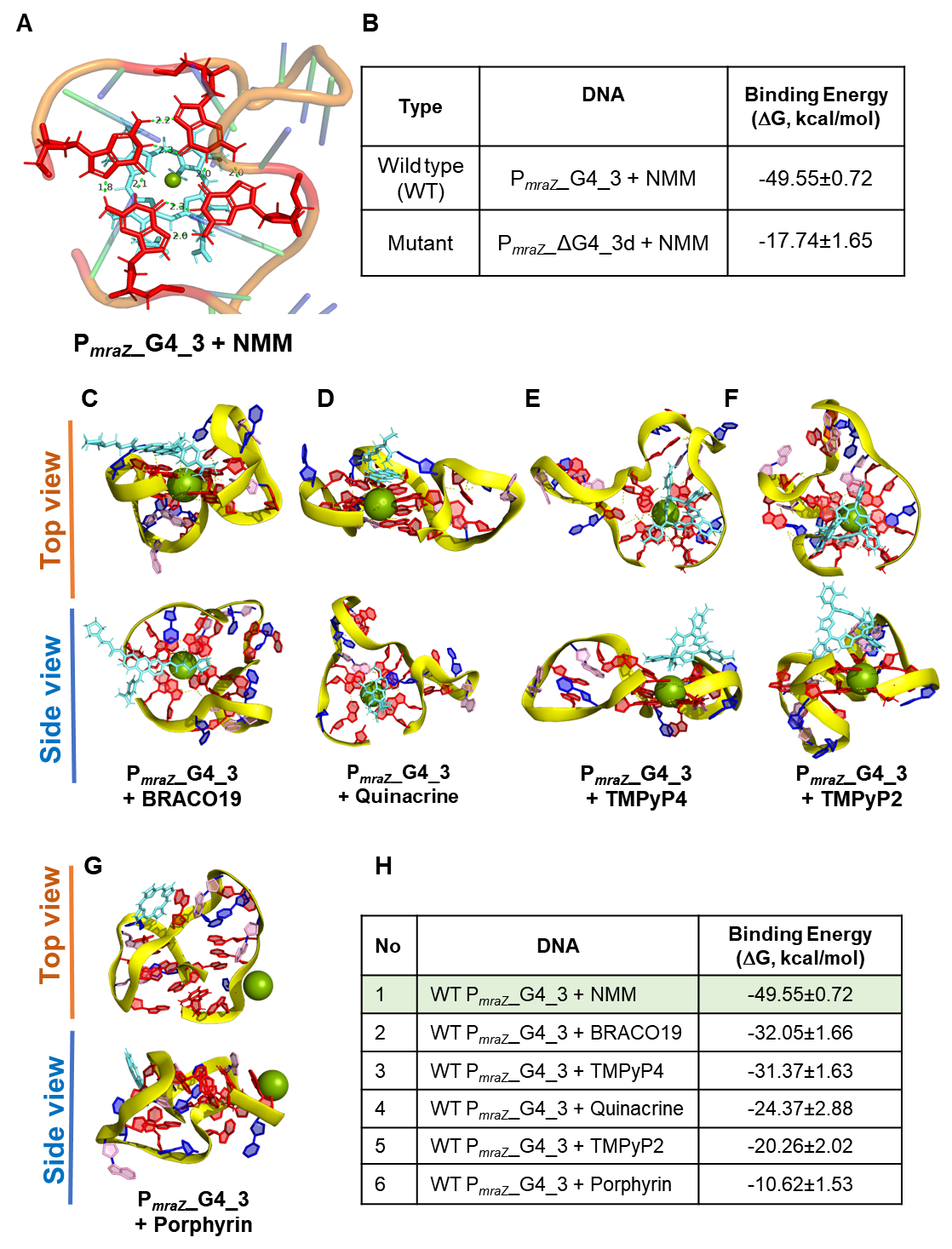


**Figure S8. Non-porphyrin and porphyrin rings containing G4-ligands binding with wild-type P*_mraZ_*_G4_3 with their respective binding energies.** (**A**) Simulated model of wild-type P*_mraZ_*_G4_3 with NMM bound with its binding mode through π–π stacking interaction with WT P*_mraZ_*_G4_3 conformed G4 structure showing the guanine residues are symmetrically arranged around the K^+^ ion and the intermolecular distances are consistent with that commonly observed in G4. (**B**) Binding energy of NMM with wild-type P*_mraZ_*_G4_3 displayed strong and specific binding as compared to mutant P*_mraZ_*_ΔG4_3d G-quadruplex. (**C–D**) Two non-porphyrin rings containing G4-ligands BRACO19 (**C**) and Quinacrine (**D**) were modeled to compare their binding mode and energy of NMM binding with wild-type P*_mraZ_*_G4_3. These two non-porphyrin rings containing G4-ligands BRACO19 (ΔG = -32.05 ± 1.66 kcal/mol) (**C**), and Quinacrine (ΔG = -24.37 ± 2.88 kcal/mol) (**D**) were used as comparator for binding energy which were found to be higher than that of NMM. Remarkably, NMM was found to possess the lowest binding energy (-49.55 ± 0.72 kcal/mol) favoring its efficient interaction with wild-type P*_mraZ_*_G4_3 showing the plausible role of porphyrin ring of NMM. To rationalize why the NMM has higher binding affinity as compared to other porphyrin ring containing G4 ligands, we modelled two additional porphyrin ring containing G4-ligands with bulky substituents namely TMPyP4 (**E**), and TMPyP2 (**F**) along with porphyrin ring only (**G**). Both the porphyrin ring containing G4-ligands *i.e.* TMPyP4 (ΔG = -31.37 ± 1.63 kcal/mol), and TMPyP2 (ΔG = -20.26 ± 2.02 kcal/mol) displayed intermediate binding energy which is in complete agreement with their steady-state binding affinity (*K*_d_) determined in figure S10 while the porphyrin ring alone showed the highest binding energy (ΔG = -10.62 ± 1.53 kcal/mol) (**H**). These findings suggest that NMM’s superior activity arises not only from the porphyrin ring but also from NMM’s specific interaction with G4, lacking bulky functional groups attached to the porphyrin ring compared to TMPyP2 and TMPyP4. Moreover, NMM has been experimentally proven to be highly selective to parallel G4 conformations while TMPyP2 and TMPyP4 possessing bulky substituents at the periphery are known to hinder the efficient interaction with G4 conformation with differential affinity [2].


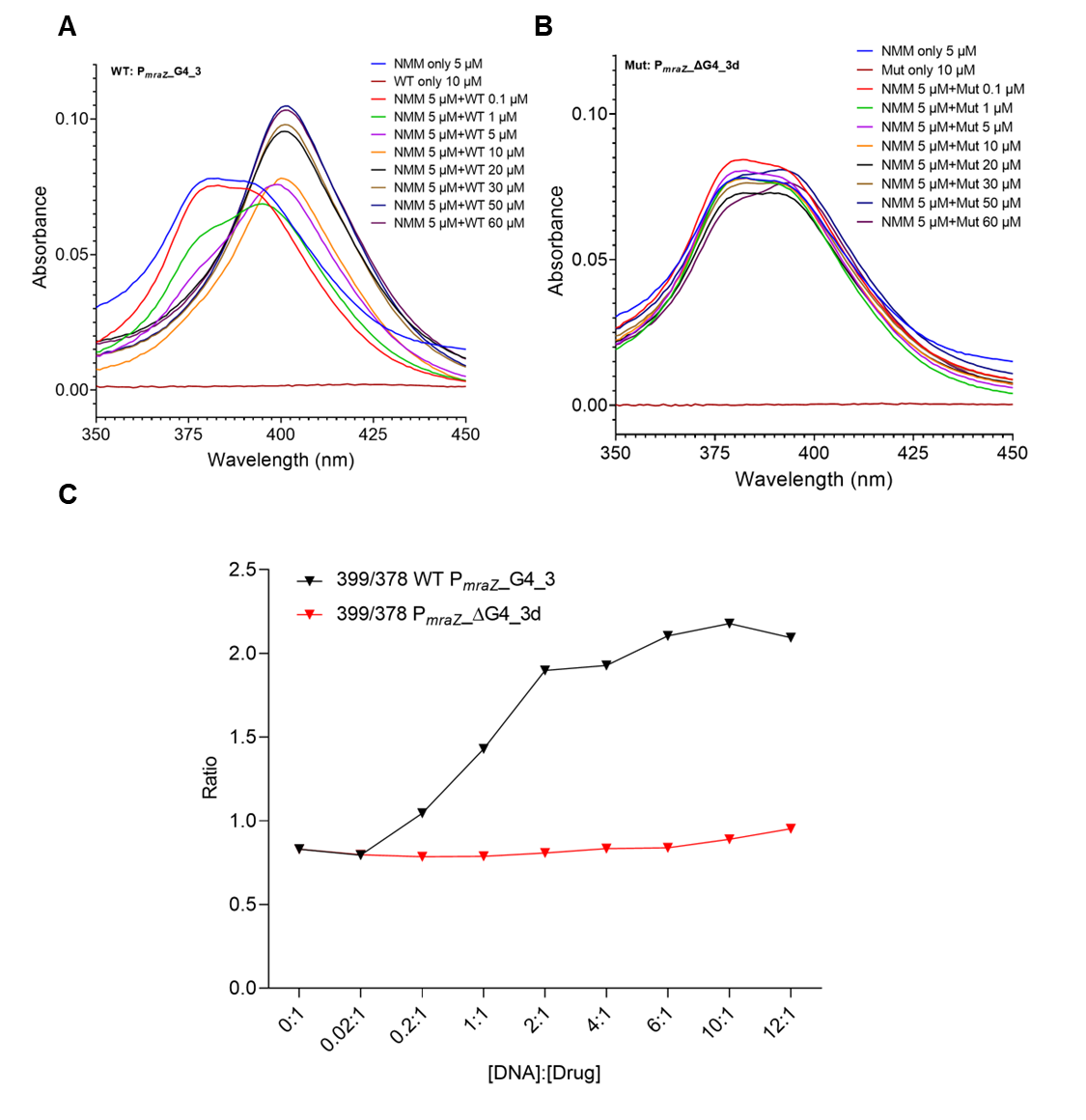


**Supporting Figure S9**. **UV-Visible titration of wild-type and mutant G4 sequences with NMM in solution. (A)** To investigate the interactions of NMM (Drug) with WT P*_mraZ_*_G4_3 G-quadruplex (DNA) in solution, UV-Visible titration assay was performed wherein NMM showed an absorption peak at 378 nm (λ_max_ = 378 nm) with a very low level of absorbance. However, as expected, a significant increase in absorbance occurred when NMM solution was mixed with the wild type P*_mraZ_*_G4_3 DNA. UV-Vis spectroscopic titrations of NMM in solution with P*_mraZ_*_G4_3 showed the interaction by showing the red shift of 21 nm with absorption maxima of 399 nm (λ_max_ = 399 nm) while the control mutant P*_mraZ_* ΔG4_3d DNA (**B**) did not show any red-shift indicating its non-interaction with NMM. (**C**) The increment in absorbance ratio (399/378 nm) of wild type P*_mraZ_*_G4_3 DNA against the ratio of [DNA]/[Drug] showed the specific interaction in solution as compared to the control mutant P*_mraZ __*ΔG4_3d.

**
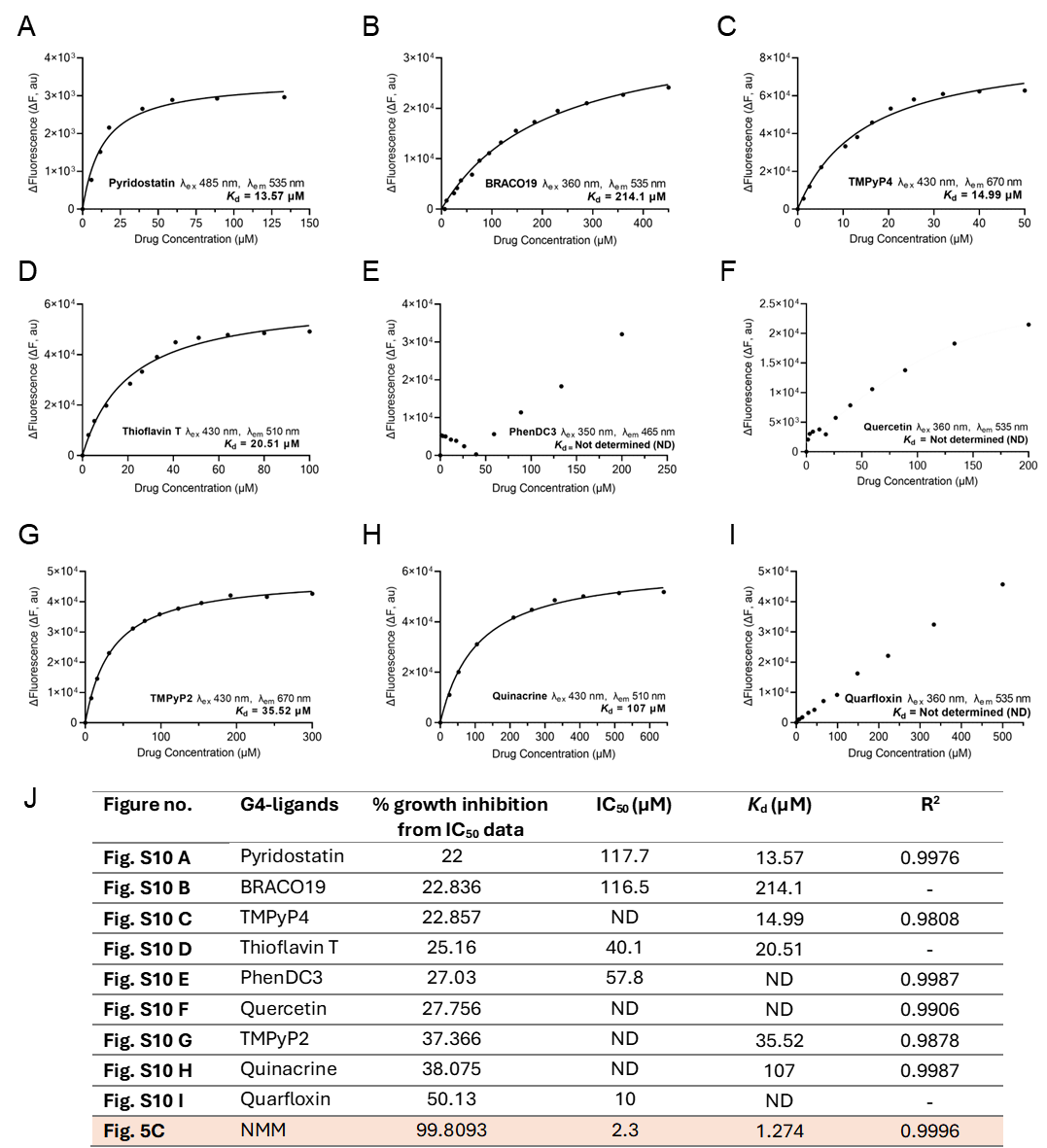
****Figure S10**. **Steady-state fluorescence titration assay showing the *K*_d_ values**. (**A**) Pyridostatin (PDS) (**B**) BRACO19, (**C**) TMPyP4, **(D)** Thioflavin T, **(E)** PhenDC3 (ND = not determined; unexpected drug concentrations versus fluorescence response) **(F)** Quercetin (ND; due to abrupt response up to 25 µM Quercetin concentrations versus fluorescence), **(G)** TMPyP2, **(H)** Quinacrine, **(I)** Quarfloxin (ND; due to lack of saturation point), and (**J**) Tabulated datasheet showing the *K*_d_ values with their IC_50_ and percent inhibition of SAUSA300 growth using G4-ligands at 10 µM derived from Chart diagram of Figure S2 K. Based on all the data points, NMM showed the maximum growth inhibition (99.8%) at the minimum IC_50_ (2.3 µM) with maximum internalization of drug **(Figure S3 H’**) and displayed the highest binding affinity (*K*_d_ 1.27 × 10^-6^ M) with WT P*_mraZ_*_G4_3 target G4-motif.


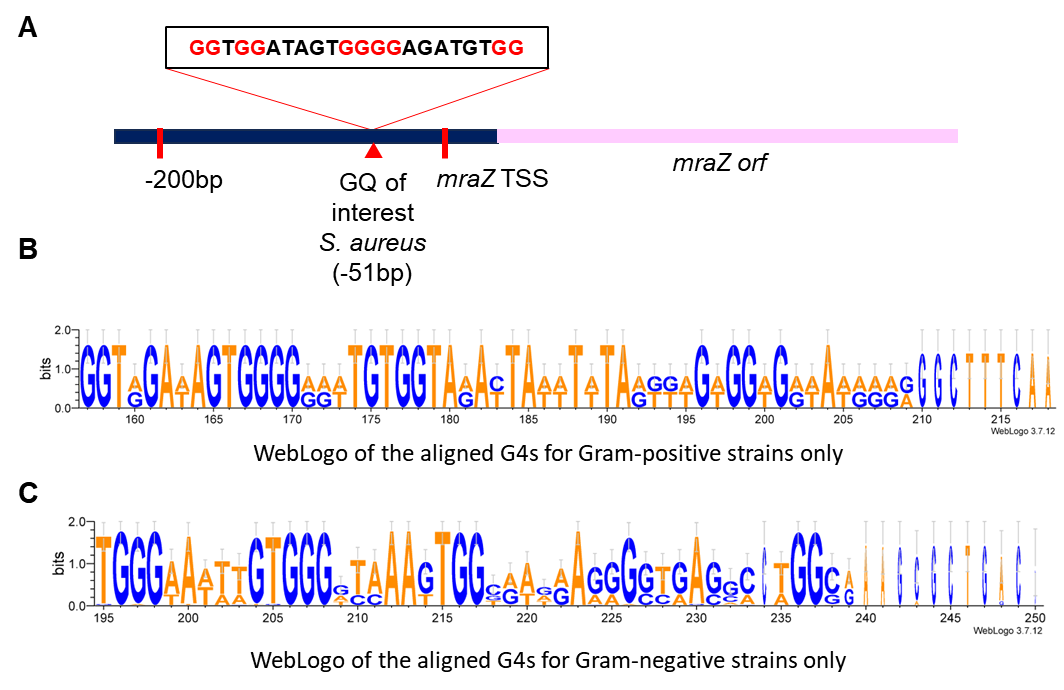
**Figure S11. Conservation of G4 regions in P*_mraZ_* promoter region of *dcw* cluster in various bacterial strains.** (**A**) Schematic diagram showing *mraZ* regulator with its -200 bp putative regulatory region including P*_mraZ_* promoter, with G4 motif and *mraZ* transcription start site (TSS) in SAUSA300 strain. (**B**) WebLogo of the aligned G4s for gram-positive strain only. (**C**) WebLogo of the aligned G4s for gram-negative strain only. This analysis indicates that the G4 motif in the regulatory region of *mraZ* is conserved both in gram-positive and -negative bacteria analyzed in this study shown in **Table S3**.


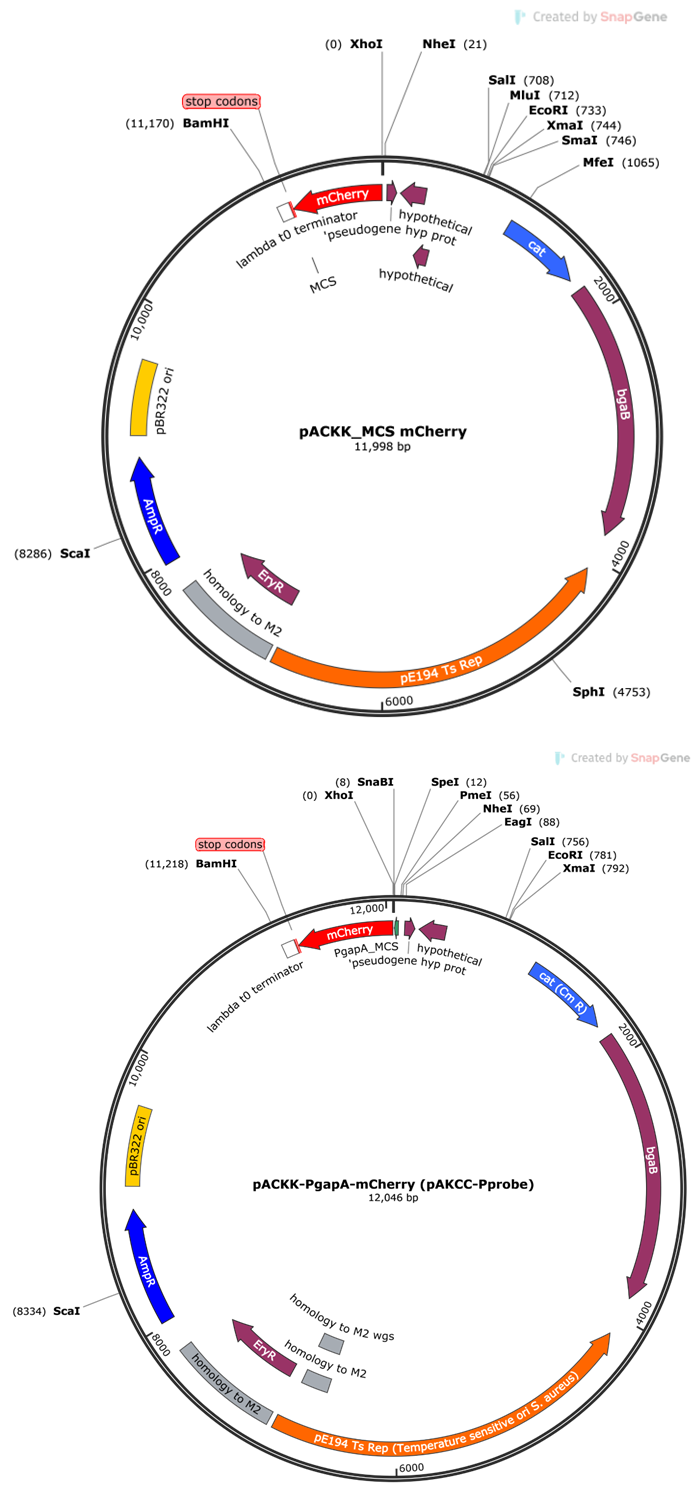


**Figure S12**. **Vector maps of promoter-less vector and pACKK_P*_Probe_* vector for *E. coli* and *S. aureus* strains. (A)** Promoter-less *mCherry* reporter gene with MCS to clone the promoter of interest showed no background or leaky expression of mCherry protein that can serve as no promoter control (negative) in the experiment; and (**B**) The vector map of pACKK_P*_Probe_* to replace the *P_gapA_* promoter with the promoter of interest with multiple options of restriction enzymes in the multiple cloning site. pACKK_P*_Probe_* vector showing a high-level of mCherry expression can be used as a positive control as a strong promoter to determine the comparative strength of the promoter of interest.


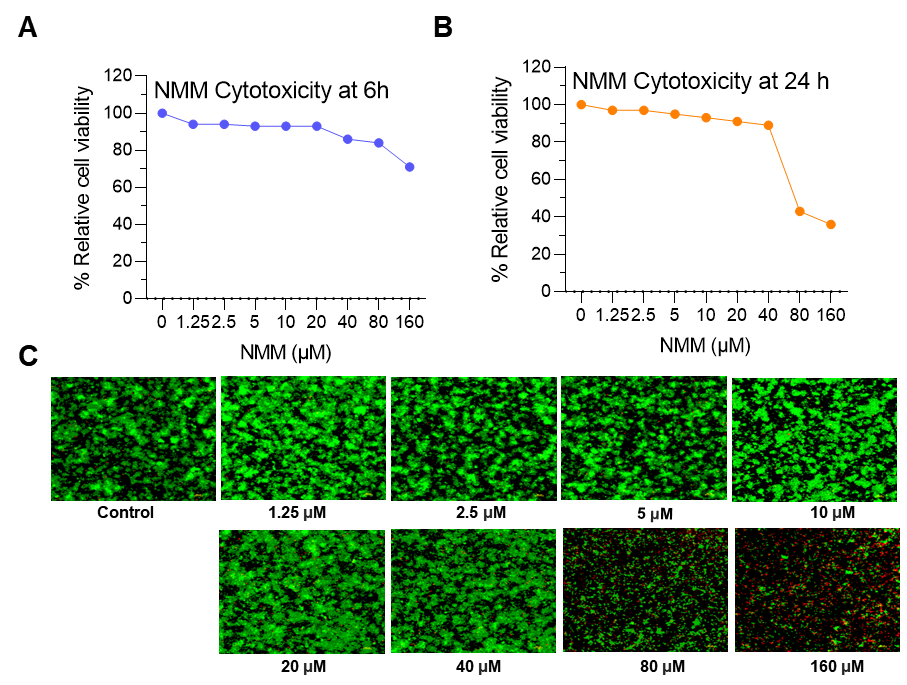


**Figure S13. Assessment of NMM cytotoxicity in RAW264.7 cells at 6 and 24 h**. The *in vitro* toxicity of NMM by percent relative cell viability of RAW264.7 cells was measured using the WST-8 cell viability kit at (**A**) 6h, (**B**) 24 h after treatment with NMM at varying concentrations. (**C**) Confocal microphotographs showing the RAW264.7 cells treated with varying concentrations of NMM for 24 h followed by staining with Acridine orange/Propidium Iodide (AO/PI live/dead stain). NMM appears to be safe up to 40 µM with merely ~15% toxicity after 24 h of treatment.


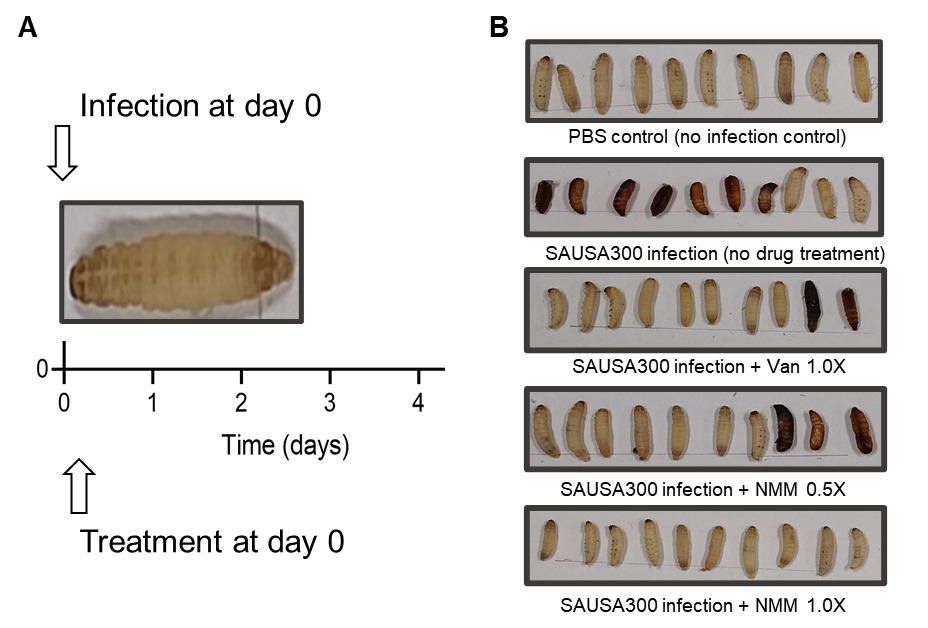


**Figure S14.** **SAUSA300 infection to waxworm.** (**A**) Timeline of infection of waxworm with SAUSA300 followed by treatment with 0.5 and 1X MICs of NMM. (**B**) 100% survival of waxworms at 1X MIC of NMM as compared to 30% survival of infection control. A last-resort glycopeptide antibiotic, vancomycin (Van) at 1X MIC was treated as a positive control of antibacterial treatment.


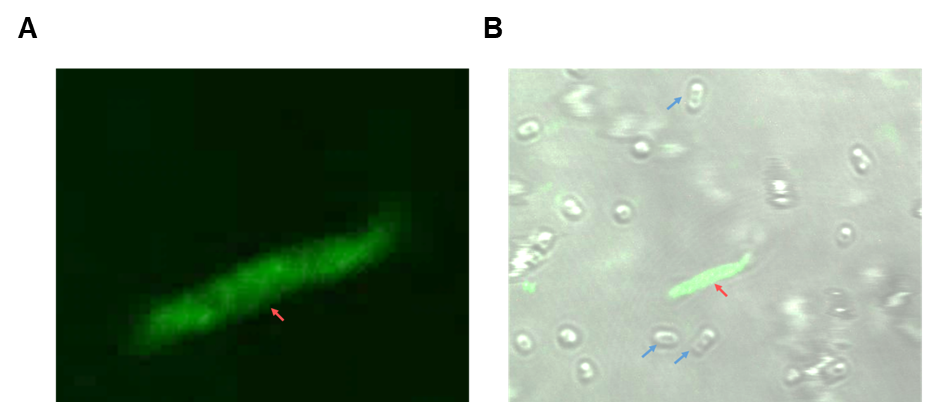


**Figure S15.** **Phenotypic assessment of *Escherichia coli*** **CFT073 cells treated with NMM**. (**A**) *E. coli* CFT073 cells were treated with 0.5X MIC of NMM for 2 h and cells were strained with Wheat germ agglutinin Alexa-fluor488 conjugate. No NMM internalization as red fluorescence was visualized presumably due to outer membrane permeability barrier. (**B**) Merged photomicrograph showing elongated *E. coli* CFT073 cells due to inhibition of cell division at sublethal concentration of NMM (red arrow) than those of normal *E. coli* CFT073 cells (blue arrow).

**
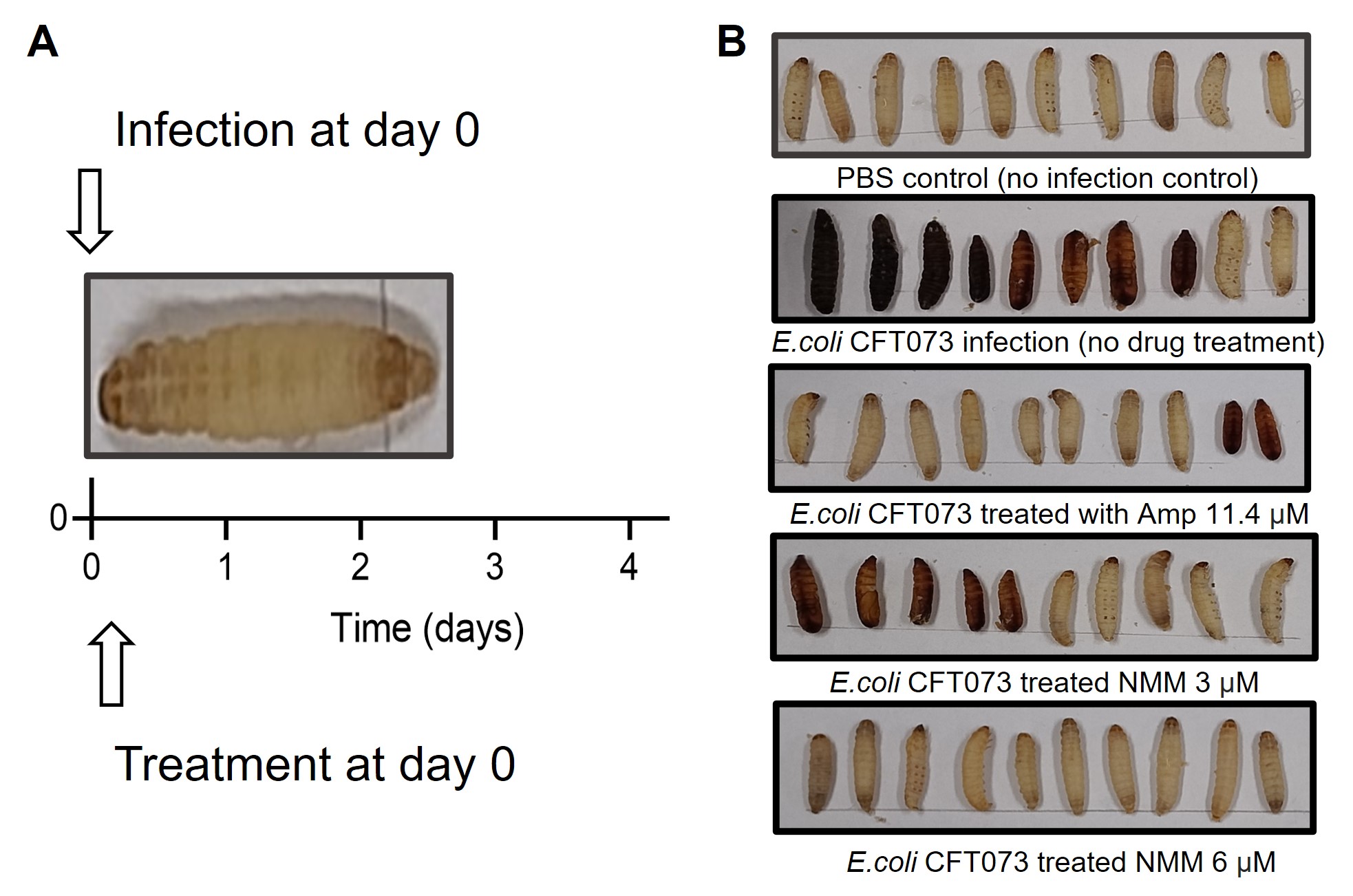
Figure S16.** **Uropathogenic *E. coli* CFT073 infection to waxworm.** (**A**) Timeline of infection of waxworm with *E. coli* CFT073 followed by treatment with 3 µM and 6 µM of NMM showing (**B**) 100% survival of waxworms at 6 µM of NMM as compared to 20% survival of infection control. A known antibiotic ampicillin was treated at 11.4 µM as a positive control of antibacterial treatment.


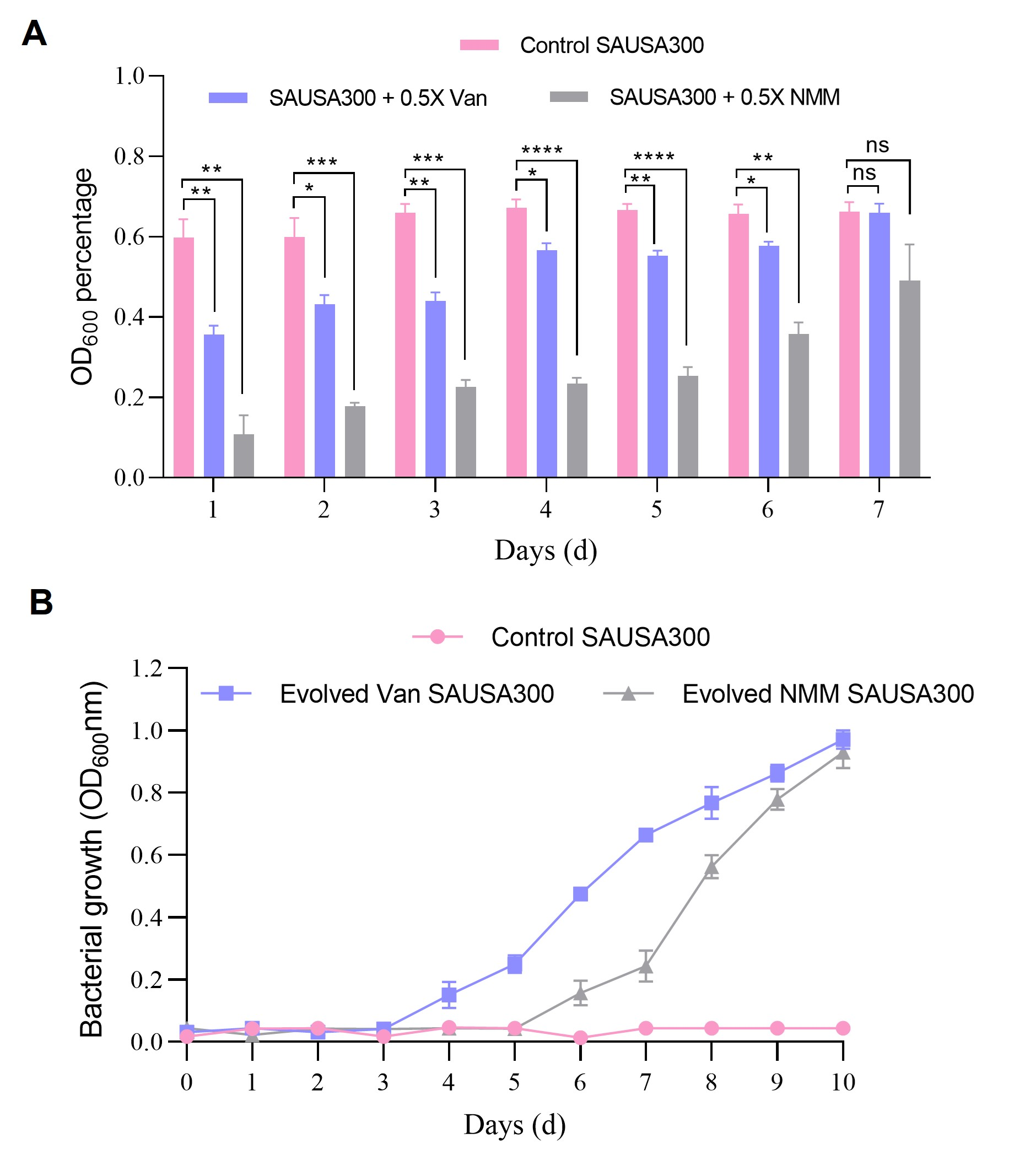


**Figure S17. Comparative assessment of resistance development upon sustained exposure of vancomycin or NMM. (A)** SAUSA300 growth at every 24 h intervals at the 0.5X MICs of vancomycin (Van) and NMM, and **(B)** Growth curves of the evolved strains challenged with 1.0X MIC of Van and NMM. The growth curve of control SAUSA300 strain which had never been exposed to NMM was kept as a control with 1.0X MIC of NMM (•)SAUSA300 evolved on day 4 in the presence of Van (■) and on day 6 in the presence of NMM (▲). Additionally, SAUSA300 growth rate remained significantly lower in NMM-evolved strain as compared to that in Van-evolved SAUSA300. However, the growth of NMM-evolved strain reached equal to Van-evolved strain on day 10 presumably due to emergence and clonal selection on day 6 followed by enrichment for the next 4 subcultures. These results suggest that the development of resistance rate is slower in NMM than that in Van.

**
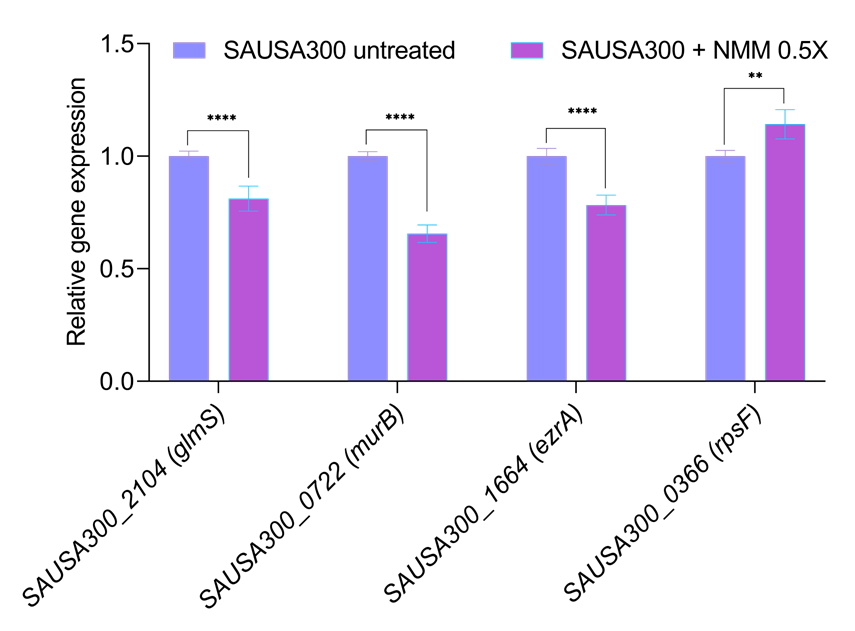
**

**Figure 18.** **Impact of NMM on four representative essential genes involved in cell-division and cell wall biosynthesis outside of *dcw* cluster in SAUSA300.** Assessment of 4 representative essential genes based on lethality of their mutants [3] *i.e.* [*rpsF* (30S ribosomal protein S6 [4]); *glmS* (glucosamine--fructose-6-phosphate aminotransferase, *glmS* riboswitch acts as drug-target for antibiotics development [5]); *murB* (UDP-N-acetylenolpyruvoylglucosamine reductase, [6]); and *ezrA* (septation ring formation regulator [7])] were assessed for gene expression without or with NMM exposure for 1h. These genes are not regulated by G4-motif and are involved in vital cellular functions such as ribonucleoprotein complex (*rpsF*), cell wall biosynthesis (*glmS* and *murB*), and cell division (*ezrA*). Three essential genes (*glmS*, *murB* and *ezrA*) were found to be downregulated with 0.5× NMM treatment for 1h suggesting that the NMM differentially regulates other cell-wall and cell-division related genes upon onset of bactericidal cascades presumably starting *via dcw* cluster.

**
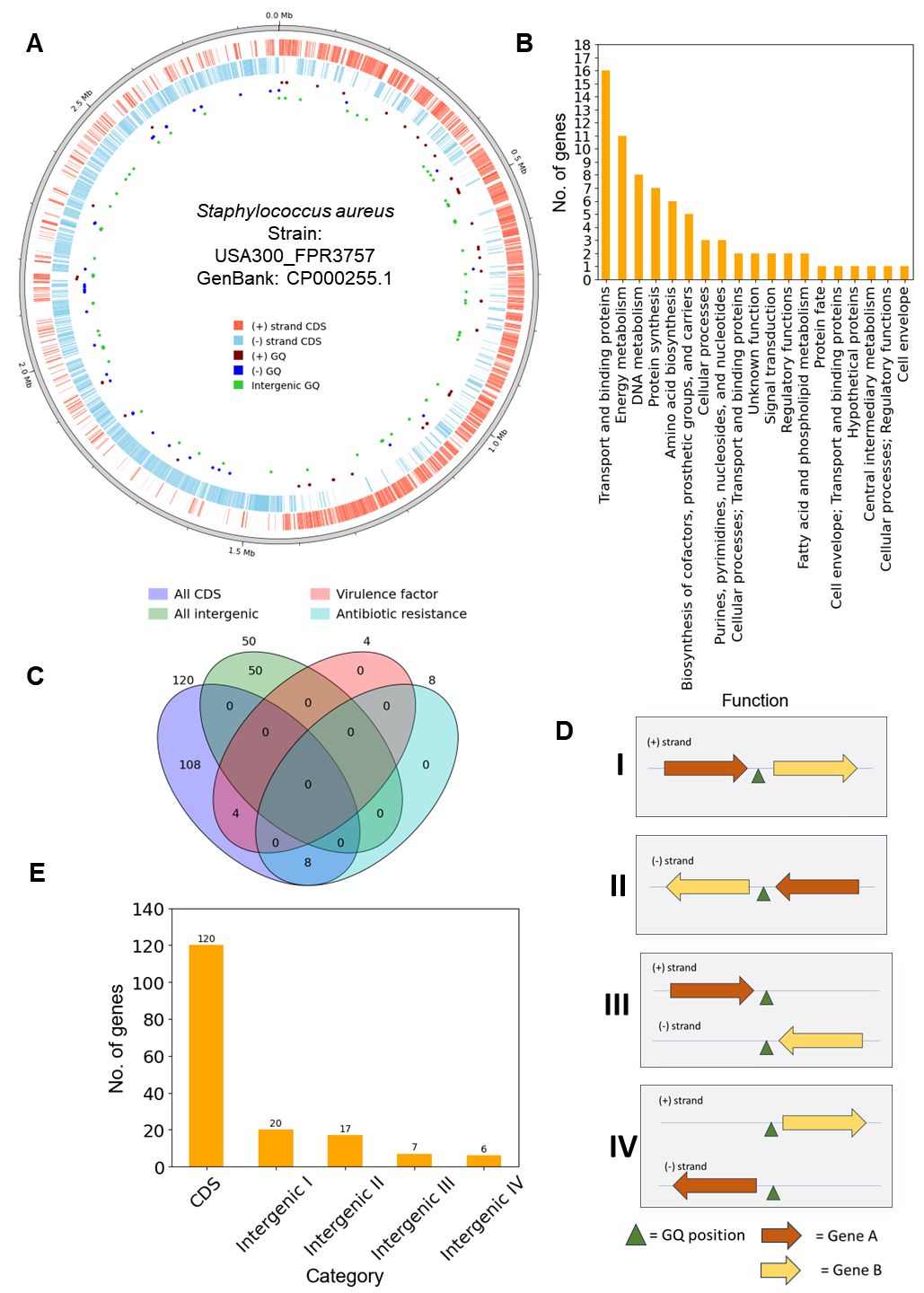
Figure S19.** **Genome-wide analysis of GQ/G4 motifs present in conserved domain sequences and intergenic regions**. (**A**) Genome plot showing the GQ/G4 motifs mapped on SAUSA300 genome. (**B**) Functional categories of GQ/G4-associated genes (based on TIGRFAM classification). (**C**) Venn diagram showing genes in CDS, intergenic regions and their association with virulence and antibiotic resistance. (**D**) Types of intergenic region showing (**I**) GQ/G4 sequences present on positive strand, (**II**) GQ/G4 sequences present on negative strand, (**III**) G4/GQ sequences present between two convergent genes and (**IV**) GQ/G4 motifs present between two divergently located genes. (**E**) Location-based distribution of GQ/G4 motifs in CDS or various intergenic regions.

**
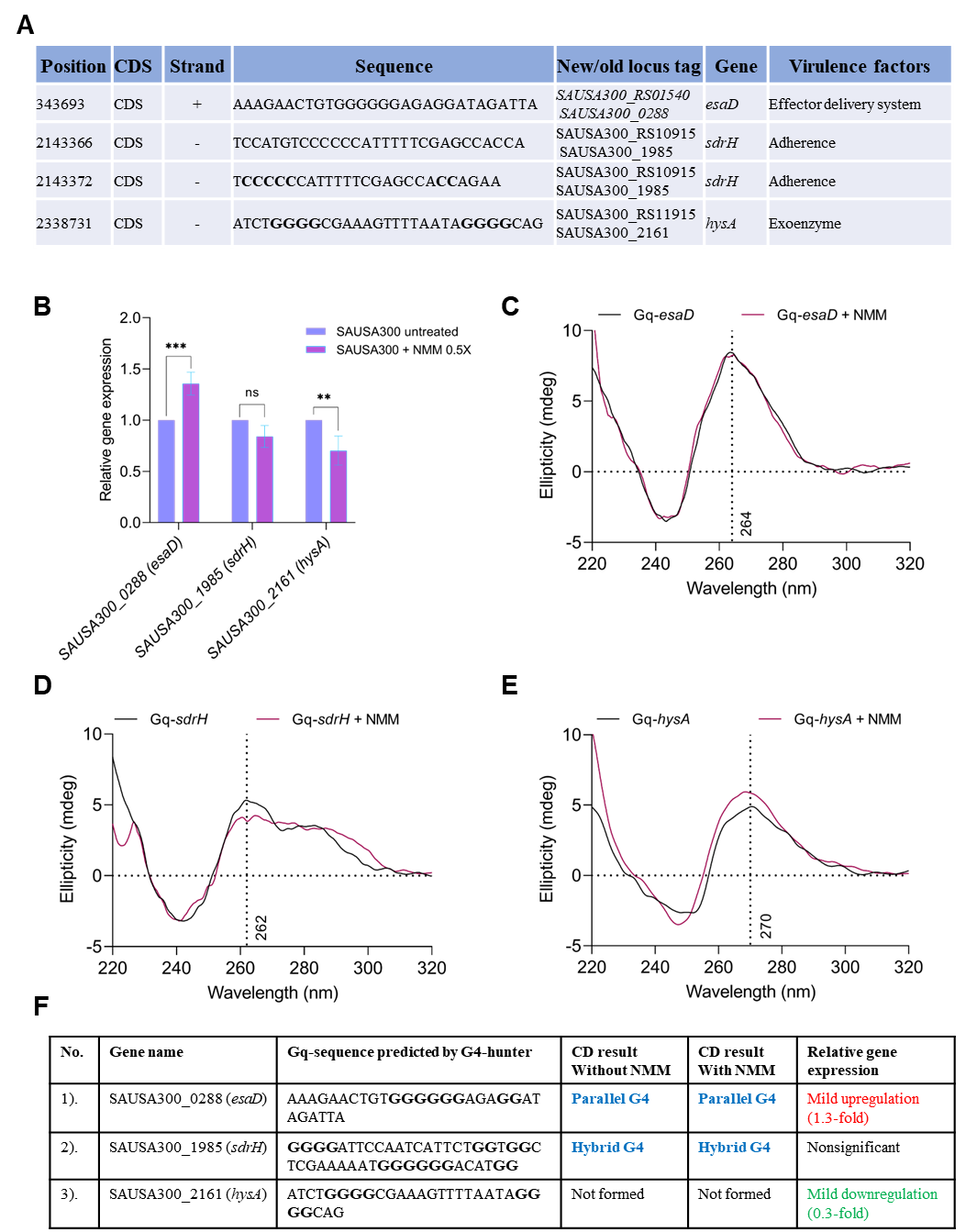
**

**Figure 20.** **G4 motifs present in virulence genes of SAUSA300 and their gene expressions analyses using qRT-PCR followed by CD spectral evaluation to validate G4 conformation of predicted putative G4s without or with NMM.** (**A**) List of virulence factors associated with G4 motif(s). (**B**). Gene expression analyses without or with NMM using qRT-PCR showed the mild upregulation of *esaD* and downregulation of *hysA* while there was no change in expression of *sdrH.* Significance was determined using Student's *t*-test, with *p*-values <0.05 deemed significant; ns (non-significant), *p*>0.05; and **p* < 0.05, ***p* < 0.01, ****p* < 0.005, and *****p* < 0.0001). (**C–E**) CD spectral analyses as a preliminary validation of predicted putative G4 motifs for their G4 conformation in *esaD* (**C**), *sdrH* (**D**) and *hysA* (**E**). (**F**) The tabulated datasheet showing the summary showing *esaD* and *sdrH* could formed as parallel and hybrid G4 conformations, respectively. The G4 conformation and the gene expression patterns of the virulence genes reveal the weak correlation between the NMM-mediated changes in gene expression and their G4 structures. The mild alteration in gene expression could be attributed to nonspecific/indirect effect of the antibacterial activity of NMM.


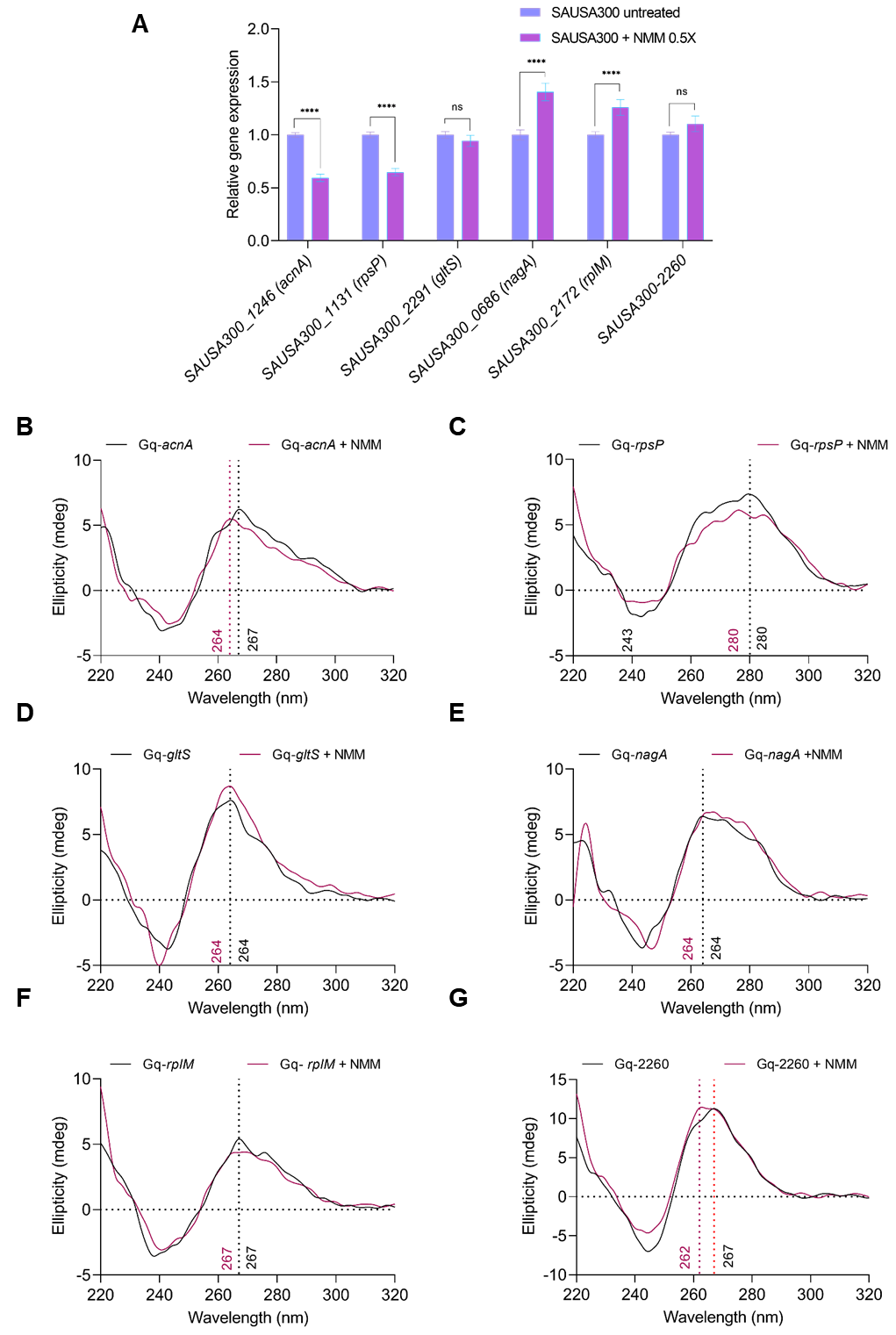


**
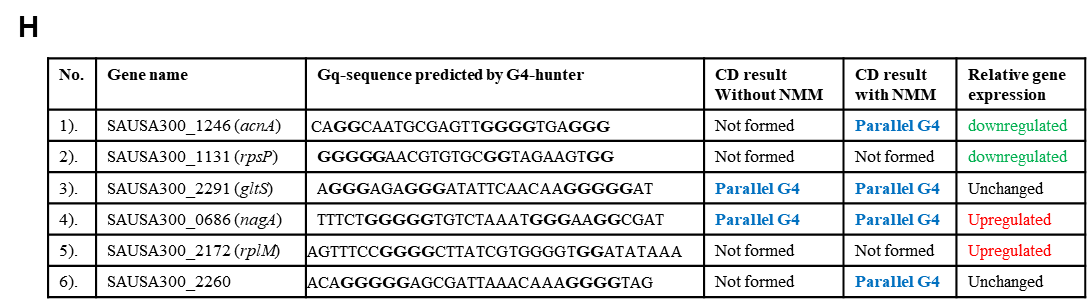
****Figure 21. G4 motifs in the promoter of representative essential and non-essential genes and their gene expressions analyses using qRT-PCR followed by CD spectral analyses to validate G4 conformation of the predicted putative G4s without or with NMM.** (**A**). Gene expression analyses without or with NMM using qRT-PCR showing differential expression wherein the aconitate hydratase encoding gene (*acnA*) and 30S ribosomal protein S16 (*rpsP*) was found to be downregulated while N-acetylglucosamine-6-phosphate deacetylase (*nagA*) and 50S ribosomal protein L13 (*rplM*) were mildly upregulated. There was no effect on gene expression of sodium/glutamate symporter (*gltS*) and inositol monophosphatase family protein (*SAUSA300_2260*). Significance was determined using Student's *t*-test, with *p*-values <0.05 deemed significant; ns (non-significant), *p*>0.05; and **p* < 0.05, ***p* < 0.01, ****p* < 0.005, and *****p* < 0.0001). (**B–G**) CD Spectro-polarimetric preliminary assessment of predicted putative G4 motifs in *acnA* (**B**), *rpsP* (**C**), *gltS* (**D**), *nagA* **(E)**, *rplM* (**F**), and *SAUSA300_2260* (**G**). (**H**) The tabulated datasheet showing the summary G4 conformations and relative gene expression, respectively. The preliminary validation of G4 conformation of these representative G4 motifs present in the promoters and the mild modulation in their gene expression levels did not show any correlation between the G4 conformation and NMM mediated gene regulation. These results suggest that the mild modulations in the gene expression of these genes are due to antibacterial impacts of NMM rather than the NMM’s specific targeting to these G4 and consequent gene regulation.

**SUPPLEMENTARY TABLE S1. Bacterial strains used in the study**

| Strains | Descriptions | Reference/Source |
| --- | --- | --- |
| *Staphylococcus aureus* USA300 | JE2, wild-type epidemic community-associated methicillin-resistant *S. aureus* isolate USA300 LAC. | NARSA [3] |
| *Eschericha coli* CFT073 | Pyelonephritis isolate, *fim+ pap+ hly+* | [8] |
| *Eschericha coli* DH5α | *F- end*A1 *gln*V44 *thi*-1 *rec*A1 *rel*A1 *gyr*A96 *deo*R *nup*G Φ80d *lacZ*ΔM15*Δ(lac*ZYA*-arg*F*) U*169*, hsdR17 (rK- mK+), λ–* | Laboratory strain |
| *E. coli* DH5α with *pRP1195* | *E. coli* DH5α with pRP1195 expression *luxBADCE* under P*_gapA_* promoter | [9] |
| *E. coli* DH5α with *pRP1195* minus P*_gapA_*-*luxBADCE* | *Eag*I-*Eag*I cloned P*_gapA-_luxBADCE* deleted to create vector backbone | This Study |
| *E. coli* DH5α with *pACKK*-P*_gapA_*-MCS | Synthetic MCS with P*_gapA_* promoter cloned at *Eag*I*-Bam*H1 sites | This Study |
| *E. coli* DH5α with *pACKK*-P*_gapA_*-*mCherry* (*pACKK*-P*_probe_*) | mCherry protein expressing under P*_gapA_* promoter; *amp^r^* and *cm^r^* | This study |
| *E. coli* DH5α with *pACKK*-*mCherry* | Promoter-less vector with *mCherry* (empty vector, EV); *amp^r^* and *cm^r^* | This study |
| *E. coli* DH5α with *pACKK*-P*_mraZ_*-*mCherry* | mCherry expressing under P*_mraZ_* promoter; *amp^r^* and *cm^r^* | This study |
| *E. coli* DH5α with *pACKK*-P*_mraZ_*_-mut_-*mCherry* | mCherry expressing under site-directed mutation (SDM) of P*_mraZ__*_mut_ promoter with destabilized G4 motif of P*_mraZ_* promoter; *amp^r^* and *cm^r^* | This study |
| *E. coli* DH5α with *pACKK*-P*_gapA_-mraZ-_his6_*-*mCherry* | Recombinant MraZ_-His6_ protein translationally fused with mCherry protein under P*_gapA_* promoter; *amp^r^* and *cm^r^* | This study |
| *E. coli* DH5α with *pACKK*-P*_mraZ_*-*mraZ*-*_his6_*-*mCherry* | Recombinant MraZ_-His6_ protein translationally fused with mCherry protein under P*_mraZ_* promoter; *amp^r^* and *cm^r^* | This study |

SUPPLEMENTARY TABLE S2. Primers used in the study

| **Primer name** | **Primer sequence (5’-3’)** |
| --- | --- |
| **Primers used for qRT-PCR** | |
| ***dcw cluster related genes*** | |
| ***mraZ-fwd*** | CGTCCAAGTTTCGTTATGA |
| ***mraZ-rev*** | AGTTCTACTTCAACAGCACC |
| ***mraW-fwd*** | TCAAGACCAAACTGCAATTG |
| ***mraW-rev*** | TTCTGGAATGTCGAGTTGTG |
| ***murD-fwd*** | GTATTAGTTGTCGGTTTGGC |
| ***murD-rev*** | CACTTACAACAGAAATGCCC |
| ***ftsL-fwd*** | AGTATACCGAAGCAACAACC |
| ***ftsL-rev*** | ATCTGCAATCTTTCCTCGC |
| ***ftsA-fwd*** | GGATACAGAAATCAACGGTTC |
| ***ftsA-rev*** | GCCTGCTTCAACCTTTAATG |
| ***ftsZ-fwd*** | TATCGCTATCAACACAGACG |
| ***ftsZ-rev*** | AATACCATGTCTGCACCTTG |
| ***Housekeeping gene*** | |
| ***gyrA-fwd*** | ATTGAAGGTCCTGATTTCCC |
| ***gyrA-rev*** | TACGAGCCTTATTCACTTGG () |
| ***Virulence related genes*** | |
| ***esaD*-fwd** | GTAATAGCCATGCACAAAGAAC |
| ***esaD*-rev** | AACTCTTGCCACTCTTTCTC |
| ***sdrH*-fwd** | GGGCAAAGATTATGGAGAAGTT |
| ***sdrH*-rev** | CACCAGTAATTAATGCGAGTAC |
| ***hysA*-fwd** | TAAAGGCGGAAATCTAGTAGAC |
| ***hysA*-rev** | GTATGGAACGTCTTGATGATCA |
| ***G4 motifs present in gene’s promoter*** | |
| ***acnA-fwd*** | GACAACTGACCACATCTCTC |
| ***acnA-rev*** | AAACCACCTTCAGTACCTG |
| ***nagA-fwd*** | AGTGGATGATAAAGCAGCAA |
| ***nagA-rev*** | TATGATGTCGTCCCTTCAGA |
| ***rpsP-fwd*** | GCAGTTAAAATTCGTTTAACACG |
| ***rpsP-rev*** | GTTTCGCACCATCATTTAACC |
| ***rplM-fwd*** | GTAGCATCTATCTTACGCGG |
| ***rplM-rev*** | GTTCTTCTTAATTCACCAGCAG |
| ***gltS-fwd*** | GGTATTTTCTTATCTCTTGCGC |
| ***gltS-rev*** | AGCCCATGACCGATAAAAC |
| ***SAUSA2260-fwd*** | ATCGTTAAAATTGGAAGACGC |
| ***SAUSA2260-rev*** | AGCAATGTCCCAAGGTTTAG |
| ***Essential genes*** | |
| ***glmS-fwd*** | GCAGGTATCGCAGTAGTAAA |
| ***glmS-rev*** | AAACGGCCATTTGATGATTG |
| ***murB-fwd*** | AGTGTATATGAATGCTGGCG |
| ***murB-rev*** | CAGTCATTTTACCAGGAGCT |
| ***ezrA-fwd*** | ATTTGAGCCAAGGTTAGAGC |
| ***ezrA-rev*** | AGATCACGGCAACCATATTT |
| ***rpsF-fwd*** | GCCCAAACATTGAGGAAGAT |
| ***rpsF-rev*** | TCGTCAGTAGCTTTGTTGTT |
| ***Primers used for cloning and amplification of templates for coupled in vitro transcription/translation (IVT)*** | |
| ***mCherry_XhoI_Fwd*** | AATAATCTCGAGATGGTAAGCAAGGGCGAGGAGG |
| ***mCherry BamHI_Rev*** | AATAAAggatcCATTCTCACCAATAAAAAACGCCCGG |
| ***Gib_P_mraZ__Fwd*** | CTTAAGGGGCCCGCTAGCCCACCTATCCACCACTTTC |
| ***Gib_P_mraZ__Rev*** | CATCTCGAGTACGTAACTAGTtttttatcacctcaccttatatataat |
| ***Mut_ P_mraZ__Fwd*** | CaaattttgtataaatagtggtgAatagtAgAgagatgtgGtaaattatatataaggt |
| ***Mut_ P_mraZ__Rev*** | ACCTTATATATAATTTACCACATCTCTCTACTATTCACCACTATTTATACAAAATTTG |
| ***P_mraZ_-Fuse_Fwd*** | GCCTTAAGGGGCCCGCTAGCtccacctatccaccactttcttac |
| ***mraZ-orf-Fuse-Fwd*** | GGTTTGTGTTATAATACTAGTATGTTCATGGGAGAATACGATC |
| ***mraZ-NS-his6_Rev*** | CGCCCTTGCTCACCATCTCGAGGTGGTGATGGTGATGATGaaaatcaaaatctattaaatc |
| ***T7+GQ_P_mraZ__Fwd*** | TAATACGACTCACTATAGGgttttttaaaataaatttcacaaattttg |
| ***T7-GQ_P_mraZ__Fwd*** | TAATACGACTCACTATAGGtatataaggtgaggtgataaaa |
| ***mraZ-his_Stop-Rev*** | TTACTCGAGGTGGTGATGGTGATGAT |

**SUPPLEMENTARY TABLE S3**. Gram-positive and gram-negative bacterial strains used for conservation of putative G4-motifs in P*_mraZ_* promoters (Figure S11 and Fig. S6A) with their respective GenBank ID and Assembly ID.

| **Strain** | **GenBank ID** | **Assembly ID** |
| --- | --- | --- |
| *Enterococcus faecium* strain SRR24 | CP038996.1 | GCA_009734005.2 |
| *Enterococcus faecium* strain AA622 | AP024837.1 | GCA_019977575.1 |
| Enterococcus faecium strain UCH1 | CP096211.1 | GCA_023204955.1 |
| *Enterococcus faecium* strain 2016C08-243 | CP131809.1 | GCA_038439085.1 |
| *Enterococcus faecium* strain VRE001 | CP018071.1 | GCA_001895905.1 |
| *Enterococcus faecium* strain AML0157 | CP060861.1 | GCA_014490015.1 |
| *Enterococcus faecium* strain DVT1681 | CP112862.1 | GCA_026409165.1 |
| *Enterococcus faecium* strain CFSAN059071 | CP020488.1 | GCA_003071445.1 |
| *Enterococcus faecium* strain RBWH1 | CP033206.1 | GCA_003957785.1 |
| *Enterococcus faecium* strain A6521 | CP061817.1 | GCA_012933195.2 |
| *Staphylococcus aureus* subsp. *aureus* USA300_FPR3757 | CP000255.1 | GCA_000013465.1 |
| *Staphylococcus aureus* strain MRSA107 | CP018629.1 | GCA_002895385.1 |
| *Staphylococcus aureus* strain KWT 2020-26218 | CP146210.1 | GCA_037039295.1 |
| *Staphylococcus aureus* strain NY2491 | CP094855.1 | GCA_022832755.1 |
| *Staphylococcus aureus* strain HC1340 | CP012011.1 | GCA_001515745.1 |
| *Staphylococcus aureus* strain M92 | CP015447.2 | GCA_002097595.2 |
| *Staphylococcus aureus* strain CMRSA-3 | CP029685.1 | GCA_003264775.1 |
| *Staphylococcus aureus* strain HL21008 | CP080562.1 | GCA_019551095.1 |
| *Staphylococcus aureus* strain SA191 | CP133442.1 | GCA_031190615.1 |
| *Staphylococcus aureus* strain PNID0137 | CP071594.1 | GCA_018399115.1 |
| *Klebsiella pneumoniae* strain KP2722 | CP116903.1 | GCA_028471925.1 |
| *Klebsiella pneumoniae* strain QD23 | CP042858.1 | GCA_011045595.1 |
| *Klebsiella pneumoniae* strain TH12908 | CP087123.1 | GCA_020911805.1 |
| *Klebsiella pneumoniae* strain KPNIH50 | CP026177.1 | GCA_002903005.1 |
| *Klebsiella pneumoniae* strain WCGKP294 | CP046612.1 | GCA_009755705.1 |
| *Klebsiella pneumoniae* strain 2020N06-159 | CP129814.1 | GCA_030490145.1 |
| *Klebsiella pneumoniae* strain CRKP-F1 | CP132042.1 | GCA_030717725.1 |
| *Klebsiella pneumoniae* strain KP29105 | CP091061.1 | GCA_021535085.1 |
| *Klebsiella pneumoniae* strain ST3576 | CP103697.1 | GCA_024918475.1 |
| *Klebsiella pneumoniae* strain S24_CRE24 | CP074541.1 | GCA_027595685.1 |
| *Pseudomonas aeruginosa* strain TY922 | CP155639.1 | GCA_039702325.1 |
| *Pseudomonas aeruginosa* strain CH1 | CP144289.1 | GCA_036542105.1 |
| *Pseudomonas aeruginosa* strain 34Pae36 | CP095770.1 | GCA_023093935.1 |
| *Pseudomonas aeruginosa* strain 2021CK-01162 | CP124632.1 | GCA_029961325.1 |
| *Pseudomonas aeruginosa* strain F30658 | CP008857.1 | GCA_001516265.1 |
| *Pseudomonas aeruginosa* strain NY5524 | CP096942.1 | GCA_030121935.1 |
| *Pseudomonas aeruginosa* strain P9W | CP081202.1 | GCA_019710495.1 |
| *Pseudomonas aeruginosa* strain LRJ32 | CP115191.1 | GCA_036232065.1 |
| *Pseudomonas aeruginosa* strain 2857 | CP116717.1 | GCA_028404045.1 |
| *Pseudomonas aeruginosa* strain F024 | CP115262.1 | GCA_036233355.1 |
| *Escherichia coli* strain MFDS1006657 | CP073589.1 | GCA_018223665.1 |
| *Escherichia coli* strain EC-10 | CP065203.1 | GCA_039725815.1 |
| *Escherichia coli* DSM 30083 = JCM 1649 = ATCC 11775 | CP033092.2 | GCA_003697165.2 |
| *Escherichia coli* strain 14EC020 | CP024138.1 | GCA_002853715.1 |
| *Escherichia coli* LF82 | CU651637.1 | GCA_000284495.1 |
| *Escherichia coli* strain ETEC1738 | CP122768.1 | GCA_030013595.1 |
| *Escherichia coli* strain ERL04-3476 | CP032808.1 | GCA_008931135.1 |
| *Escherichia coli* UTI89, | CP000243.1 | GCA_000013265.1 |
| *Escherichia coli* strain E2865 | AP018808.1 | GCA_003966465.1 |
| *Escherichia coli* ETEC H10407, | FN649414.1 | GCA_000210475.1 |
| *Enterobacter ludwigii* strain EN-119 | CP017279.1 | GCA_001750725.1 |
| *Enterobacter roggenkampii* strain L3897 | CP155692.1 | GCA_039727605.1 |
| *Enterobacter huaxiensis* strain 090008 | CP043342.1 | GCA_003594935.2 |
| *Enterobacter cloacae* strain FDAARGOS 1431 | CP077211.1 | GCA_019047105.1 |
| *Enterobacter cancerogenus* strain FDAARGOS 1428 | CP077290.1 | GCA_019047785.1 |
| *Enterobacter hormaechei* strain Eho-E1 | CP066094.1 | GCA_022023895.1 |
| *Enterobacter kobei* JCM 8580 DNA, | AP024590.1 | GCA_018323985.1 |
| *Enterobacter bugandensis* strain FDAARGOS 1427 | CP077206.1 | GCA_019046905.1 |
| *Enterobacter mori* strain ACYC.E9L | CP091779.1 | GCA_022014715.1 |
| *Enterobacter oligotrophicus* strain CCA6 | AP019007.1 | GCA_009176645.1 |

**SUPPLEMENTARY** TABLE S4. Preliminary screening of antibacterial properties of 10 representative G4-binding ligands against SAUSA300.

| **S. No.** | **Drug** | **% OD inhibition (SAUSA300) ^#^** |
| --- | --- | --- |
| 1 | Quinacrine | 47.2 |
| 2 | PhenDC3 | 56.6 |
| 3 | PDS | 58.9 |
| 4 | Quercetin | 60.6 |
| 5 | Quarfloxin | 60.6 |
| **6** | TMPyP4 | 61.8 |
| **7** | Thioflavin T | 62.3 |
| **8** | TMPyP2 | 67.0 |
| **9** | BRACO19 | 67.1 |
| **10** | NMM | 92.4 |

^#^In primary screening in microtiter plate assay, all the 10 ligands mentioned along with % inhibition of SAUSA300 in terms of OD_600nm_ at 10 µM. The G4-ligands from 6 to 10 (bold serial numbers) were further tested under optimal microbial growth conditions and by enumerating the colony forming units (CFU).

**References**

[1] Kowalska-Krochmal B, Dudek-Wicher R. The minimum inhibitory concentration of antibiotics: Methods, interpretation, clinical relevance. Pathogens. 2021;10:165.

[2] Romera C, Bombarde O, Bonnet R, Gomez D, Dumy P, Calsou P, et al. Improvement of porphyrins for G-quadruplex DNA targeting. Biochimie. 2011;93:1310-7.

[3] Fey Paul D, Endres Jennifer L, Yajjala Vijaya K, Widhelm Todd J, Boissy Robert J, Bose Jeffrey L, et al. A genetic resource for rapid and comprehensive phenotype screening of nonessential *Staphylococcus aureus* genes. mBio. 2013;4:10.1128/mbio.00537-12.

[4] Carter AP, Clemons WM, Brodersen DE, Morgan-Warren RJ, Wimberly BT, Ramakrishnan V. Functional insights from the structure of the 30S ribosomal subunit and its interactions with antibiotics. Nature. 2000;407:340-8.

[5] Traykovska M, Popova KB, Penchovsky R. Targeting *glmS* ribozyme with chimeric antisense oligonucleotides for antibacterial drug development. ACS Synth Biol. 2021;10:3167-76.

[6] Zhou J, Cai Y, Liu Y, An H, Deng K, Ashraf MA, et al. Breaking down the cell wall: Still an attractive antibacterial strategy. Front Microbiol. 2022;13.

[7] Haeusser Daniel P, Garza Anna C, Buscher Amy Z, Levin Petra A. The division inhibitor EzrA contains a seven-residue patch required for maintaining the dynamic nature of the medial FtsZ ring. J Bacteriol 2007;189:9001-10.

[8] Kim H, Chaurasia AK, Kim T, Choi J, Ha SC, Kim D, et al. Structural and functional study of ChuY from *Escherichia coli* strain CFT073. Biochem Biophys Res Commun. 2017;482:1176-82.

[9] Plaut RD, Mocca CP, Prabhakara R, Merkel TJ, Stibitz S. Stably luminescent *Staphylococcus aureus* clinical strains for use in bioluminescent imaging. PLoS One. 2013;8:e59232.
